# Interfacing B‐DNA and DNA Mimic Foldamers

**DOI:** 10.1002/anie.202505273

**Published:** 2025-06-17

**Authors:** Manuel Loos, Felix Xu, Pradeep K. Mandal, Tulika Chakrabortty, Céline Douat, David B. Konrad, Melis Cabbar, Johannes Singer, Valentina Corvaglia, Thomas Carell, Ivan Huc

**Affiliations:** ^1^ Department of Pharmacy Ludwig‐Maximilians‐Universität München Butenandtstr. 5–13 München 81377 Germany; ^2^ Department of Chemistry Ludwig‐Maximilians‐Universität München Butenandtstr. 5–13 München 81377 Germany; ^3^ Present address: Institute of Science and Technology Austria, Am Campus 1 Klosterneuburg 3400 Austria; ^4^ Present address: Department of Pharmaceutical Sciences, Universität Wien, Josef‐Holaubek‐Platz 2 Vienna 1090 Austria; ^5^ Present address: Institute for Stem‐Cell Biology, Regenerative Medicine and Innovative Therapies, IRCCS Casa Sollievo della Sofferenza, San Giovanni Rotondo (Italy) & Center for Nanomedicine and Tissue Engineering (CNTE), ASST Grande Ospedale Metropolitano Niguarda, Milan Rotondo Italy

**Keywords:** Chimeric molecules, DNA hairpin, DNA mimic foldamer, Helical molecules, Transcription factor

## Abstract

A linker unit was designed and synthesized that can serve both as a hairpin turn in a DNA duplex and anchor point for an aromatic helical foldamer mimicking the shape and surface properties of B‐DNA. Methods were developed to synthesize natural/non‐natural chimeric molecules combining foldamer and DNA segments. The ability of the linker to position the foldamer helix and the duplex DNA so that their rims and grooves are in register, despite their completely different chemical nature, was demonstrated using single crystal X‐ray diffraction, circular dichroism and molecular models. Bio‐layer interferometry confirmed that artificial hairpin DNA duplexes keep their ability to bind to DNA binding proteins. The chimeric molecules may pave the way to competitive inhibitors of protein‐DNA interactions involving sequence‐selective DNA‐binding proteins.

## Graphical abstract

Helical aromatic oligoamide DNA mimic foldamers that reproduce the shape and charge distribution of duplex B‐DNA have been interfaced with DNA duplexes by means of a new linker that serves both as a DNA hairpin and as an aromatic oligoamide unit. In the resulting chimeric molecules, the linker does not perturb the DNA duplex structure and converts the chirality of DNA into handedness control of the foldamer helix.

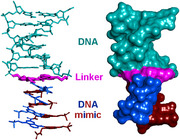

DNA mimic foldamers are single stranded aromatic helices having an overall shape and charge distribution similar to those of B‐DNA duplexes. Oligoamides composed of alternating M and Q quinoline‐derived amino acids (Figure 1a,b) constitute a prototypical example.^[^ ^1^, ^2^ ^]^ Like DNA mimic proteins^[^ ^3^, ^4^ ^]^ and some anionic polymers, e.g. heparin, DNA mimic foldamers may recognize DNA‐binding proteins with high affinity and show potential for interfering with some DNA‐protein interactions.^[^ ^1^, ^5^, ^6^, ^7^, ^8^ ^]^ Nevertheless, (MQ)_n_ oligoamides consist of a simple repeat motif and lack sequence features. They are thus less well suited to target proteins that recognize DNA sequence‐selectively such as transcription factors or restriction enzymes. A possible approach to overcome this limitation would be to combine an unnatural (MQ)_n_ oligoamide with natural DNA in a chimeric helical molecule that may benefit from the properties of both worlds, taking inspiration from other hybrid sequences that blend distinct molecular backbones, e.g. DNA‐peptide nucleic acid chimeras^[^ ^9^, ^10^, ^11^, ^12^, ^13^, ^14^, ^15^, ^16^ ^]^ and peptides that integrate α‐amino acids with some of their homologues^[^ ^17^, ^18^, ^19^, ^20^ ^]^ or analogues.^[^ ^21^, ^22^ ^]^ However, creating a DNA‐(MQ)_n_ interface that would allow for a structurally consistent arrangement of the DNA and foldamer subcomponents is challenging because of their completely distinct chemical nature and conformational behavior. Here, we report how we successfully reached this milestone through the design and synthesis of a linker that can serve as both a B‐DNA duplex hairpin turn and a single helical (MQ)_n_ extension, thereby placing the grooves and arrays of negative charges of the two segments in register.

**Figure 1:**
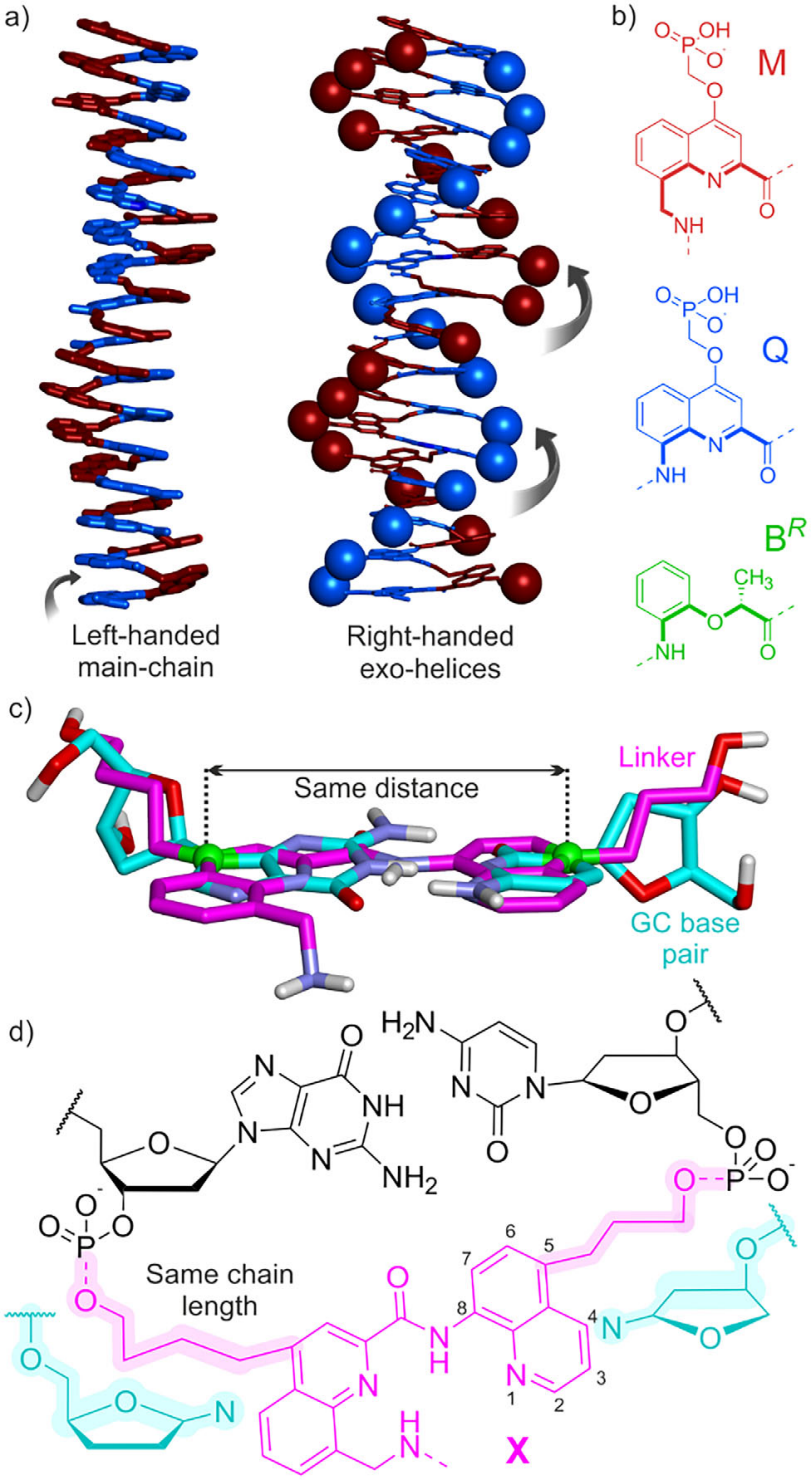
a) Crystal structure of an (MQ)_16_ foldamer.^[^ ^1^ ^]^ Diethyl ester‐protected phosphonic acid side chains have been omitted in the structure at left. The phosphorus atom of the side chain is shown in space‐filling representation at right. Atoms are colored in blue and red for Q and M units, respectively. b) Amino acid building blocks of DNA mimic foldamers. The thick bonds belong to the inner rim of the helix. c) Overlay of an MQ dimer (magenta) and a GC base pair (cyan). Relevant overlapping atoms are displayed as green balls (quinoline Q4 carbon and guanine N9 atoms, quinoline Q5 carbon and cytosine N1 atoms). d) Linker X (magenta) embedded as a turn‐unit for a DNA duplex. The highlighted atoms of the side chains of X match in number with the highlighted atoms of the oxolane rings shown in cyan below.

In (MQ)_n_ sequences, an MQ dimer brings a structural contribution equivalent to that of a base pair (bp) in a B‐DNA duplex.^[^ ^1^ ^]^ It raises the single helix by ca. 3.5 Å –, i.e., the thickness of an aromatic ring – and spans ca. 0.9 helix turn (equivalent to a twist of –0.1 turn), resulting in a twist angle of 36° with respect to the next MQ dimer in the sequence, similar to the twist angle between bps in B‐DNA. An overlay of an MQ dimer and a dG‐dC Watson–Crick–Franklin bp (Figure 1c) highlights their comparable sizes. Specifically, the distance between the nitrogen atoms bonded to the deoxyribose of dA and dT matches with the distance between the carbon atoms in position 4 of M and in position 5 of Q.

This match inspired the design of foldamer‐DNA linker X shown in magenta in Figure 1d. X is an MQ analogue that bears the benzylic amine of M and lacks the carboxylic acid of Q. As such, it can be placed at the C terminus of an (MQ)_n_ helix. Furthermore, X possesses two hydroxy‐terminated side chains so that it can be inserted in a DNA sequence via phosphodiester linkages and serve as a hairpin turn within a unimolecular duplex. Thus, the side chain OH groups of the first and second quinoline rings of X are equivalent to deoxyribose 5′‐ and 3′‐OH groups, respectively. The side chains are in position 4 and 5 of the first and second quinoline rings of X, respectively, so as to place X where a DNA bp would be located. The length of the side chains – four and three methylene units – were designed to match the number of atoms involved if the connecting units had been deoxyribose (Figure 1d). The torsional flexibility inherent to simple alkyl side chains was intended to give some conformational freedom to the hairpin structure. Notably, the overlap mentioned above between the nitrogen atoms of the bases and the C4/C5 of the quinoline rings does not hold for the atoms immediately adjacent – the ribose C1’ carbon atoms and the first exocyclic atoms of the side chains – because of different bond orientations (Figure 1c), and this deviation must be accommodated. Besides, making the structure too rigid from the start may increase chances of failure. Nevertheless, a possible drawback of the flexibility of the side chains of X may be that it allows for two distinct orientations in which either prochiral face of X may stack with the adjacent DNA bp. Molecular models with both orientations suggest that the aromatic overlap between the stacked bp and quinoline rings is larger in the desired orientation (Figure S1). This matters because the other orientation would be conducive of a foldamer helix handedness opposite to that required when (MQ)_n_ is linked to an X‐DNA hairpin (see below).

Figure 2 depicts the synthesis of **1**, a precursor of X suitably protected and activated for oligodeoxyribonucleotide (ODN) solid phase synthesis (SPS). The Fmoc group protects the amine during ODN SPS and may be orthogonally removed afterwards for the subsequent conjugation of a pre‐synthesized (MQ)_n_ segment. Compound **1** was prepared from **6**, the 8‐cyano‐4‐(1*H*)‐quinolone precursor of M,^[^ ^1^ ^]^ and from commercially available 5–bromoquinoline **2**. Both side‐chains were installed on bromo‐quinoline derivatives **3** and **7** via Sonogashira cross‐coupling reactions. The alkyne of **4** was then hydrogenated along with the 8‐nitro group, whereas the alkyne and nitrile of **8** were hydrogenated in separate steps. To differentiate the two OH groups, one DMT protecting group was installed before amide coupling of the two quinoline precursors. Acylation of **5** by **11** occurred selectively on the 8‐amino group to yield **12**, which was activated to give SPS‐ready linker **1**. SPS was performed with cyanoethyl phosphoramidite activation using standard building blocks for d‐ODNs and with protecting groups allowing UltraMild deprotection for L‐ODNs. A series of ODNs was prepared having up to twenty residues and incorporating one or two linker units to form one or two hairpin turns (**13**–**21** in Figure 3a). A typical scale was 4 µmol, eventually performed four times in parallel. Final yields after purification using high‐performance liquid chromatography (HPLC) ranged from 6% to 14% (Table S1). A representative example is highlighted in Figure S2.

**Figure 2:**
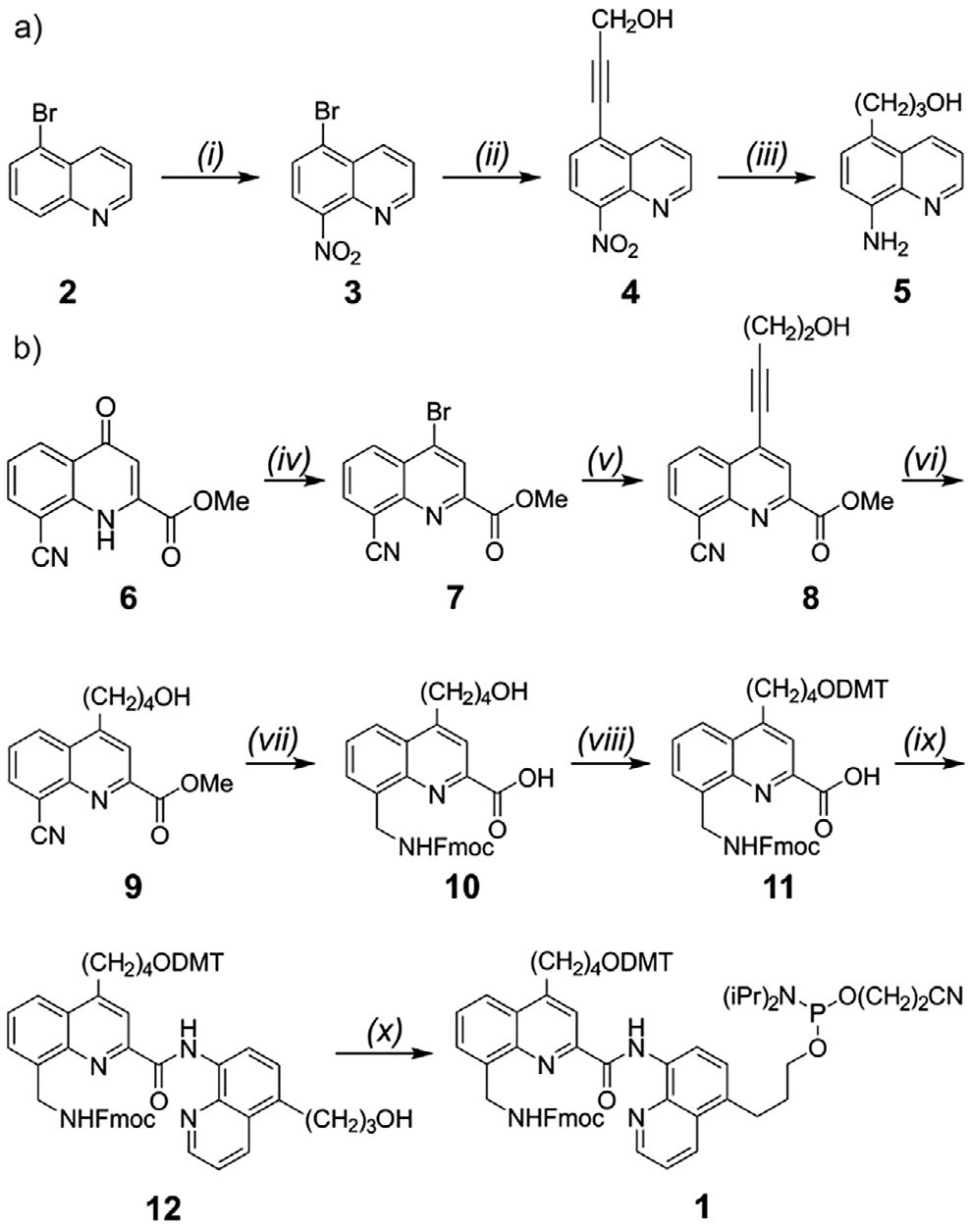
a) Synthesis of precursor **5**. i) KNO_3_, H_2_SO_4_, 0 °C to rt, 18 h, 85%; ii) propargyl alcohol, CuI, XPhos, Pd(dppf)Cl_2_, TEA/dioxane, 85 °C, 3 h, 85%; iii) NH_4_CO_2_H, H_2_, Pd/C, Pd(OH)_2_/C, MeOH, rt, 20 h, 53%. b) Synthesis of DMT‐protected phosphoramidite linker **1** for oligonucleotide synthesis. iv) POBr_3_, DMF, 80 °C, 2 h, 97%; v) 3‐butynol, CuI, XPhos, Pd(dppf)Cl_2_, TEA/dioxane, 85 °C, 3 h, 75%; vi) H_2_, Pd/C, THF, rt, 2 h, 97%; vii) 1. LiOH, THF/H_2_O, 30 min, rt, 2. H_2_, Pd(OH)_2_/C, NaH_2_PO_4_, NH_4_OH, THF/H_2_O, 2 d, rt; 3. Fmoc‐OSu, NaHCO_3_, THF/H_2_O, rt, 4 h, 38% over three steps; viii) DMTCl, pyridine, rt, 2.5 h, 70%; ix) **5**, PyBOP, DIPEA, CHCl_3_, 0 °C, 4 h, 91%; x) CEDCl, DIPEA, CH_2_Cl_2_, 0 °C to rt, 2 h, 80%. Abbreviations: triethylamine (TEA), 1,1′–bis(diphenylphosphino)ferrocene (dppf), dimethoxytrityl (DMT), diisopropylethylamine (DIPEA), fluorenylmethyloxycarbonyl (Fmoc), succinimidyl (Su), benzotriazol‐1‐yl‐oxytripyrrolidinophosphonium hexafluorophosphate (PyBOP), 2‐cyanoethyl *N,N*‐diisopropyl‐chlorophosphoramidite (CEDCl).

**Figure 3:**
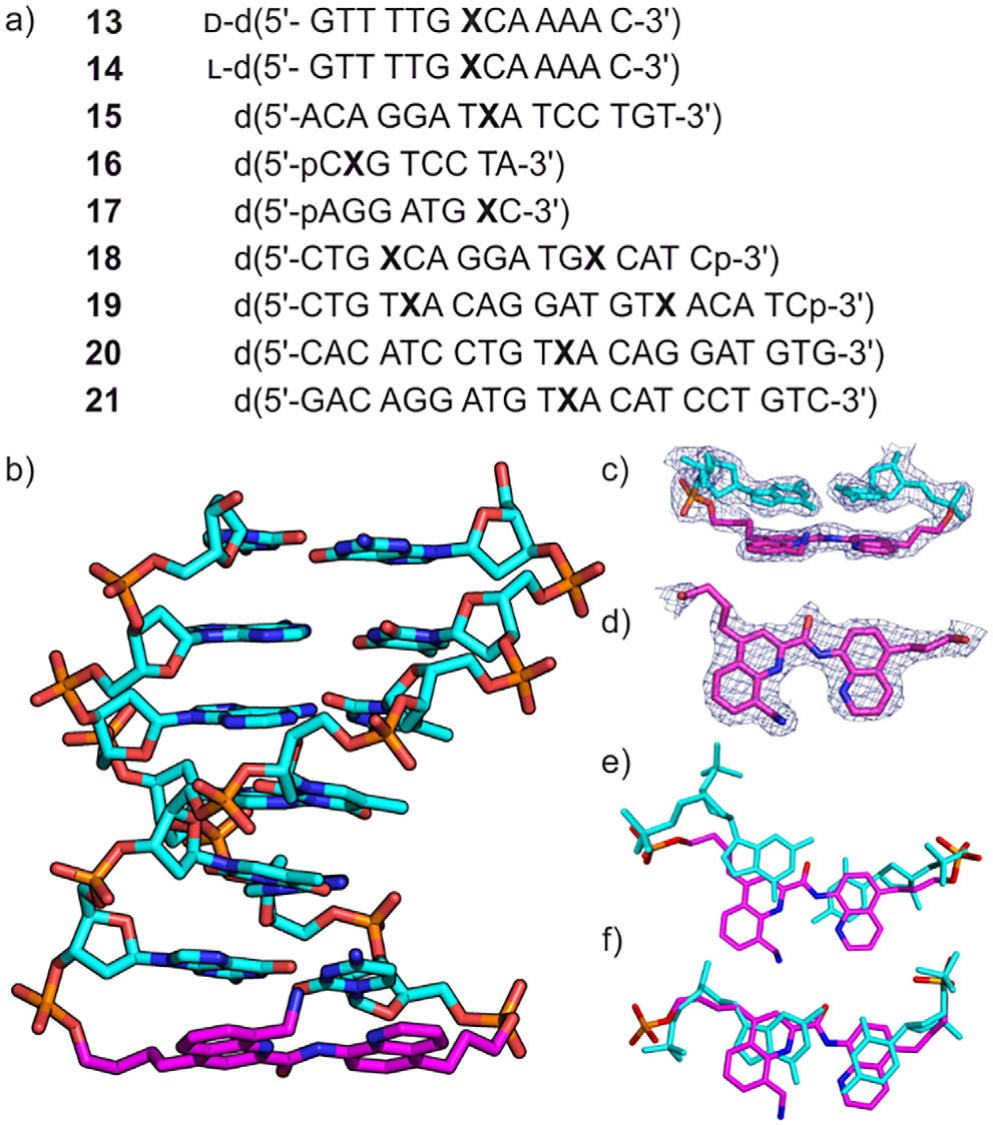
a) Synthesized oligonucleotides with the linker X highlighted in bold. All strands were synthesized from d‐deoxynucleoside phosphoramidites except **14**, the enantiomer of **13**, which was made using l‐deoxynucleosides. Sequences are terminated by free hydroxy groups at 3′ and 5′ ends except where a phosphate (p) is indicated (in **16**‐**19**). b)–f) Solid state structure of **13**. One (hairpin 1) out of the two molecules present in the asymmetric unit is shown in b) with the linker carbon atoms colored in magenta and other carbon atoms and nitrogen, oxygen and phosphorus atoms colored in cyan, blue, red and orange respectively. The final Fourier 2F_o _− F_c_ electron density at 2.5 Å resolution and contoured at 1 σ are shown around the linker and adjacent GC bp of hairpin 1 c) and around the linker in top view (d). The overlay of the linker (carbon atoms shown in purple) and a base pair (in cyan) where it would be expected upon extending the DNA duplex in hairpin 1 e) and hairpin 2 f). See Figure S9 for details.

Sequences **13**–**15**, **20**, and **21**, were all expected to fold as single hairpins due to their complementary DNA segments. The latter two were used to assess binding of transcription factors that recognize the GGA motif (see below). Self‐complementary sequences **18** and **19** have two hairpins and could in principle be macrocyclized by a single ligation step. Sequences **16** and **17** are complementary to each other and their duplex could in principle be macrocyclized through two ligation steps. However, efforts to implement ligation within the DNA hairpins were not pursued at this stage. Sequence **13**, where X links complementary d(GT_4_G) and d(CA_4_C) segments was specifically prepared for structural investigations because related sequences with a stilbenediether linker had been shown to form particularly stable hairpins and to produce crystals suitable for X‐ray diffraction (XRD) analysis.^[^ ^23^, ^24^ ^]^ The melting temperature of **13** was measured at 78 °C, while the d(GT_5_G)•d(CA_5_C) duplex is not expected to be stable at room temperature, indicating a considerable stabilization (Figure S3). Using the sitting drop vapor diffusion method, crystals of **13** were obtained under multiple crystallization conditions (Figure S4). However, single crystal XRD only showed a weak DNA fiber‐like diffraction pattern (Figure S5). Encouraged by our previous success in crystallizing diverse DNA sequences using racemic DNA mixtures,^[^ ^25^, ^26^, ^27^ ^]^ we prepared **14**, the lll enantiomer of **13**. Crystals of the **13**+**14** racemate diffracting to a resolution of 2.5 Å were obtained readily and the structure could be solved in centrosymmetric space group *P*‐1 using an earlier structure^[^ ^23^, ^24^ ^]^ as molecular replacement model (Figures 3b‐d, S6‐S10 and Table S2). The asymmetric unit consisted of two independent molecules and four Mg^2+^ ions. Both molecules adopted a hairpin B‐form DNA conformation albeit with some variations of sugar puckering (Tables S3‐S7) However, the structure of the two hairpins differed significantly for both the DNA components and the X linker (Figures 3 and S8). Highlighting these differences, the root mean square deviation of the two hairpins superimposed based on calculations performed for all DNA atoms was 1.27 Å.^[^ ^28^ ^]^ Importantly, in both molecules, the quinoline rings of X linkers are coplanar and stacked to the adjacent dG−dC bp in the predicted preferred orientation (Figure S1). The X units have different twist angles with respect to the dG−dC bp but in both cases, this twist extends the twist between DNA bps (Figure 3e,f and S10). X is thus equivalent to an additional bp. To assess the coherence of the position of X units with respect to where a base pair would be instead, each hairpin molecule was overlaid with a copy of itself frame‐shifted by one bp along the helix axis (Figures 3e,f and S10). X units were thus made to overlap with adjacent dG–dC bp. For one hairpin, the match was excellent (Figure 3f) whereas for the other the X unit was in part twisted away from an ideal bp position (Figure 3e), reflecting the flexibility of the side chains of X. Altogether the solid‐state hairpin structures validate the initial design.

We next explored the conjugation of (MQ)_n_ segments to the benzylic amine of X after its installation within an ODN. Conjugation was first attempted on unpurified ODNs still attached to the SPS glass beads with their protecting groups still on, except for the Fmoc group of X. Using unprotected Ac‐(MQ)_4_‐OH **22**, that is, with free phosphonic acid side chains, the terminal carboxylic acid function was successfully activated with EDC (1‐ethyl‐3‐(3‐dimethylaminopropyl) carbo‐diimide hydrochloride) but no conjugation product could be detected. This route was not pursued but deserves to be revisited because fragment condensation of foldamers on peptides or other foldamers on solid phase is reliable.^[^ ^29^, ^30^ ^]^ Conjugation was successful when performed in solution using purified deprotected ODNs containing X. However, mass spectrometry revealed the presence of dehydrated impurities.^[^ ^31^ ^]^ These side reactions could be suppressed by using DMTMM (4‐(4,6‐dimethoxy‐1,3,5‐triazin‐2‐yl)‐4‐methyl‐morpholine hydrochloride) instead of EDC as coupling reagent. These milder conditions required excess foldamer (4.6 equiv.). Final purification was achieved by HPLC using an NH_4_OAc buffer system, leading to good separation of the ODN‐foldamer conjugates from excess foldamer. Conjugates **24** and **25** (Figure 4a) were prepared and purified on a 50–150 nmol scale. The melting temperature of **24** was measured at 87 °C (Figure S3), an increase of 9 °C compared to **13**, suggesting that the linker X is better held in place when linked to a foldamer. Note it has been shown previously that (MQ)_n_ oligomers do not melt.^[^ ^8^ ^]^

**Figure 4:**
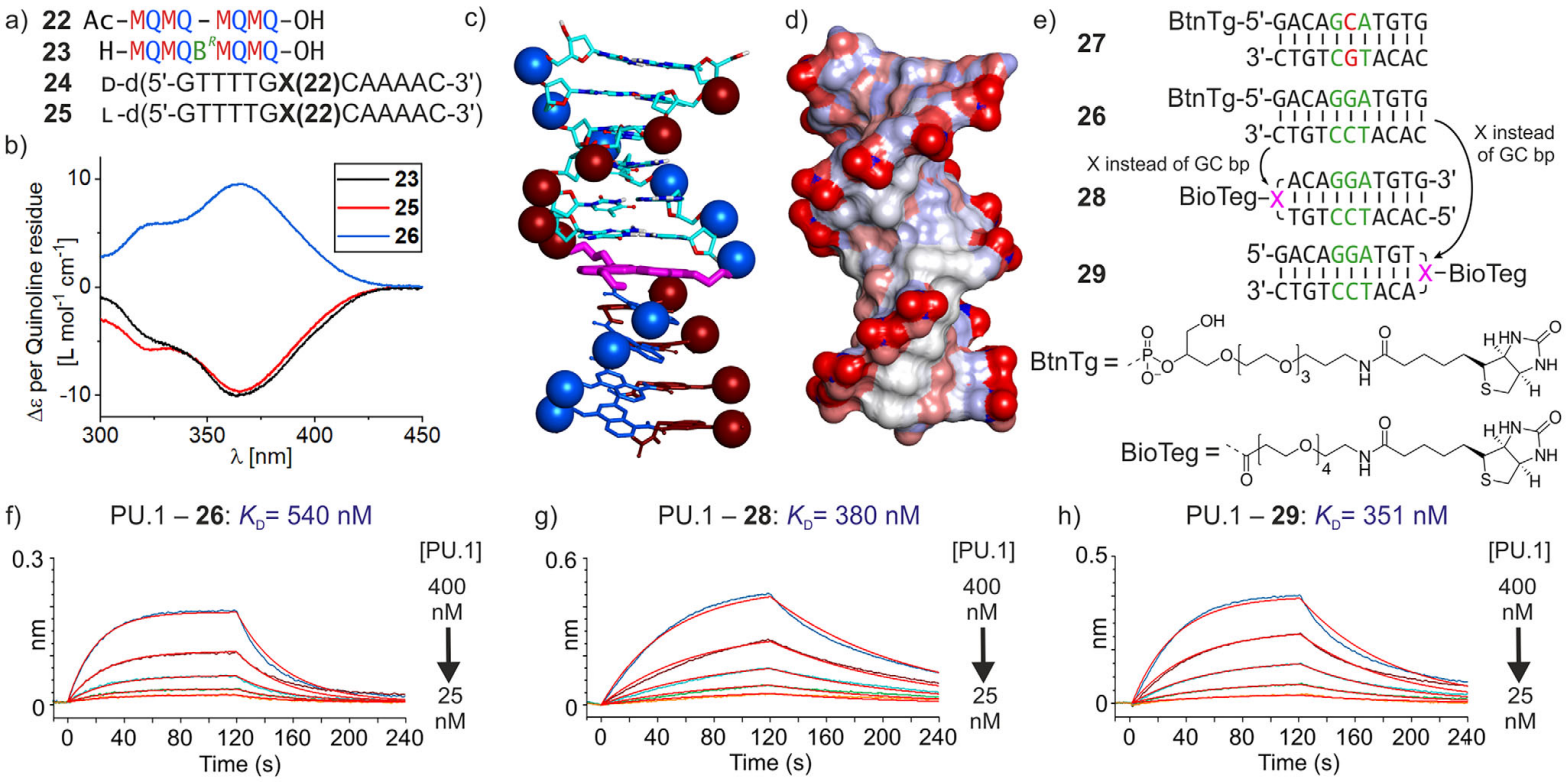
a) Sequences of synthesized foldamers and foldamer‐DNA conjugates. **X(22)** indicates an X unit, with its amine acylated with the acid of foldamer **22**. b) CD spectra of conjugates **24** and **25** in comparison with reference foldamer **23**. Spectra have been normalized per quinoline residues. Sample solutions were typically 60 or 80 µM in 2 mm cuvettes. c) Energy‐minimized model of foldamer‐DNA conjugate **24** with DNA atoms colored in cyan, red, blue and white for carbon, oxygen, nitrogen and hydrogen atoms respectively. Linker atoms are colored in magenta. Foldamer atoms are displayed in blue and red for Q and M respectively. The two phosphorus atoms are shown in space filling representation. DNA phosphorus atoms are colored according to the foldamer exohelix that they extend. d) Solvent accessible surface for **24** colored according to atom charge. e) Biotinylated DNA duplexes with (**26**) and without (**27**) GGA consensus sequence and biotinylated hairpins **28** and **29** containing X. BLI sensorgrams of the binding of PU.1 to immobilized **26** f), **28** g), and **29** h).

Circular dichroism (CD) spectroscopy was used to assess the conformation of the chimeric molecules in solution. (MQ)_n_ foldamers are achiral and exist as racemic mixtures of right‐handed (*P*) and left‐handed (*M*) helical conformers. The helix handedness can be biased quantitatively toward the *M* helical conformer upon insertion of a single chiral B*^R^* unit (Figure 1b) in a sequence.^[^ ^2^, ^32^ ^]^ For example, the ^1^H nuclear magnetic resonance spectrum of **23** shows a single set of sharp resonances indicating the presence of a single diastereomeric conformer, and its CD spectrum shows a negative band as a result of its *M* helicity, with a maximum at 365 nm, a wavelength at which DNA does not absorb (Figures 4b and S11). Importantly, it is the *M* helical conformation of (MQ)_n_ that mimics the shape and charge distribution of B‐DNA because it projects its phosphonate side chains so that they form two *P* exo‐helices that match with the *P* double helix of DNA (Figure 1a).^[^ ^33^, ^34^ ^]^ Within a DNA‐foldamer chimera, the orientation of the X unit with respect to the DNA duplex dictates the handedness of the foldamer. If X is oriented as in the original design and in the solid state structure of **13**, the foldamer helix must adopt the desired *M* handedness whereas a 180° flip of X would induce *P* helicity (Figure S12).^[^ ^35^, ^36^ ^]^ Satisfactorily, the CD spectrum of **24**, which possesses a foldamer segment identical to **23** but lacking the B*^R^* unit, shows a negative band at 365 nm indicating it is left‐handed. The intensity of the band and its overlap with the CD spectrum of **23** indicate that foldamer helix handedness bias in **24** is essentially quantitative and that its shape is not altered from its conjugation to the ODN (changes in helix shape are typically reflected in the CD spectrum). As expected, the CD spectrum of **25**, the l enantiomer of **24**, shows a band with opposite sign (Figure 4b). Our attempts to crystallize enantiopure **24** or the **24 **+ **25** racemic mixture did not yield diffracting crystals. Nevertheless, the solution data clearly establish that (MQ)_n_ segments connected to a DNA duplex via an X linker adopt the desired *M* helicity, that is, DNA chirality is transferred to the foldamer helix. The energy‐minimized structure shown in Figure 4c, with the arrays of anions and the grooves of the DNA and foldamer segment in register, represents a plausible model of the conformation of **24**.

Finally, we used bio‐layer interferometry (BLI) to assess the binding of two transcription factors, PU.1^[^ ^37^ ^]^ and SAP1,^[^ ^38^ ^]^ to biotinylated hairpin ODNs containing X and immobilized on streptavidin biosensors (Figures 4d‐h, S13–S16, and Table 1). Both proteins bind selectively to DNA sequences that include a d(GGA) motif. We first confirmed binding to **26**, a biotinylated 11‐bp DNA duplex including GGA. We also verified the drop in binding affinity when GGA was replaced by GCA using duplex **27**. We then measured binding of PU.1 and SAP1 to sequences **28** and **29**, the biotinylated analogues of **20** and **21**, respectively which correspond to duplex **26** where either of the terminal bp has been replaced by a biotinylated X. *K*_D_ values were in all cases similar to those measured with the original duplex DNA (Table 1), suggesting that the X unit does not perturb the DNA structure and does not hamper protein binding in these two cases. We nevertheless noted that both association and dissociation were faster with hairpin duplexes **28** and **29** than with **26** (Table S8).

**Table 1:** *K_D_* values of biotinylated DNA duplexes and X‐containing hairpins against transcription factors PU.1 and SAP1.

*K*_D_ (nM)^a)^	26^b)^	27^b)^	28^b)^	29^b)^
PU.1	330 ± 9	1600 ± 50	380 ± 0.5	351 ± 0.4
SAP1	1080 ± 6	^c)^	1100 ± 130	880 ± 138

^a)^ Calculated using a 1:1 model and global curve fitting from BLI sensorgrams measured in 25 mM Na_2_HPO_4_ (pH 7.5) containing 250 mM NaCl, 1 mM EDTA, 0.05% Tween 20.

^b)^ Biotinylated ligands were immobilized on streptavidin sensors and protein analytes were in solution.

^c)^ No binding detected.

The purpose of this work was to create chimeric DNA‐foldamer molecules with a structurally consistent arrangement of the DNA and foldamer subcomponents despite their different chemical nature. We have designed and synthesized an artificial linker based on a diamide quinoline unit that can be incorporated into DNA by standard phosphoramidite synthesis and that promotes a DNA hairpin structure. The linker can be elongated by a DNA mimic foldamer that extends the grooves and double helical array of negative charges of DNA. The *M* handedness of the helical foldamer is then controlled by the d stereochemistry of the DNA. The next step will be to explore how DNA‐foldamer hybrids can be exploited as a new generation of DNA decoys^[^ ^39^, ^40^ ^]^ to target sequence‐selective DNA–binding proteins. While the foldamer component has been shown to enhance affinity for DNA‐binding proteins,^[^ ^1^, ^5^, ^6^, ^7^, ^8^ ^]^ the DNA component may help promote selectivity for defined protein targets by including a consensus sequence, in particular for transcription factors many of which have been considered undruggable.

## Supporting Information

The authors have cited additional references within the Supporting Information.^[^ ^41^, ^42^, ^43^, ^44^, ^45^, ^46^, ^47^, ^48^, ^49^, ^50^, ^51^, ^52^, ^53^, ^54^, ^55^, ^56^, ^57^, ^58^, ^59^, ^60^, ^61^, ^62^, ^63^, ^64^ ^]^

## Conflict of Interests

The authors declare no conflict of interest.

## Supporting information



Supporting Information

## Data Availability

The data that support the findings of this study are available in the Supporting Information of this article.
