# Supplementary material for: Interfacing B‐DNA and DNA Mimic Foldamers
Source: Angew Chem Int Ed Engl. 2025 Jun 17;64(31):e202505273. doi: 10.1002/anie.202505273 (PMC12304850; doi:10.1002/anie.202505273)
Supplement: Supplementary file 1 — Supporting Information [file ANIE-64-e202505273-s001.pdf]

Table of contents

1 Supplementary figures and tables 3

1.1 Supplementary figures 3

1.2 Supplementary tables 15

2 Materials and Methods 19

2.1.1 General methods for synthesis and analysis for small molecule intermediates, foldamers and conjugates 19

2.1.2 General methods for nucleic acid synthesis and purification 20

2.1.3 Crystallography 21

2.1.4 Molecular modeling 22

2.1.5 Recombinant protein expression and purification 23

2.1.6 Bio-layer interferometry 26

3 Synthetic procedures 27

3.1 Linker synthesis 27

3.1.1 Synthesis of 3’-terminal quinoline ring 27

3.1.2 Synthesis of 5’-terminal quinoline ring 29

3.1.3 Linker assembly & activation 33

3.2 Foldamer synthesis 35

3.2.1 General solid-phase foldamer synthesis 35

3.2.2 Removal of the diethyl-phosphonate protecting groups 36

3.2.3 Cation exchange chromatography 37

3.2.4 Synthesized foldamers 37

3.3 Conjugate synthesis and oligonucleotides 38

3.3.1 DNA hairpin biotinylation 38

3.3.2 By EDC hydrochloride 38

3.3.3 By DMTMM hydrochloride 38

4 Spectra & Chromatogramms 39

4.1 NMR spectra 39

4.2 HPLC chromatograms 49

4.2.1 Foldamer chromatograms 49

4.2.2 Oligonucleotide chromatograms 50

4.2.3 Conjugate chromatograms 52

4.3 Mass spectra 53

4.3.1 Foldamer mass spectra 53

4.3.2 Oligonucleotide mass spectra 54

4.3.3 Conjugate mass spectra 56

5 References 58

1 Supplementary figures and tables

1.1 Supplementary figures

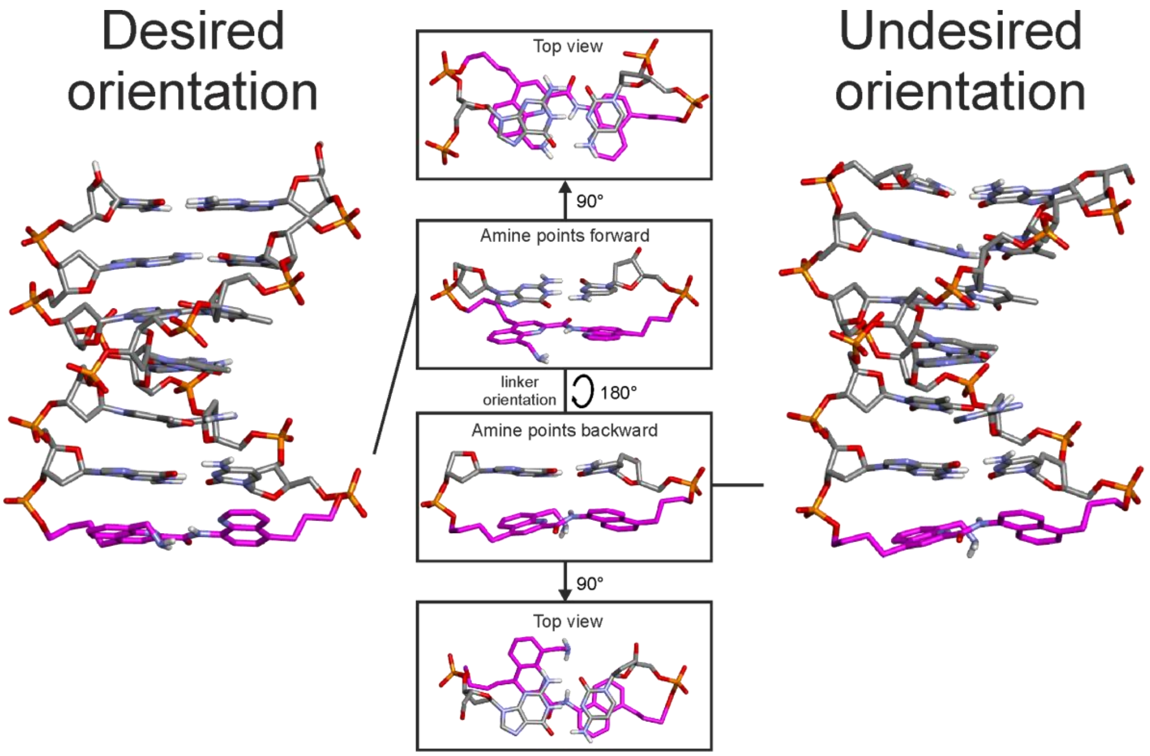

Figure S1. Energy-minimized molecular models of the two orientations that are possible for the diquinoline linker X within a DNA hairpin. The orientation depicted with the amine pointing forward (left) is the preferred orientation for a continuation of the DNA double-helical array of negative charges when a foldamer is appended to the turn unit. Atoms are colored in grey, red, orange and white for carbon, nitrogen, oxygen, phosphorus and hydrogen respectively. Non-polar hydrogen atoms are hidden. Carbon atoms of the linker unit are colored in magenta.

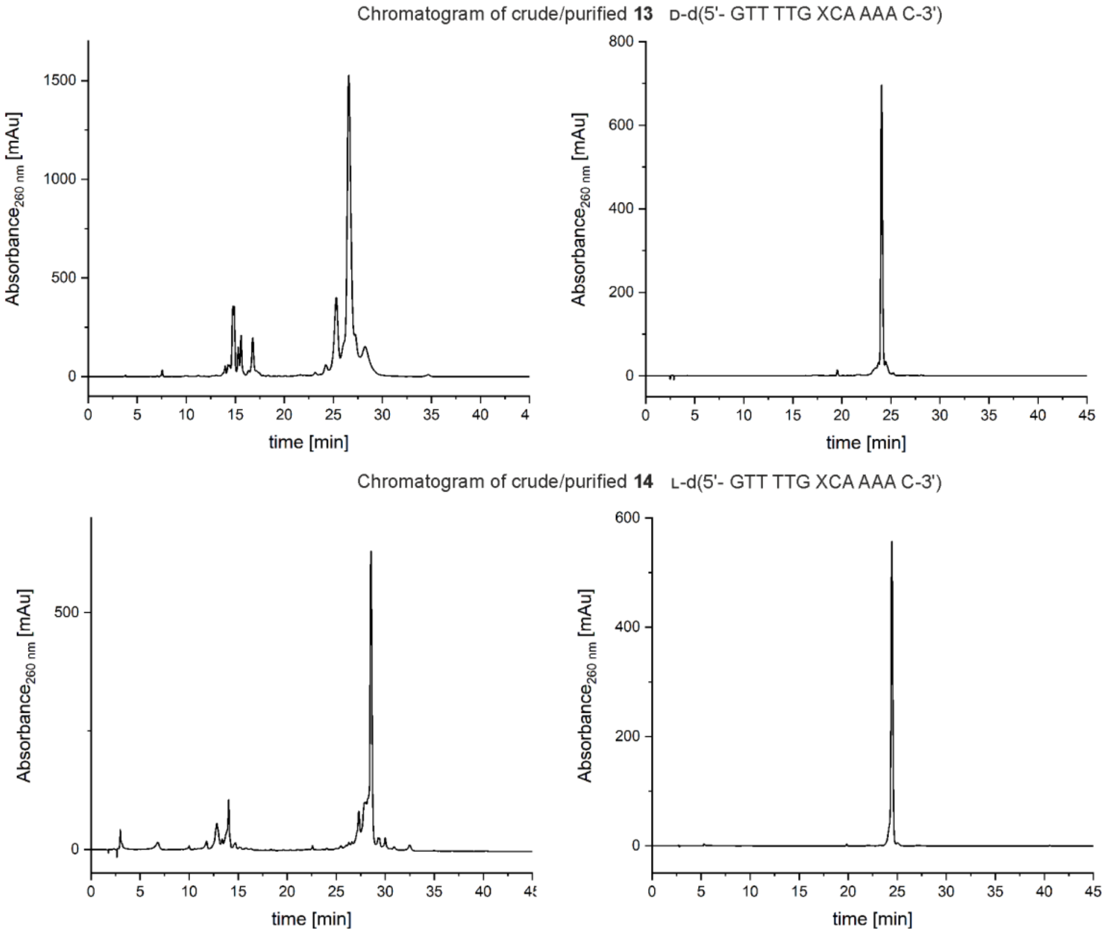

Figure S2. Representive examples of crude and purified chromatograms for ODN synthesis using standard phosphoramidite chemistry (**13**) and ODN synthesis using ultraMild oligonucleotide phosphoramidite chemistry. (**14**).

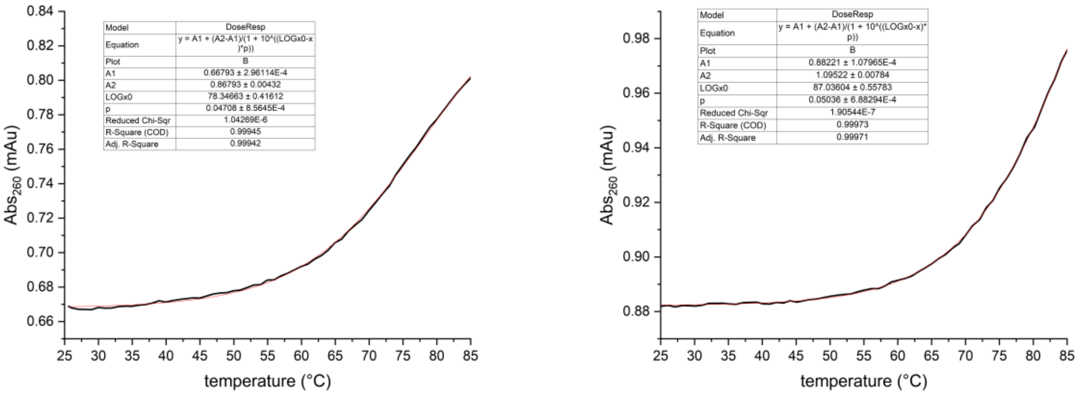

Figure S3. Melting curve of **13** (left) measured in a 1 cm cuvette at a concentration of 3.5 µM in a 1 cm cuvette and **24** (right) at a 25 µM concentration in a 2 mm quartz glass cuvette in sodium phosphate buffer (100 mM, pH = 7.5). Data was fit with a sigmoidal DoseResp function and their melting point was read from their respective inflection points.

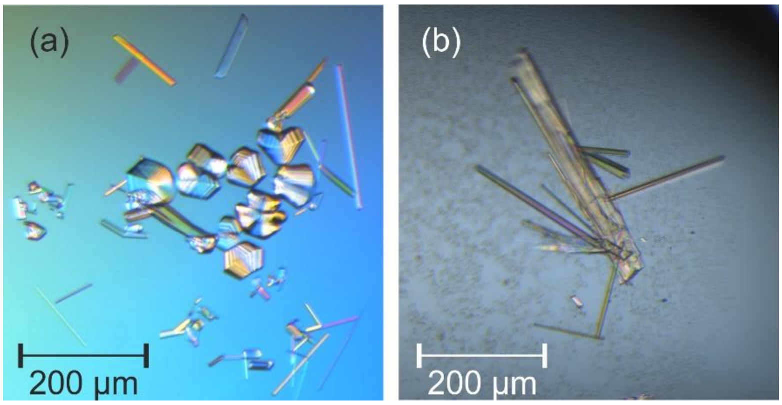

Figure S4. Representative examples of crystals of **13** (D-d(GT$_4$G)−X(H)−d(CA$_4$C)) and racemic mixture **13/14** D/L-d(GT$_4$G)X(H)d(CA$_4$C) observed under crossed polarizing microscope.

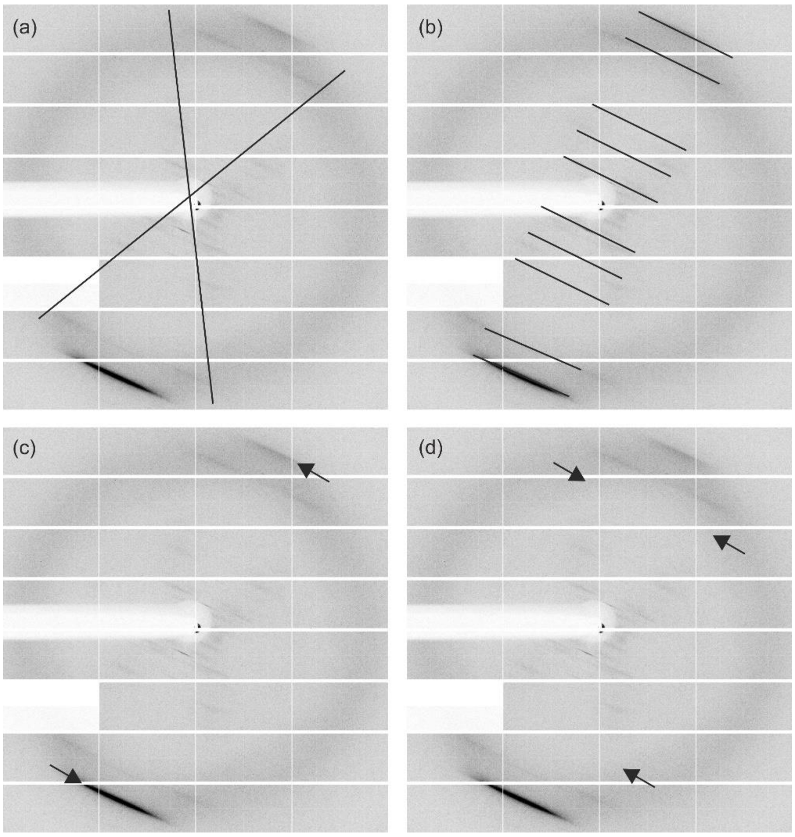

Figure S5. Diffraction of crystals of **13** (D-d(GT$_4$G)−X(H)−d(CA$_4$C)). The weak "X"-form distribution of diffraction peaks (a) indicates a helical structure[41] while the distances between the layer lines (b) imply the period of a helical turn. The broad extended lines at the top and bottom (c) correspond to small periodical features- the base pairs or linker. The absence of broad peaks (d) indicates the modulation of the interference pattern *i.e.* interference between the waves diffracted at two helical structures that are relatively shifted to each other along the long axis, implying a double helical structure. The experiment was carried out as a single crystal XRD experiment (unlike X-ray diffraction experiments using DNA fibers) and a limited amount of structural information was obtained. However, the DNA fiber-like pattern implied that the single crystals indeed corresponded to a hairpin with B-DNA like features and were not belonging to any small-molecule components of the crystallization reagents such as salts, buffer and precipitants that tend to diffract to high resolution and possess smaller unit cells.

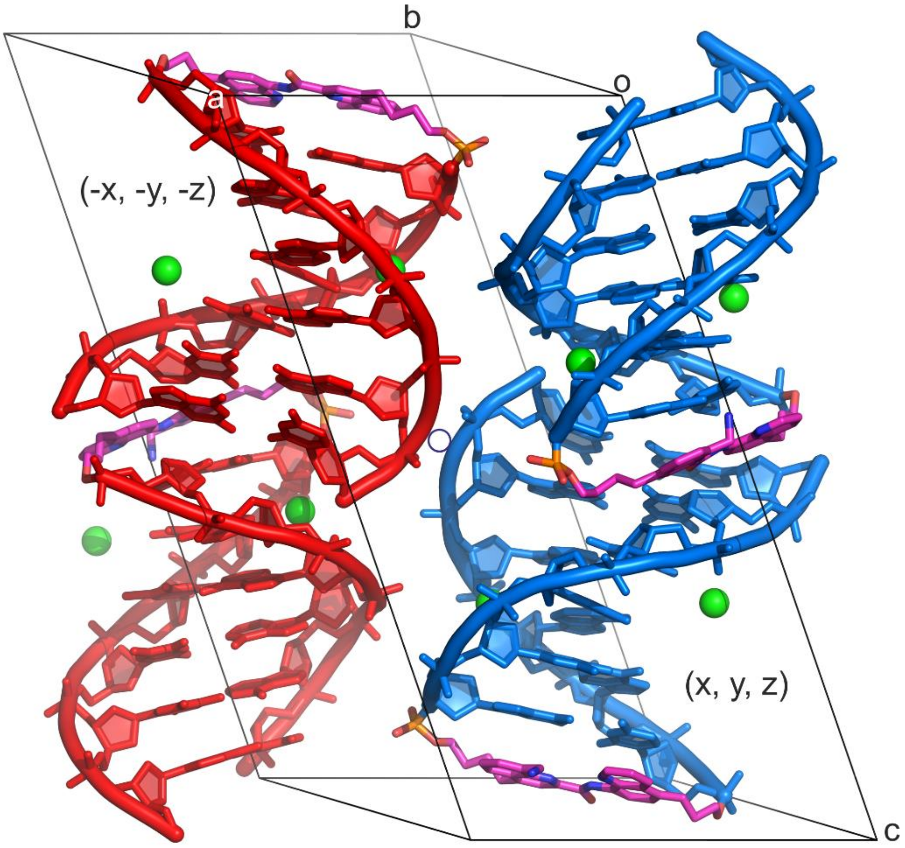

Figure S6. Unit cell arrangement of L/D-d(GT4G)−X−d(CA4C) hairpins **13/14** in P-1 space group. A blue circle represents the point of inversion. The L and D enantiomers are colored in red and blue, respectively. The diquinoline X linker atoms are colored magenta, red, blue and orange for carbon, oxygen, nitrogen and phosphorus respectively. Mg^{2+} ions are shown as green spheres. Bound water molecules have been omitted for clarity. The asymmetric unit consists of two right-handed D hairpins in (x, y, z).

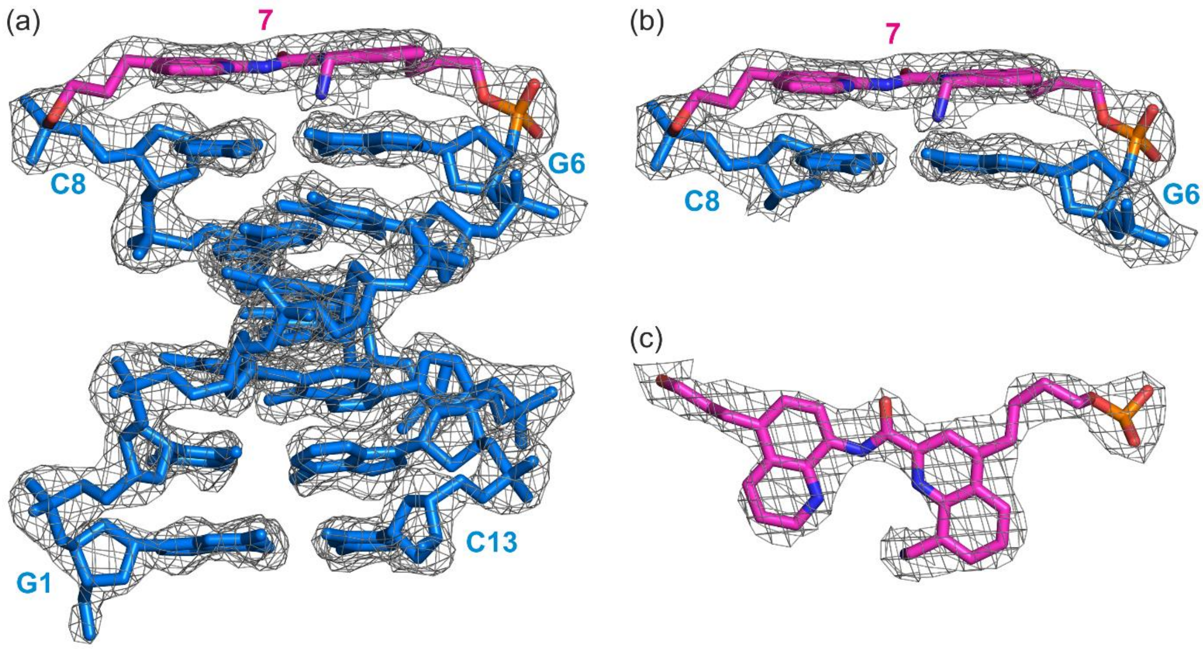

Figure S7. Quality of the structure. The final Fourier 2Fo−Fc electron density at 2.5 Å resolution and contoured at 1 σ around (a) Hairpin 1, (b) its residues G6-X7-C8, and (c) the X linker. DNA residues are colored blue and the linker atoms are colored in magenta, red, blue and orange for carbon, oxygen, nitrogen and phosphorus respectively.

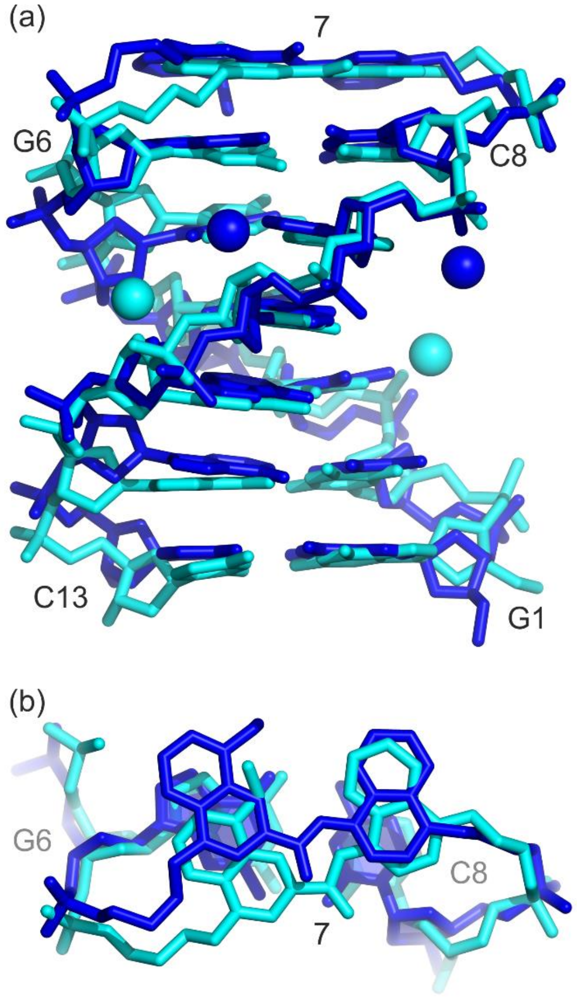

Figure S8. Conformations of D-d(GT_4G)−X−d(CA_4C). a) Overall superposition of Hairpin 1 (blue) and Hairpin 2 (cyan) structures along with the Mg^{2+} ions coordinating their minor groove. b) Geometries and relative orientations of the X linkers in the two hairpins. The view is approximately perpendicular to the adjacent G:C base pair.

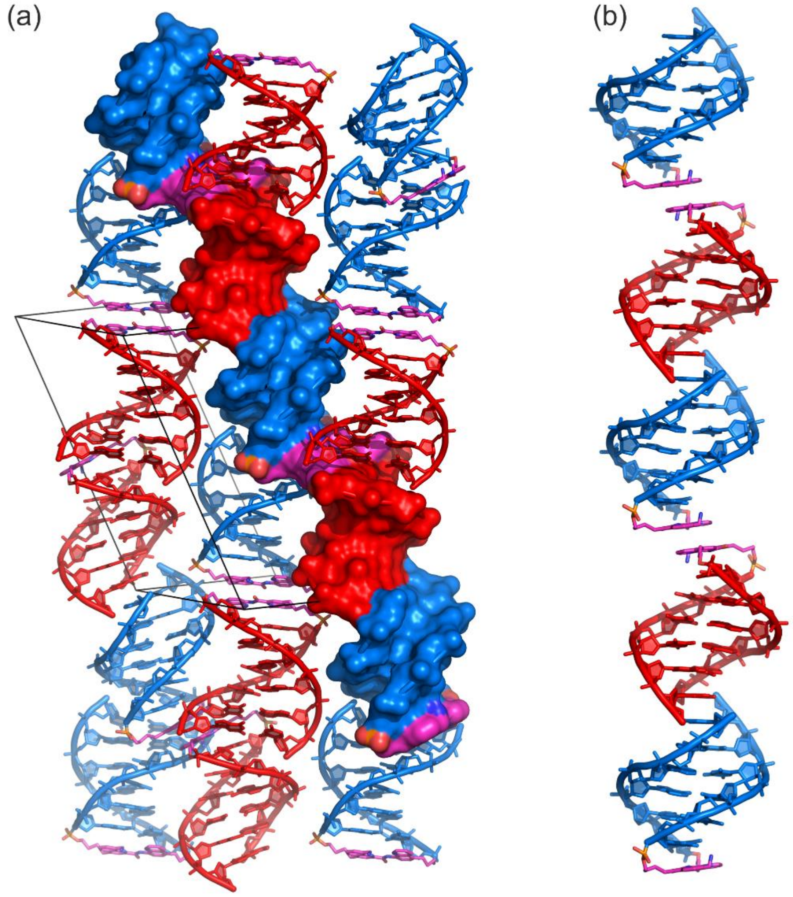

Figure S9. (a) Crystal packing arrangement L/D-d(GT$_4$G)−X−d(CA$_4$C) hairpins **13/14**. The L and D enantiomers are colored in red and blue, respectively. The X linker atoms are colored in magenta, red, blue and orange for carbon, oxygen, nitrogen and phosphorus respectively. Mg^{2+} ions and bound water molecules have been omitted for clarity. A heterochiral pseudo-continuous hairpin stacking arrangement is shown in (a) as a surface and in (b) as stick representations, respectively. The presence of a racemic mixture and of the diquinoline linker X gave rise to a novel packing arrangement of hetero-chiral DNA hairpins stacked into pseudo-continuous via the terminal residues of the DNA stem or the diquinoline linkers.

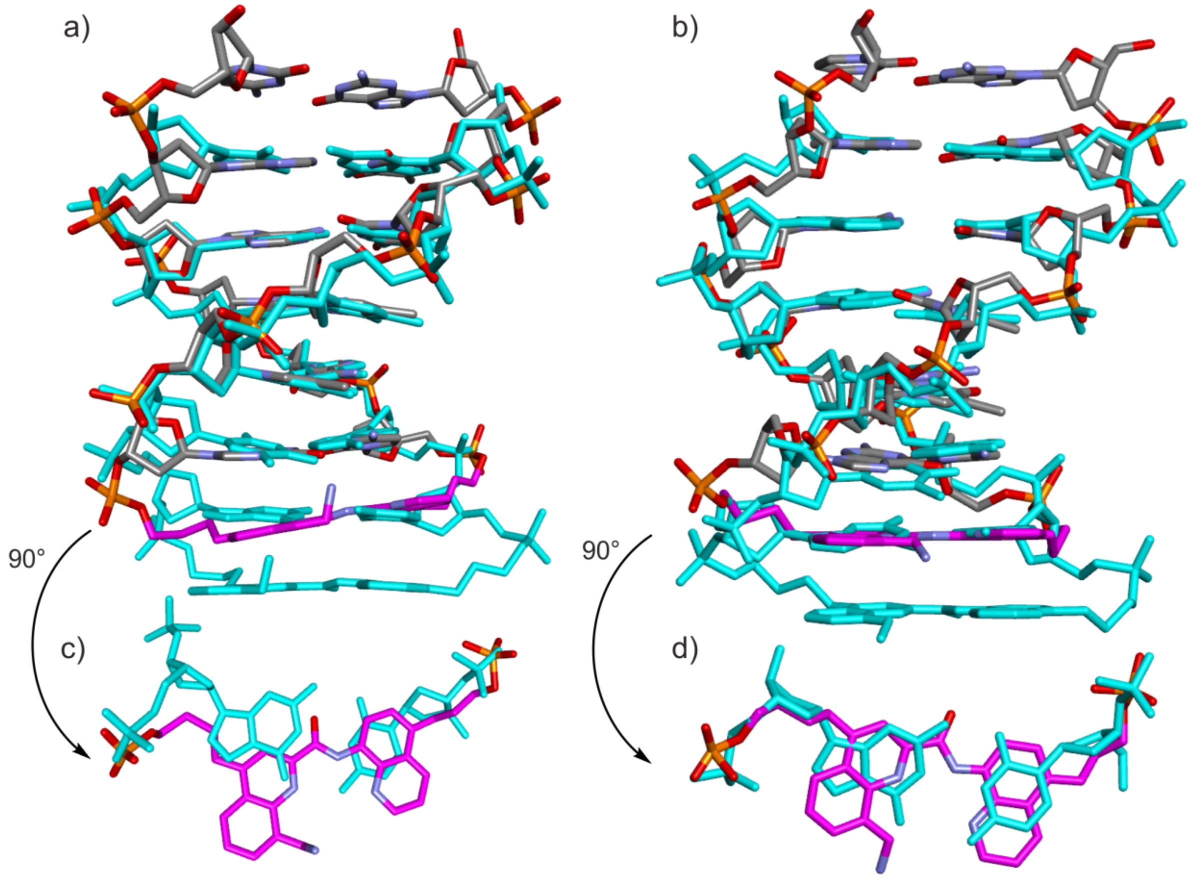

Figure S10. Crystal structure of hairpin 1 (a) and hairpin 2 (b) with linker carbon atoms in colored in magenta and carbon, nitrogen, oxygen and phosphorus colored in grey, blue, red and orange respectively. Additionally, another molecule of the same hairpin, shifted and fitted to match the DNA backbone was overlayed in cyan to highlight the orientation of the linker position in reference to a DNA base pair.

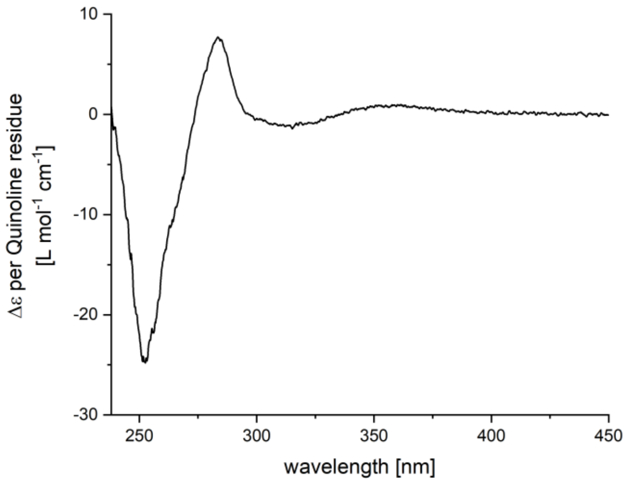

Figure S11. CD spectrum of compound **13** normalized per quinoline residue. Note the weak intensity of the positive band at 350 nm which can be assigned to the diquinoline linker chromophore, stacked on the adjacent base-pair with a right-handed twist as an extension of the B-DNA duplex.

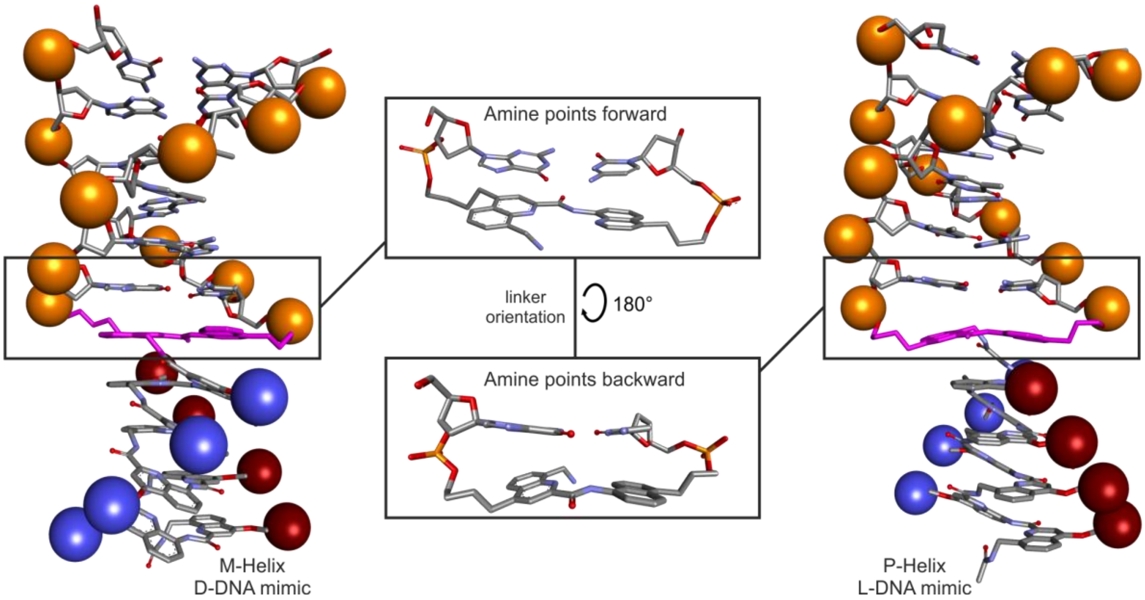

Figure S12. Energy-minimized molecular models of DNA hairpins with an appended foldamer segment in the two possible orientations of the diquinoline hairpin turn X. Only the orientation depicted with the amine pointing forward (left) gives rise to a continuation of the double-helical array of negative charges from the DNA to the foldamer. In the other orientation (right), the arrays of negative charges have opposite handedness in the DNA and foldamer segments. Atoms are color in grey, blue, red, orange and white for carbon, nitrogen, oxygen, phosphorus and hydrogen respectively. Phosphorus atoms are displayed in space-filling representation in respective blue, red and orange for Q units, M units and nucleotides, respectively. Non-polar hydrogen atoms have been omitted for clarity. Carbon atoms of the linker unit X are colored in magenta.

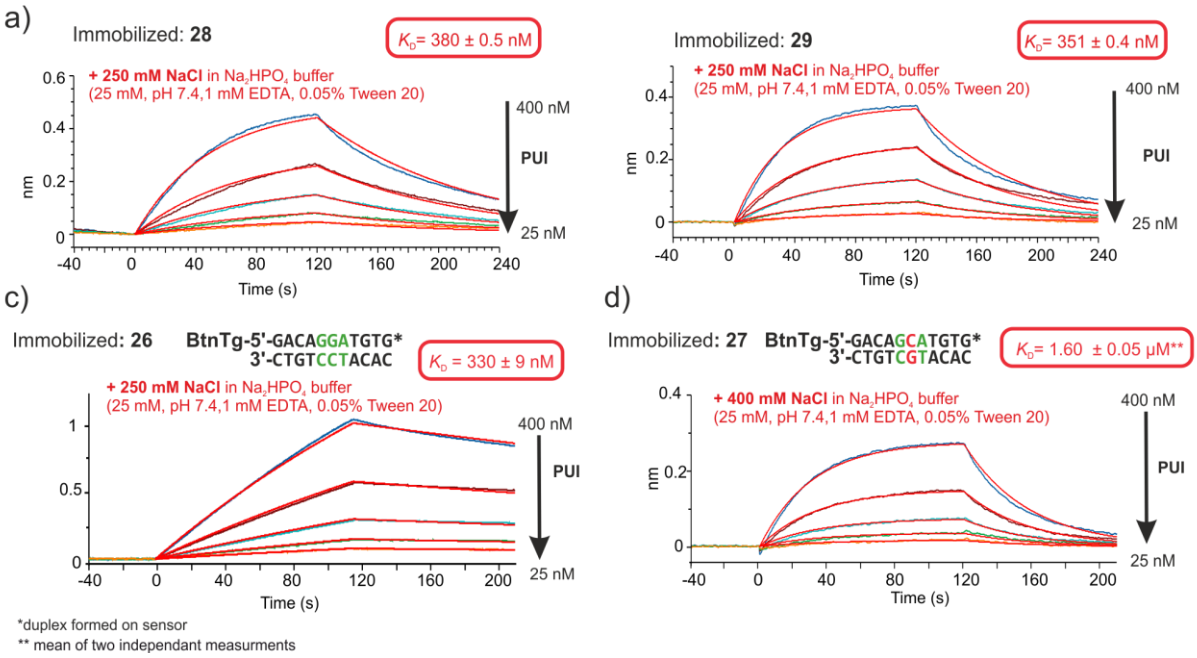

Figure S13. BLI sensorgrams of PU.1 against biotinylated hairpins containing X **28** (a) and **29** (b) with their respective fits and buffer composition. c) Immobilized dsDNA (see Figure S14 and section 2.1.6 for general information) containing the cognate GGA binding site with comparable length and sequence as **28** and **29**. For a-c), the K_D values are in the same high nM range. d) Immobilized dsDNA containing a non-cognate GCA motif measurements against PU.1. The GC to CG mutation in the cognate binding site leads to a drop in K_D value.

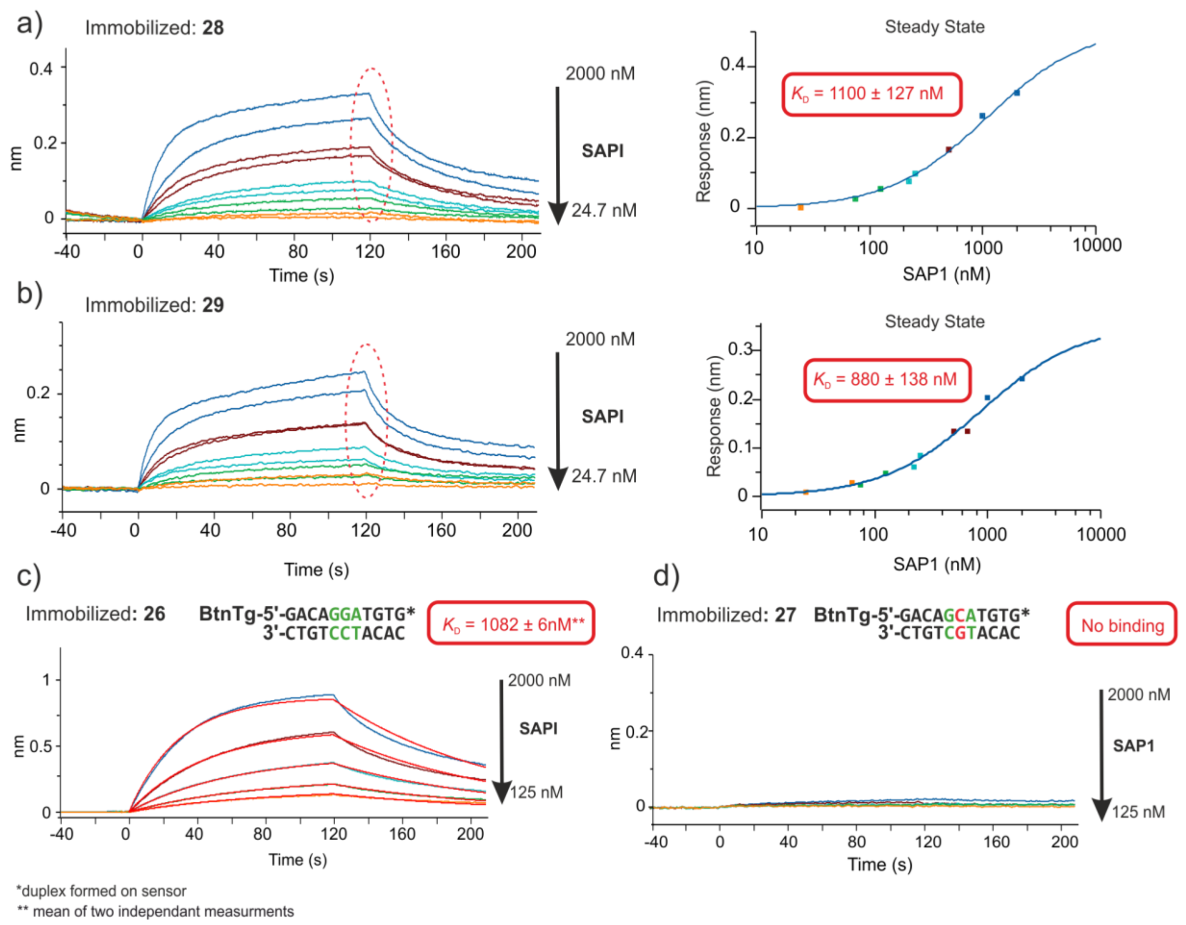

Figure S14. BLI sensorgrams of SAP1 against biotinylated hairpins containing X **28** (a) and **29** (b) with the sensorgrams (left) and a steady-state fit with the values at the end of the association phase at 120 seconds (highlighted by the dashed red ellipse in the sensorgrams). c) Immobilized dsDNA (see Figure S14 and section 2.1.6 for general information) containing the cognate GGA binding site with comparable length and sequence as **28** and **29**. For a-c), the K_D values are in the same low µM to high nM range. d) Immobilized dsDNA containing a non-cognate GCA motif measurements against SAP1. The GC to CG mutation in the cognate binding site leads to a complete loss in binding.

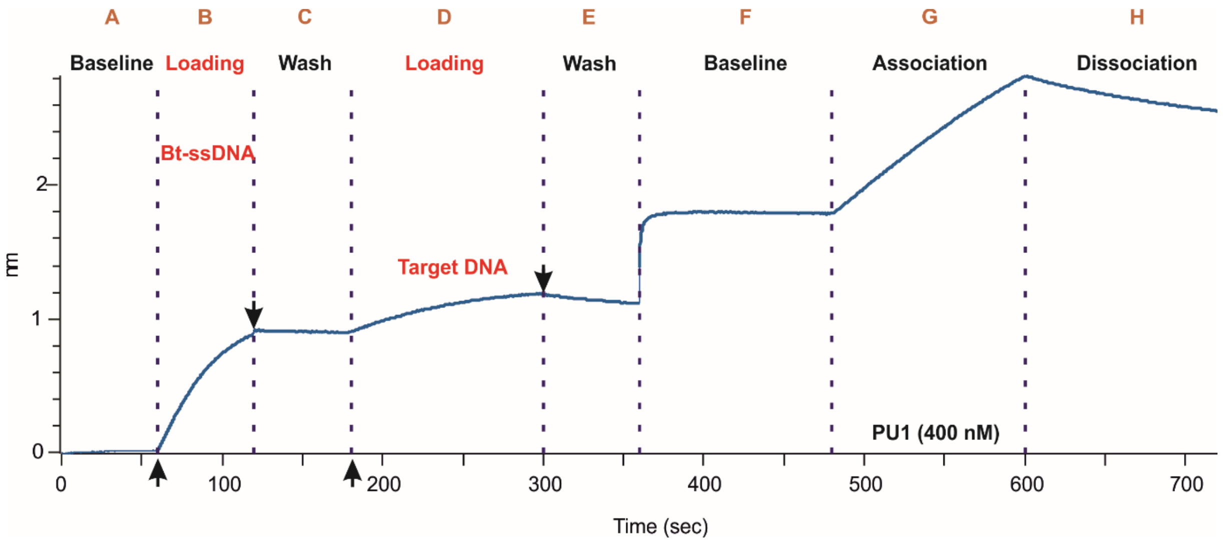

Figure S15. Sensorgrams of ssDNA immobilization, target DNA hybridization (**26**) on biosensors and kinetic curves of association and dissociation exemplified at the highest PU1 concentration (400 nM). The height independent steps (*A*-*H*) are shown, starting from a baseline in PBS of the SA biosensors (*A*) to the dissociation step (*H*). The biotinylated ssDNA (Bt-ssDNA) was loaded at 0.5 µM for 60 seoncds and target dsDNA hybridization (0.5 mM) was performed over 120 seconds in PBS buffer. At the end of each immobilization step, the SA-biosensors were washed (↓ arrows). For the subsequent baseline (*F*), association (*G*) and dissociation (*H*) steps, the kinetic buffer was 25 mM Na_2HPO_4 (pH 7.5), 250 mM NaCl, 0.01 mM EDTA and 0.05% tween 20 buffer.

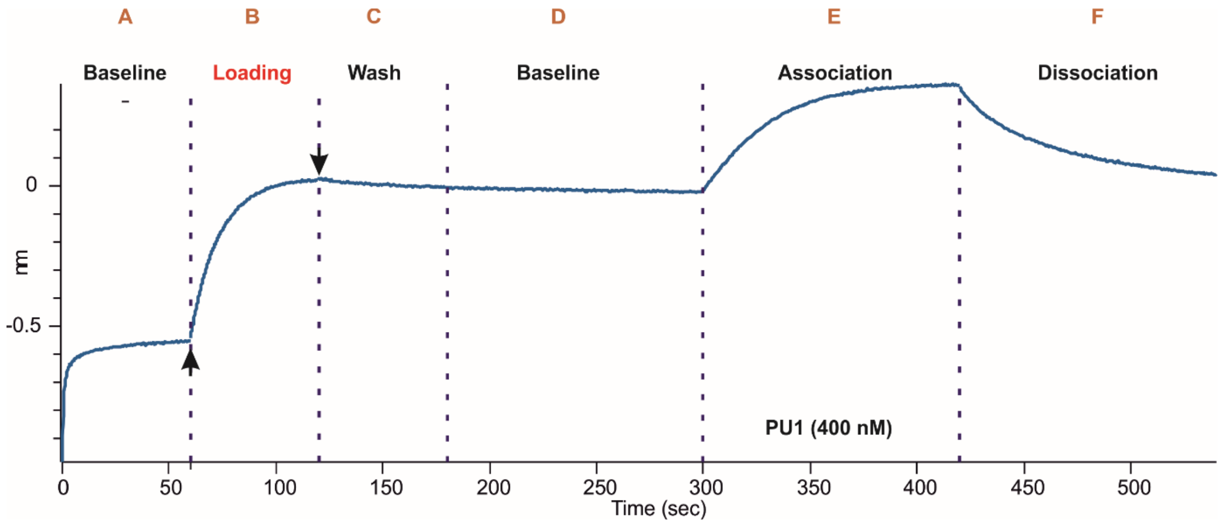

Figure S16. Sensorgrams of hairpin **29** immobilization on SA-biosensors and kinetic curves of association and dissociation exemplified at the highest PU1 concentration (400 nM). The six independent steps (*A*-*F*) are shown. The biotinylated **29** was loaded at 2 mg/mL in 25 mM Na_2HPO_4 (pH = 7.5) buffer containing 250 mM NaCl, 1 mM EDTA and 0.05 % tween. At the end of the loading step, the SA-biosensors were washed with the buffer (↓ arrows). For the subsequent baseline, association and dissociation steps, the kinetic buffer remained identical.

1.2 Supplementary tables

Table S1 Synthesized oligonucleotides, conjugates and biotinylated oligonucleotides, their calculated and found exact masses and yields. Oligonucleotides were synthesized on four cartridges with 4 µmol initial capacity each. Yields **13-21** are from ODN synthesis. Yields of **24** and **25** are from DNA-foldamer conjugation, **28** and **29** are yields of biotinylation of **20** and **21**.

Sequence name	Sequence (5'→3')	Calculated $[M\text{-}nH]^{n-}$	Found (*m/z*)	Yield (Isolated) Mass (Percent)
**13**	D-d(5'- GTT TTG **X**CA AAA C-3')	n=1; 4161.8	4162.8	13%
**14**	L-d(5'-GTT TTG **X**CA AAA C-3')	n=1; 4161.8	4161.4	11%
**15**	d(5'- ACA GGA T**X**A TCC TGT-3')	n=1; 4782.4	4782.5	12%
**16**	d(5'-pC**X**G TCC TA-3')	n=1; 2654,5	2654.0	14%
**17**	d(5'-pAGG ATG **X**C-3')	n=1; 2743,5	2742.4	14%
**18**	d(5'-CTG **X**CA GGA TG**X** CAT Cp-3')	n=1; 5383.8	5383.7	8%
**19**	d(5'-CTG T**X**A CAG GAT GT**X** ACA TCp-3')	n=1; 6614.3	6614.4	6%
**20**	d(5'-CAC ATC CTG T**X**A CAG GAT GTG-3')	n=1; 6636.2	6638,1.	10%
**21**	d(5'-GAC AGG ATG T**X**A CAT CCT GTC-3')	n=1; 6636.237	6636.5	9%
**24**	D-d(5'- GTT TTG **X(22)**CA AAA C-3')	n=4; 1624.271	1624.277	338 µg (42%)
**25**	L-d(5'-GTT TTG **X(22)**CA AAA C-3')	n=4; 1624.271	1624.363	904 µg (39%)
**28**	d(5'-CAC ATC CTG T**X**A CAG GAT GTG-3')	n = 5; 1420.687	n = 5; 1420.515	1.31 mg (67%)
**29**	d(5'-GAC AGG ATG T**X**A CAT CCT GTC-3')	n = 5; 1420.687	n = 5; 1420.515	182 µg (33%)

Table S2 Summary of X-ray diffraction and refinement data for racemic **13**/**14**. Values in parenthesis refer to data in the highest resolution shell.

Parameters	L/D-d(GT_4G)−X−d(CA_4C)
**Data collection**	
Wavelength	0.976 Å
Resolution[a] range	28.56 – 2.5 Å (2.59 – 2.5 Å)
Space group	*P*-1
Unit cell	*a* = 26.517 Å, *b* = 30.040 Å, *c* = 45.224 Å<br>*α* = 105.62°, *β* = 105.77°, *γ* = 94.34°
Total reflections	7970 (794)
Unique reflections	3985 (397)
Multiplicity	2.0 (2.0)
Completeness	90.2 % (88.9%)
Mean I/ σ (I)	13.98 (5.53)
Wilson B-factor	49.79 $Å^2$
R-merge	3.44 % (19.18 %)
R-meas	4.87 % (27.12 %)
CC1/2	0.998 (0.901)
**Refinement**	
No. of Reflections	3985 (396)
R_{work}	27.82 % (33.89 %)
R_{free}	32.79 % (38.31 %)
No. of non-H atoms<br>DNA hairpin<br>Metal ions, Solvent	739<br>560<br>4 Mg^{2+}, 175
R.m.s.d. bond<br>R.m.s.d. angle	0.014 Å<br>1.92°
Average B factor	34.25 $Å^2$
PDB ID #	8Q60

Table S3 Definition sugar structural parameters**.**

v0:	C4'-O4'-C1'-C2'
v1:	O4'-C1'-C2'-C3'
v2:	C1'-C2'-C3'-C4'
v3:	C2'-C3'-C4'-O4'
V4:	C3'-C4'-O4'-C1'
tm	the amplitude of pucker
P	the phase angle of pseudorotation

Table S4 Sugar puckering of strand 1 of hairpin 1.

Base	v0	v1	v2	v3	v4	tm	p	Puckering
1G	-17.9	-11.7	35.2	-45.5	40.6	46.2	40.5	C4'-exo
2T	-7.1	-10.8	23.7	-27.8	22.0	28.0	32.3	C3'-endo
3T	-23.6	32.4	-28.6	15.6	4.9	32.1	152.7	C2'-endo
4T	-25.2	32.1	-26.7	12.7	7.7	31.7	147.5	C2'-endo
5T	-40.2	44.8	-33.2	10.5	18.4	44.9	137.8	C1'-exo
6G	-18.9	31.1	-31.1	20.5	-1.2	32.4	163.5	C2'-endo

Table S5 Sugar puckering of strand 2 of hairpin 1.

Base	v0	v1	v2	v3	v4	tm	p	Puckering
1C	-35.5	15.0	9.80	-30.3	42.3	41.2	76.3	O4'-endo
2A	-22.8	35.9	-34.1	21.7	0.20	36.2	160.5	C2'-endo
3A	-15.2	30.7	-34.0	26.4	-7.30	34.2	173.4	C2'-endo
4A	-28.2	36.5	-30.7	15.5	7.70	35.9	149.0	C2'-endo
5A	-11.8	27.6	-31.8	26.0	-9.10	31.8	177.5	C2'-endo
6C	-30.9	36.5	-28.4	11.0	12.3	36.2	141.9	C1'-exo

Table S6 Sugar puckering of strand 1 of hairpin 2.

Base	v0	v1	v2	v3	v4	tm	P	Puckering
1G	2.0	16.7	-27.6	29.3	-20.1	29.8	202.2	C3'-exo
2T	-33.6	41.7	-34.3	15.8	10.9	41.3	146.3	C2'-endo
3T	-33.4	36.8	-26.6	7.8	15.9	36.8	136.3	C1'-exo
4T	-25.3	33.8	-28.7	14.5	6.6	33.2	149.9	C2'-endo
5T	-23.3	34.0	-31.1	18.3	2.8	34.0	156.4	C2'-endo
6G	-27.4	37.8	-35.1	20.1	4.4	38.6	155.3	C2'-endo

Table S7 Sugar puckering of strand 2 of hairpin 2.

Base	v0	v1	v2	v3	v4	tm	P	Puckering
1C	-39.1	37.0	-21.1	-0.9	24.7	39.2	122.5	C1'-exo
2A	-34.7	32.1	-18.2	-1.3	22.4	34.6	121.7	C1'-exo
3A	-15.8	30.6	-33.2	25.1	-6.2	33.5	171.6	C2'-endo
4A	-25.5	32.3	-27.1	13.2	7.3	31.9	148.1	C2'-endo
5A	-9.4	29.2	-37.0	32.7	-14.9	37.1	184.5	C3'-exo
6C	6.2	-20.6	26.5	-23.4	11.1	26.6	5.4	C3'-endo

Table S8 K_D values and related association (k_{on}) and dissociation (k_{off}) constant values calculated after global curve fitting using 1:1 model.

Compound	K_D ($s^{-1}M^{1-}$)	k_{on} ($s^{-1}M^{-1}$)	k_{off} ($s^{-1}M^{-1}$)
**26**	3.30×10^{-7}	5.43×10^{3}	1.79×10^{-3}
**28**	3.80×10^{-7}	2.65×10^{4}	1.01×10^{-2}
**29**	3.51×10^{-7}	4.35×10^{4}	1.53×10^{-2}

2 Materials and Methods

2.1.1 General methods for synthesis and analysis for small molecule intermediates, foldamers and conjugates

Reagents were used as purchased from commercial sources without further purification. Column chromatography purifications were performed on silica gel (230-400 mesh, 40-63 µm, Merck). Thin-layer chromatography was performed on silica gel plates (60-F254, Merck). Reactions requiring anhydrous conditions were performed under nitrogen dried over $CaCl_2$, with commercially anhydrous solvents unless states otherwise. Anhydrous THF and DCM for solid- and solution-phase synthesis was dispensed from a *MBRAUN SPS-800* solvent purification system using alumina columns for drying. $CHCl_3$, DIPEA, and NEt_3 were freshly distilled over CaH_2 under N_2-atmosphere.

NMR spectra were recorded on the spectrometers *Avance III HD 400 MHz Bruker BioSpin* and *Avance III HD 500 MHz Bruker Biospin* for 400 MHz and 500 MHz. $CDCl_3$ (δ_H: 7.26, δ_C: 77.0), DMSO-d_6 (δ_H: 2.50, δ_C: 39.4) and D_2O (δ_H: 4.79) were used as solvents and their residual solvent signals were used as internal standards.[42] NMR spectra of the oligomers were recorded in H_2O/D_2O 9:1 (vol/vol), 50 mM NH_4HCO_3 at 298 K. The raw data was evaluated using *Mnova version 14.0.0* by *Mestrelab Research.* The derived data signals are stated with chemical shift in ppm, their multiplicity (s = singlet, bs = broad singlet, d = doublet, t = triplet, q = quadruplet, m = multiplet, or a combination of these), their coupling constant in Hz and their integrated values. Water suppression was performed with excitation sculpting. Reactions requiring anhydrous conditions were performed under nitrogen. Analytical RP-HPLC analysis and semi-preparative purifications in acidic conditions were performed on a Thermo Fisher Scientific Ultimate 3000 HPLC system using Macherey-Nagel Nucleodur C18 columns (4.6 × 100 mm, 5 µm and 10 × 250 mm, 5 µm). When using acidic conditions (TFA buffered system), 0.1% TFA was added to aqueous mobile phase (referred to as mobile phase A) and to acetonitrile (referred to as mobile phase B). When using basic conditions, the column used for analysis was Macherey-Nagel Nucleodur C18 (4.6 × 100 mm, 5 µm). The mobile phase was composed of 12.5 mM TEAA in water at pH 8.5 (A) and 12.5 mM TEAA in water: acetonitrile mixture (1:2, v/v) at pH = 8.5 (B) (*TEAA buffer system*) or 12.5 mM aqueous NH_4OAc buffer adjusted to pH = 8.5 with aqueous ammonia (A) and acetonitrile (B) *(NH_4OAc-buffer system*). For analytical runs a flow rate of 1.0 mL/min was applied; semi-preparative RP-HPLC purification were performed at a flow rate of 5.0 mL/min. UV absorbance was monitored at 254 and 300 nm with a diode array detector if not stated otherwise. LC-MS chromatograms for acidic conditions were recorded on a Thermo Scientific Dionex UltiMate 3000 equipped with a C18 gravity (2.1 x 50 mm) with a flow of 0.33 mL min-1. 0.1% of formic acid in water (A) and 0.1% of formic acid in acetonitrile (B) were used as mobile phase. For basic conditions (e.g. water soluble oligomers and DNA strands) the column used was a Kinetex C18 EVO column (2.1 x 50 mm, 1.8 µm) with 12.5 mM aqueous NH_4OAc buffer adjusted to pH 8.5 (A) and LC-MS grade acetonitrile (B). Elution was monitored by UV detection at 254 and 300 nm with a diode array detector. The LC system was coupled to a micrOTOF II mass spectrometer by Bruker Daltonics and molecules were ionized by ESI (for molecules **22**, **22a**, **23**, **24**, **25**, **28**, **29**). Circular dichroism (CD) spectra were measured on Jasco J-810 spectrometer. Spectra were measured at 25 °C, over a wavelength range of 300–500 nm, with a response time of 1 s, a continuous scanning mode, sensitivity set to standard and a scanning speed of

50 nm/min for 2 scans. Both foldamer and conjugate were solubilized in NH_4HCO_3 (50mM, pH = 8.5) and were measured at Δε values were normalized for the number of quinoline units. UV-spectroscopic measurements for determining protein, nucleic acid or foldamer concentrations were performed on a NanoDrop™ OneC (ThermoFisher Scientific). Variable temperature UV-Vis measurements nucleic acid melting were conducted on a JASCO V-650 spectrometer at 260 nm using a 10 mm quartz cuvette with a scanning rate of 1ºC/min or on a Jasco J-1500 spectrometer with a 2 mm cuvette.

2.1.2 General methods for nucleic acid synthesis and purification

DNA strands not containing X were purchased from Sigma-Aldrich and used without further purification. Phosphoramidites of canonical deoxyribonucleosides (Bz-dA-CE, Ib-dG-CE, Ac-dC-CE and dT-CE) were purchased from Sigma-Aldrich or LinkTech. UltraMild L-DNA phosphoramidites (beta-L-Pac-dA-CE, beta-L-iPr-Pac-dG-CE, beta-L-Ac-dC-CE, beta-L-dT-CE) were purchased from Glen Research. Oligonucleotides were synthesized on a 1 or 4 µmol scale using high Load Glen UnySupport™ or 3'-CPR II CPG (for 3' phosphate modifications) as solid supports using an DNA automated synthesizer (Applied Biosystems 394 DNA/RNA Synthesizer) with a standard phosphoramidite chemistry. For the linker incorporation, the linker was dissolved in MeCN/DCM (v/v; 1:9) to a 0.15 M concentration and the coupling times were extended to 310 s. Oligonucleotides were synthesized in DMT-OFF mode using DCA as a deblocking agent in CH_2Cl_2, Activator 42® or ETT as activator in MeCN, Ac_2O (or Pac_2O for UltraMild phosphoramidites) as capping reagent in pyridine/THF and I_2 as oxidizer in pyridine/H_2O. The cleavage and deprotection of the CPG bound oligonucleotides were performed in an aqueous solution of 30% NH_4OH and heating at 50ºC for 5 h. Subsequently, the supernatant was collected, and the beads were washed with water (3 × 0.3 mL). The combined aqueous solutions were concentrated under reduced pressure using a SpeedVac concentrator and subsequently lyophilized. The oligonucleotides were further purified by semi-preparative RP-HPLC using a 1260 Infinity II Manual Preparative LC System from Agilent (G7114A detector) equipped with the column VP 250/10 Nucleodur 100-5 C18ec from Macherey Nagel. A flow rate of 5 mL/min with varying gradients between 0% to 30-60% of buffer B in 45 min was applied for the purifications. The following buffer system was used: buffer A: 100 mM NEt_3/HOAc (pH 7.0) in H_2O and buffer B: 100 mM NEt_3/HOAc (pH 7.0) in H_2O/MeCN (v/v; 1:4). The purified oligonucleotides were analyzed by analytical RP-HPLC on a 1260 Infinity II LC System from Agilent (G7165A detector) equipped with the column EC 250/4 Nucleodur 100-3 C18ec from Macherey Nagel using a flow of 1 mL/min and gradients of 0% to 30-60% of buffer B in 45 min. Finally, the purified oligonucleotides were desalted using a C18 RP-cartridge from Waters. The absorbance of the synthesized oligonucleotides in H_2O solution were measured using an IMPLEN NanoPhotometer® N60/N50 at 260 nm. The extinction coefficients of the oligonucleotides were calculated using the OligoAnalyzer Version 3.0 from Integrated DNA Technologies. For oligonucleotides containing the foldamer linker an estimated extinction coefficient of 11000 $M^{-1}cm^{-1}$ was used for the calculations. The structural integrity of the synthesized oligonucleotides was analyzed by MALDI-TOF mass measurement. For this purpose, the synthesized oligonucleotides **13-21** (2-3 µL) were desalted on a 0.025 µm VSWP filter (Millipore), co-crystallized in a 3-hydroxypicolinic acid matrix (HPA, 1 µL) and measured on a Bruker Autoflex II. Biotinylated strands were measured on a microTOF II mass spectrometer by Bruker Daltonics and molecules were ionized by ESI.

2.1.3 Crystallography

2.1.3.1 Crystallization and data collection

The lyophilized powder of **13** D-d(GT_4G)−X−d(CA_4C) was dissolved using ultra-pure water such that the final concentration was 1mM. Crystallization screening was carried out by sitting drop vapor diffusion technique at 293 K. Multiple crystallization hits (> 10) were obtained from the screens and one condition that was reproducible was optimized for XRD studies. Colorless, prisms (Figure S4) were obtained within three days from drops prepared by mixing 1µL of **13** D-d(GT_4G)−X−d(CA_4C) and 1µL of crystallization reagent containing 10% v/v (+/-)-2-Methyl-2,4-pentanediol (MPD), 40 mM Sodium cacodylate trihydrate buffer (pH 6.0), 12 mM Spermine tetrahydrochloride, 80 mM Sodium chloride and equilibrated against a reservoir of 30% v/v MPD (50 µL). Crystals were fished using microloops and plunged into liquid nitrogen directly without further cryoprotection.

The diffraction experiment was performed on the beamline P13[43] operated by EMBL Hamburg, at the Petra III storage ring (DESY, Hamburg). Crystal characterization was carried out using 4 images at T = 100 K and λ = 0.775 Å, exposure time for 0.04 s and 1° oscillation per frame. However, the characterization revealed weak, DNA fiber diffraction-like pattern.

We eventually used the racemic crystallographic approach for DNA[25] to improve the quality of crystal and diffraction. The L-enantiomer of d(GT_4G)−X−d(CA_4C) **14** was thus synthesized and the lyophilized powder was dissolved in ultra-pure water. The racemic mixture of L/D-d(GT_4G)−X−d(CA_4C) was prepared by mixing the enantiopure solutions to a final concentration of 1 mM. Colorless needles (Figure S4) of **13/14** L/D- d(GT_4G)−X−d(CA_4C) were obtained within 7 days by hanging drop vapor diffusion technique at 293 K from drops prepared by mixing 1.25 µL of L/D-d(GT_4G)−X−d(CA_4C) and 1.25 µL of the reservoir solution (400 µL) containing 50% (v/v) Polyethylene glycol 200, 100 mM MES buffer (pH 6.5) and 200 mM Magnesium chloride. Crystals were fished using microloops and plunged into liquid nitrogen directly without further cryoprotection.

The diffraction experiment was performed on the beamline P14 operated by EMBL Hamburg, at the Petra III storage ring (DESY, Hamburg). Diffraction data was measured at T = 100 K and λ = 0.976 Å (12.7 keV) using a DECTRIS EIGER2 X 16M detector. The crystal was exposed for 0.08 s and 0.1° oscillation per frame and a rotation pass of 360°. Diffraction was processed using the *autoPROC* pipeline.[44-47] The crystal belonged to the centrosymmetric space group *P*-1 and selected crystal data and data collection parameters are summarized in Table S2. The crystallographic asymmetric unit consisted of two molecules.

2.1.3.2 Structure determination and refinement

The structure was determined by molecular replacement (MR) method using the program *Phaser*[48] within the *CCP4* suite.[49] The crystal structure of D-d(GT_4G)−Sd2−d(CA_4C) (PDB ID: 1PUY)[23] was used to build a search model of **13** such that the stilbene diether linker atoms were replaced by diquinoline linker atoms. During energy-minimization, full positional restraints were put on DNA atoms (for parameters, see modeling section). The stem residues were numbered G1, T2, T3, T4, T5, G6 in strand 1 and C8, A9, A10, A11, A12 and C13 in strand 2, and diquinoline linker X is residue 7.

The best MR solution was obtained with a translation-function Z (TFZ) score of 10.4 and a log-likelihood gain (LLG) score of 274 revealed two D-d(GT_4G)−X−d(CA_4C) hairpins (named hereafter Hairpins 1

and 2). Graphical analyses of the model and the electron density maps were carried out using *Coot*.[50] The initial maps showed clearly resolved electron density for the DNA duplex portions d(GT_4G), d(CA_4C); and the diquinoline linker X. The initial map also allowed unambiguous identification of the chirality of symmetry related D- and L- enantiomers (Figure S6).

Refinement of the coordinates was carried out initially with rigid-body refinement and subsequently in combination of translation libration screw (TLS) refinement[51] using d(GT_4G) and d(CA_4C) as a separate TLS groups and restrained refinement in the program *REFMAC5*[52] with maximum-likelihood targets and the *REFMAC5* dictionary.[53] 10% of the unique reflections were used to calculate R_{free}.[54] The planar conformation of the diquinoline linker moiety was modelled using σ-weighted omit and $2F_o$-F_c electron density maps. *PRODRG* was used for the generation of diquinoline linker ligand restraint dictionary[55] and used for refinement in *REFMAC5*. An example of the final electron density are depicted in Figure S7. Beside the synthetic hairpin DNA, four Mg^{2+} ions and 175 solvent molecules were identified. Two out of four Mg^{2+} hexahydrate coordination were identified. The complete model was subsequently refined to yield final values for the R_{factor} and R_{free} of 27.82 % and 32.79 %, respectively. Further refinement did not lead to a better convergence nor an improvement in the refinement statistics. The refinement *R*-values were high compared to typical macromolecular structure depositions. Poor refinement statistics for centrosymmetric crystal are inherent to centric data.[56]

The coordinates and structure factors have been deposited in the Protein Data Bank[57] (PDB ID: 8Q60). Selected refinement parameters are given in Table S2. Figures for the structure were prepared using *PyMOL*.[58] Root mean square deviation (r.m.s.d.) values were determined using *Superpose*.[28] Structural parameters of DNA were calculated using *3DNA*.[59]

2.1.4 Molecular modeling

The molecular models of conjugates and DNA hairpins were built in Maestro (Version 11.5)[60] and energy-minimized with parameters in table S2. Hairpin models were built based on the crystal structure of stilbene-linked ds-DNA with the same stem d(GT_4G)−Sd2−d(CA_4C) (PDB ID: 1PU)[23] with full positional restraints on DNA atoms. Conjugate models were built based on the crystal structure of **13**/**14** and subsequently energy-minimized with full positional restraints on DNA atoms. Foldamer coordinates for the models of conjugates were derived from earlier crystal structures from diethylester-protected polyphosphonates,[1] stripped of their side-chain protecting groups and energy-minimized according to parameters below.

Table S 9 Minimization parameters used to build molecular models.

Forcefield	OPLS3
Solvent	Water
Charges from	Force Field
Cutoff	Extended
Method	PRCG or TNCG
Converge on	Gradient
Convergence threshold	0.01
Minimization mode	Minimization of non-conformers
Maximum iterations	20000

2.1.5 Recombinant protein expression and purification

The pET28a (+)-SAP1 plasmid for SAP1 (UniProt accession number: P28324, aa 1 - 93) with a N-terminal hexa-histidine tag overexpression was obtained from GeneScript.[38] Similarly, the pET28a (+)-PU.1plasmid for PU.1(UniProt accession number: P17947, aa 165-270) with a N-terminal hexa-histidine tag overexpression was obtained from GeneScript.[37] For overexpression and purification, we followed exactly the same protocol. The full-length proteins were expressed in *E. coli* BL21 plysS cells. Overnight pre-cultures in Luria broth (LB) supplemented with 50 µg/ml kanamycin were diluted 1000- fold with fresh 4L LB media and grown at 37 °C until OD600 reached 0.6. The expression was induced by addition of isopropyl 1-thio-β-D-galactopyranoside to make the final concentration 0.5 mM, and the culture was incubated for 15 hours at 22 °C. Next, the cells were harvested at 4000 rpm (J-LITE® JLA9.1000 Rotor, Beckman Coulter) and stored at −80 °C. The following purification steps were carried out at 4°C. Briefly, the cells were resuspended in 50 mL of 20 mM HEPES buffer pH 7.5, 500 mM NaCl containing 20 mM imidazole, and 1 mM phenylmethylsulfonyl fluoride (PMSF). The cells were lysed by sonication and the lysate was clarified by centrifugation at 18000 rpm (JA-25.5 Rotor, Beckman Coulter) for 40 min. The supernatant was equilibrated with nickel-nitrilotriacetic acid (Ni-NTA) agarose beads by gentle mixing for 1 hour. The mixture was then applied to a gravity flow column, drained out, and washed with 20mM HEPES,500mM NaCl, and 100mM imidazole. The protein was eluted with 20mM HEPES, 500mM NaCl, and 400mM imidazole, and immediately dialysed with 20mM HEPES, 150mM NaCl buffer. For storage, the protein was concentrated, frozen in liquid nitrogen and stored in a -80 °C freezer and thawed prior to BLImeasurements.

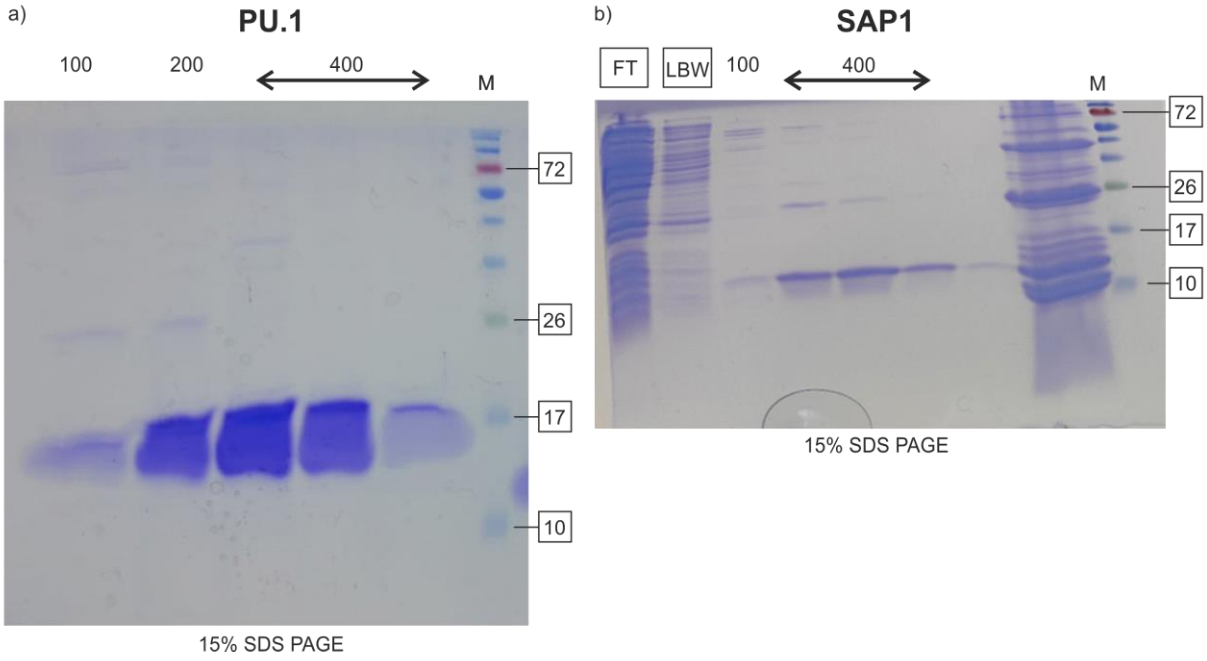

Figure S17. a) SDS-PAGE of PU.1 analysis. lane1: lysis buffer+100mM imidazole wash; lane 2: lysis buffer + 200 mM imidazole wash; lane 3-5: lysis buffer + 400 mM imidazole elution; lane 6: molecular weight marker (kDa). b) SDS-PAGE of SAP1 analysis. lane1: flow through (FT); lane 2: lysis buffer wash (LBW); lane3: lysis buffer + 100mM imidazole wash, lane 4-7: lysis buffer + 400mM Imidazole elution; lane 8: different sample; lane 9: molecular weight marker (kDa).

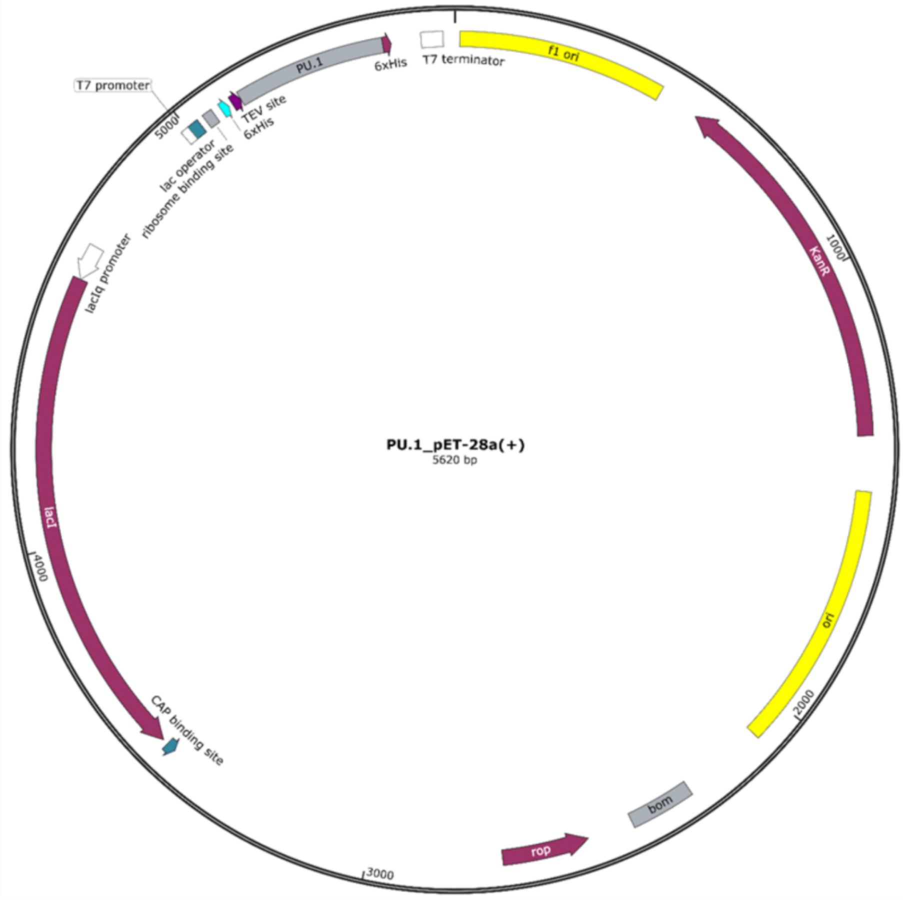

ATGGGCAGCAGCCATCATCATCATCATCAC AGCAGCGGCGAGAATCTTTATTTTCAGGGCCATATGGGTAGCAAGAAGAAGATTCG
TCTGTATCAGTTCCTGCTGGACCTGCTGCGTAGCGGCGACATGAAAGATAGCATTTGGTGGGTGGACAAAGACAAGGGTACCTTC
CAGTTTAGCAGCAAACACAAAGAGGCGCTGGCGCACCGTTGGGGTATCCAGAAAGGCAACCGTAAGAAAATGACCTACCAAAAG
ATGGCGCGTGCGCTGCGTAACTATGGTAAAACCGGCGAAGTGAAGAAAGTTAAGAAAAAGC TGACCTACCAATTTAGCGGTGAA
GTGCTGGGTCGTGGTGGTCTGGCGGAGCGTCGTCATCCGCCGCAT

MGSSHHHHHHSSGENLYFQ |GHMGSKKKIRLYQFLLDLLRSGDMKDSIWWVDKDKGTFQFSSKHKEALAHRWGIQKGNRKKMTYQK
MARALRNYGKTGEVKKVKKKLTYQFSGEVLGRGGLAERRHPPH

Figure S18. pET28a(+) plasmid map (top), coding sequence (middle), and “PU.1” protein expression product (bottom) used in this study; **|** indicates the TEV protease cleavage site.

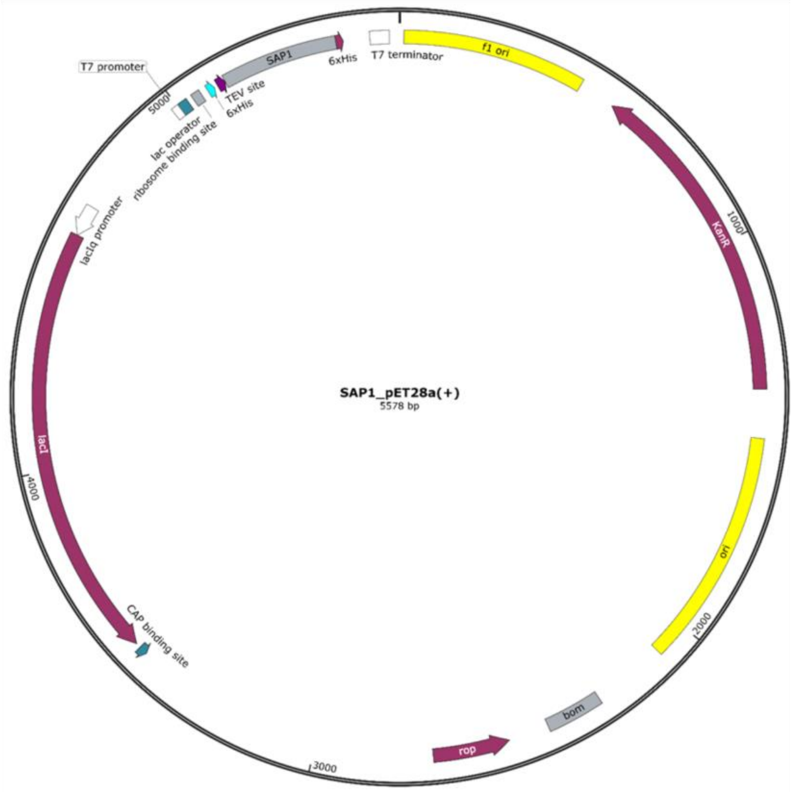

ATGGGCAGCAGC CATCATCATCATCATCAC AGCAGCGGC GAGAATCTTTATTTTCAGGGC CATATGGACAGCGCGATCACCCTGTGGC
AATTCCTGCTGCAGCTGCTGCAAAAGCCGCAGAACAAACACATGATTTGCTGGACCAGCAACGACGGTCAATTTAAGCTGCTGCAAG
CGGAGGAAGTGGCGCGTCTGTGGGGCATCCGTAAGAACAAACCGAACATGAACTATGATAAGCTGAGCCGTGCGCTGCGTTACTACT
ACGTGAAGAACATCATTAAGAAAGTTAACGGTCAGAAGTTCGTGTATAAATTTG TTAGCTACCCGGAGATTCTGAACATG

MGSSHHHHHHSSGENLYFQ | GHMDSAITLWQFLLQLLQKPQNKHMICWTSNDGQFKLLQAEEVARLWGIRKNKPNMNYDKLSRALRYY
YVKNIIKKVNGQKFVYKFVSYPEILNM

Figure S19. pET28a(+) plasmid map (top), coding sequence (middle), and “SAP1” protein expression product (bottom) used in this study; **|** indicates the TEV protease cleavage site.

2.1.6 Bio-layer interferometry

2.1.6.1 Biotinylated ssDNA immobilization and dsDNA hybridization on streptavidin (SA) bionsensors and kinetic analysis with PU1 and SAP1proteins as analytes.

BLI measurements were carried out at 25 °C on an Octet R8 BLI Sartorius instrument, using streptavidin (SA) biosensors. To perform the ssDNA immobilization and hybridization, we took inspiration from the paper of Yang *et al.* were the authors reported SPR studies of DNA hybridization and protein-DNA binding.[61] The binding strongly depends on NaCl concentration and that [NaCl] was adjusted so that the binding and release kinetics can conveniently be monitored by BLI. All the steps, for setting-up these specific experiments, are exemplified in the figure S14. The buffer for the assembly of biotinylated ssDNA and dsDNA hybridization was PBS. After a baseline in buffer for 60 s (*A*), the biotinylated ssDNA **26** was loaded on the biosensors (6) with a loading at 0.5 μM over 60 sto reach a 1-0.8 nm shift (*B*). The biosensors were then washed with PBS buffer for 60 sec (*C*), and the subsequent target DNA hybridization of the immobilized ssDNA was performed with a 0.5 μM target DNA for 120 sec to yield a signal plateau (*D*). The biosensors were washed again in PBS (*E*) before to record a second baseline for 120 s in the kinetic buffer: 25 mM Na_2HPO_4 (pH 7.5) containing 250 mM NaCl, 1 mM EDTA, 0.05% tween 20 (*F*). The association was next recorded for 120 sec with a range of PU1 concentration from 400 nM to 25 nM (*G*). This protein, being known to be prone to aggregation, we did not try to go higher in concentration.[62] For the dissociation, the sensors were moved back and dipped in the wells were the baseline was recorded (H). A global curve fitting with a 1:1 binding model was next performed using the reprocessing software embedded with the BLI Octet R8 instrument. The same approach was followed for the control dsDNA **27**. In the case of protein SAP1 as analyte, the same two phases loading process of the dsDNA was followed and the protein was assayed for its binding to dsDNA ligand sequences **26** and **27**, in the same kinetic buffer, in a protein concentration ranging from 2 to 0.125 mM. A global curve fitting was next performed to calculate the K_D values.

2.1.6.2 Loading of biotinylated hybrid hairpins **28** and **29** on SA-biosensors and kinetic assays

Biotinylated hairpins **28** and **29** were independently loaded on SA-biosensors and measurements for **29** are depicted in figure S15. After a baseline in 25 mM Na_2HPO_4 (pH 7.5) buffer (step *A*) containing 250 mM NaCl, 1 mM EDTA, 0.05% tween 20 for 60 sec, loading was performed at 2 µg/ml concentration of hybrid over 60 s (*B*). The sensors were then washed in the same buffer (*C*), a second baseline for 120 s was performed (*D*) and the association step was performed at different protein concentrations, from 500 nM to 25 nM for PUI and 2 μM to 0.125 μM for SAP1 using the same kinetic buffer (*E*). The dissociation step was recorded in the same well column were the second baseline was performed (*F*). The K_D values were calculated by global curve fitting with a 1:1 binding model using the reprocessing software embedded with the BLI instrument.

3 Synthetic procedures

3.1 Linker synthesis

3.1.1 Synthesis of 3'-terminal quinoline ring

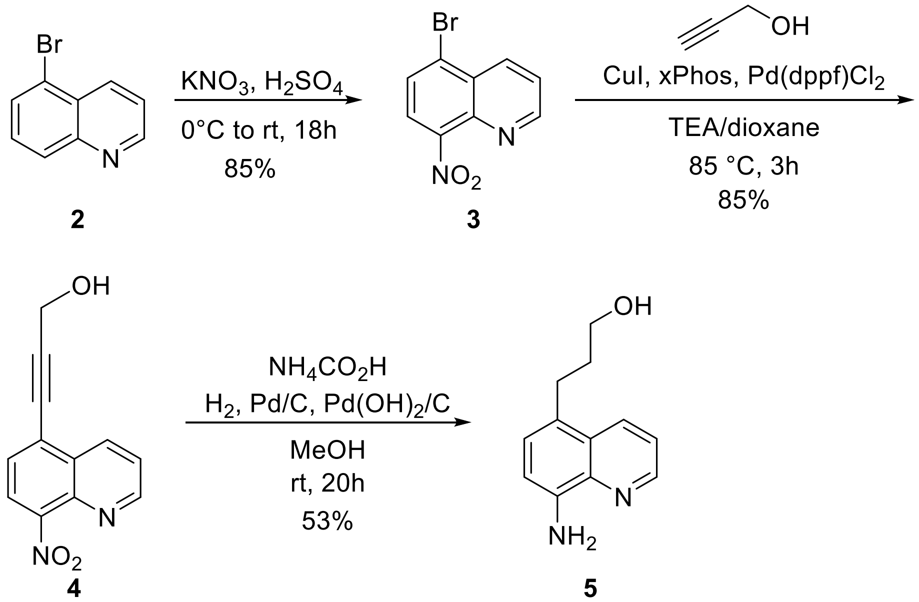

Compound **3**[63]

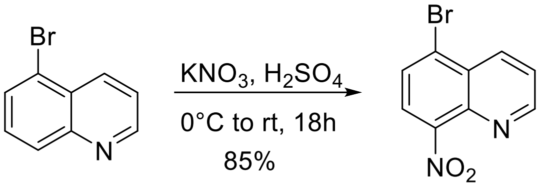

5-Bromoquinoline(**2**) (3.00 g, 14.4 mmol, 1.0 eq.) was dissolved in sulfuric acid (96%, 12.0 mL) and cooled to 0 °C. KNO_3 (2.33 g, 23.1 mmol, 1.6 eq.) was added portion wise over 15 min at 0°C and the reaction was stirred at rt for 22 h. The reaction mixture was poured on ice, filtered and washed with water. The product was lyophilized to give the title compound 1 as a yellow solid (3.11 g, 12.3 mmol, 85%).

^{1}H NMR (500 MHz, DMSO-*d*$_6$) δ [ppm] = 9.12 (dd, *J* = 4.2, 1.5 Hz, 1H), 8.68 (dd, *J* = 8.6, 1.5 Hz, 1H), 8.26 (d, *J* = 8.0 Hz, 1H), 8.19 (d, *J* = 8.1 Hz, 1H), 7.90 (dd, *J* = 8.6, 4.2 Hz, 1H).

Compound **4**

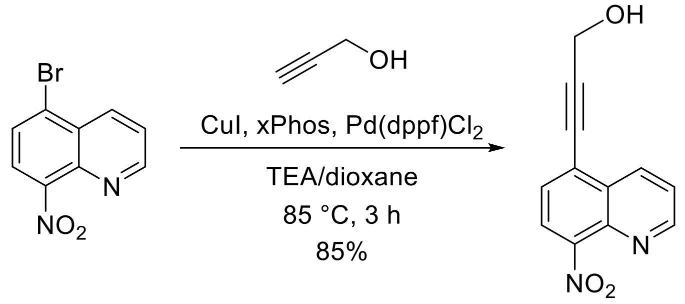

3 (1.74 g, 6.86 mmol, 1.0 eq.), XPhos (327 mg, 686 µmol, 0.1 eq.) and Copper(I) iodide (131 mg, 686 µmol, 0.1 eq.) were suspended in triethylamine (67.0 mL) and dioxane (67.0 mL) under N_2-atmosphere. The mixture was degassed for 10 min and propargyl alcohol (599 µl, 10.3 mmol, 1.5 eq.) and Pd(dppf)Cl_2 (502 mg, 686 µmol, 0.1 eq.) were added and the suspension further degassed for 10 min. The mixture was heated to 85 °C under reflux and N_2-atmosphere for 3 h.The solvent was removed *in vacuo*. The crude product was purified by column chromatography (EtOAc 50 → 70% EtOAc in cHex) to give the title compound as an orange solid (1.33 g, 5.83 mmol, 85%).

^{1}H NMR (500 MHz, DMSO-*d*$_6$) δ [ppm] = 9.11 (dd, *J* = 4.2, 1.7 Hz, 1H), 8.76 (dd, *J* = 8.5, 1.6 Hz, 1H), 8.28 (d, *J* = 7.8 Hz, 1H), 7.90 (d, *J* = 7.8 Hz, 1H), 7.87 (dd, *J* = 8.5, 4.2 Hz, 1H), 5.60 (t, *J* = 6.0 Hz, 1H), 4.50 (d, *J* = 6.0 Hz, 2H).

^{13}C NMR (126 MHz, DMSO-*d*$_6$) δ [ppm] = 153.3, 147.5, 138.3, 134.5, 129.5, 128.6, 124.3, 124.2, 123.05, 99.0, 79.2, 49.7.

HRMS: (ESI$^+$) m/z calc. for $C_{12}H_9N_2O_3$: 229.0608 [M+H]$^+$; found: 229.0606.

Compound **5**

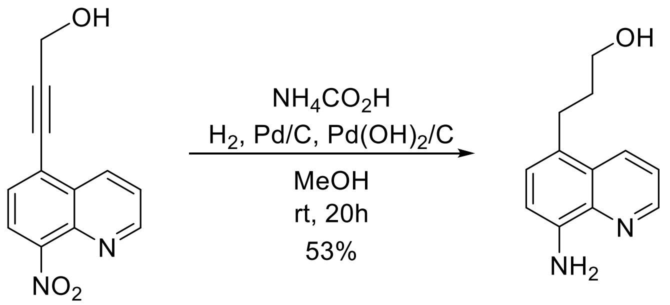

4 (767 mg, 3.36 mmol, 1 eq.) was dissolved in MeOH (150 mL) and degassed for 10 min by bubbling N_2 threw the solution. Pd/C (10% w/w, 150.0 mg) and NH_4HCO_2 (2.12 g, 33.6 mmol, 10 eq.) was added and the black suspension was further degassed for 10 min. The reaction mixture was vigorously stirred under H_2-atmosphere at rt for 20h. The black solid was filtered over celite and washed with MeOH (150.0 mL). The solvent was removed *in vacuo*. The crude product was purified by automated reversed-phase column chromatography (C18, 5 → 100% MeCN in H_2O) and further purified by column chromatography (20 → 40% acetone in DCM) to give the title compound (360 mg, 1.78 mmol, 53%) as a yellow oil.

^{1}H NMR (500 MHz, DMSO-d_6) δ [ppm] = 8.72 (dd, *J* = 4.1, 1.6 Hz, 1H), 8.34 (dd, *J* = 8.5, 1.7 Hz, 1H), 7.48 (dd, *J* = 8.5, 4.1 Hz, 1H), 7.13 (d, *J* = 7.7 Hz, 1H), 6.79 (d, *J* = 7.7 Hz, 1H), 5.72 (s, 2H), 4.48 (t, *J* = 5.1 Hz, 1H), 3.45 (td, *J* = 6.3, 5.1 Hz, 2H), 2.94 – 2.84 (m, 2H), 1.72 (ddt, *J* = 9.2, 7.7, 6.4 Hz, 2H).
^{13}C NMR (126 MHz, DMSO-d_6) δ [ppm] = 146.4, 143.5, 137.9, 132.3, 127.1, 126.8, 124.3, 121.0, 108.3, 60.3, 34.3, 27.4.
HRMS: (ESI$^+$) m/z calc. for $C_{12}H_{15}N_2O$: 203.1179 [M+H]$^+$; found: 203.1177.

3.1.2 Synthesis of 5'-terminal quinoline ring

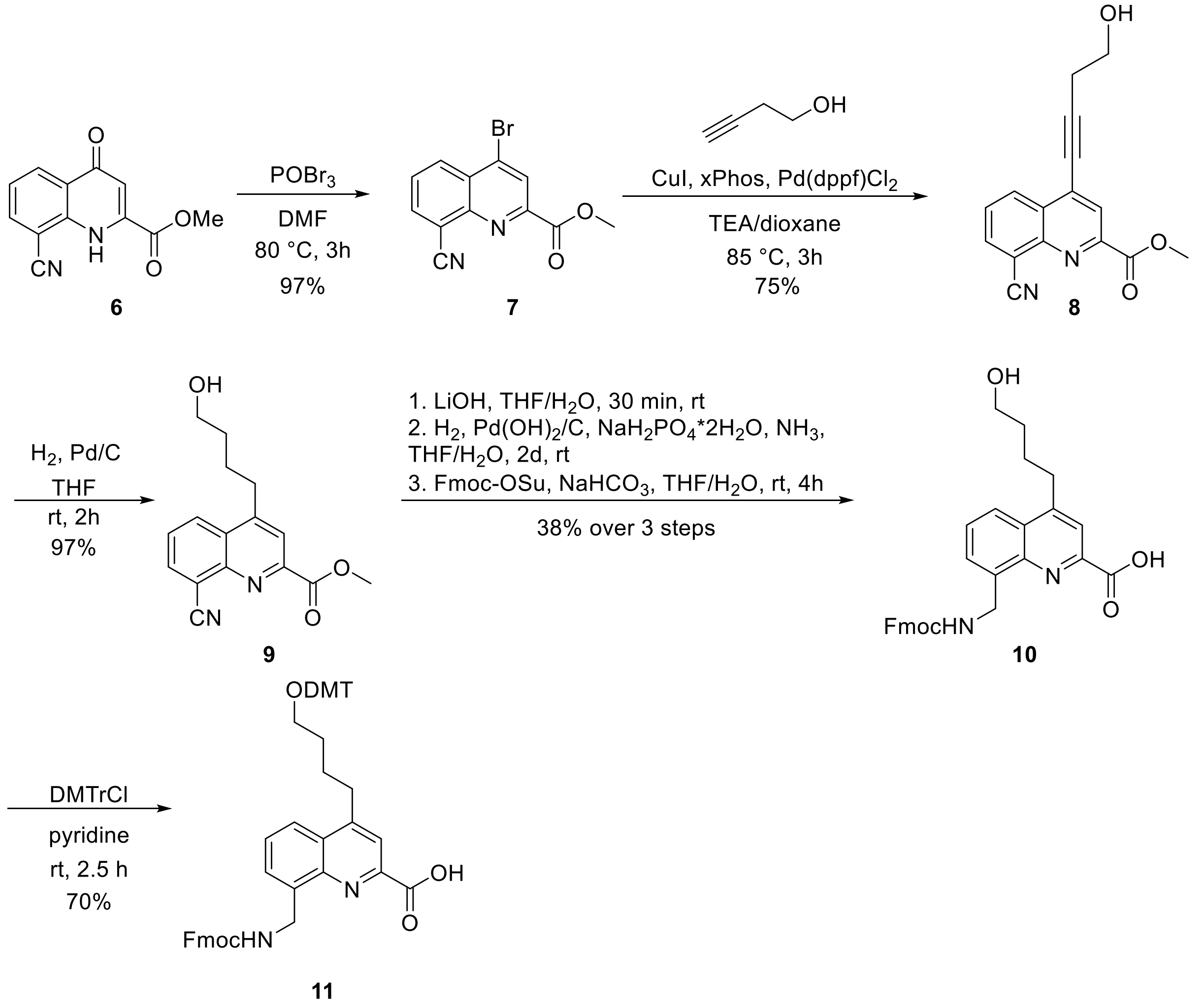

Compound **7**

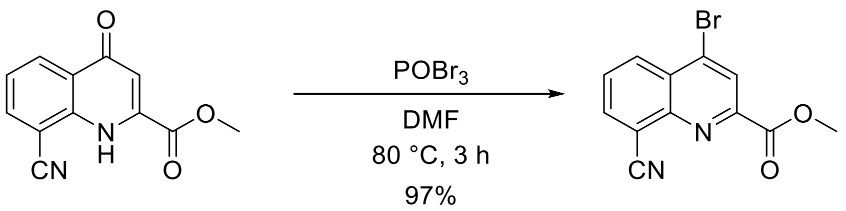

6 (4.20 g, 16.8 mmol, 1.0 eq.) was dissolved in anhydrous DMF (50 mL) under N_2-atmosphere and $POBr_3$ (5.28 g, 18.4 mmol, 1.1 eq.) was added. The solution was vigorously stirred at 80 °C for 3 h under N_2-atmosphere to form a colorless precipitate. After cooling to rt, the reaction mixture was poured on ice and stirred for 30 min. The solid was filtered and washed well with H_2O until the washing solution gave

a neutral pH and the residual solid dried in a vacuum oven at 60 °C over night (4.75 g, 16.3 mmol, 97%) as an off-white solid.

^{1}H NMR (500 MHz, $CDCl_3$) δ [ppm] = 8.60 (s, 1H), 8.51 (dd, *J* = 8.5, 1.4 Hz, 1H), 8.27 (dd, *J* = 7.2, 1.4 Hz, 1H), 7.83 (dd, *J* = 8.6, 7.2 Hz, 1H), 4.10 (s, 3H).

^{13}C NMR (126 MHz, $CDCl_3$) δ [ppm] = 164.5, 149.6, 147.2, 137.43, 136.0, 131.85, 129.1, 126.8, 116.2, 115.0, 53.8.

HRMS: (ESI^+) m/z calcd. for $C_{12}H_7N_2O_2BrNa^+$ 312.9583 $[M+Na]^+$; found: 312.9582.

Compound **8**

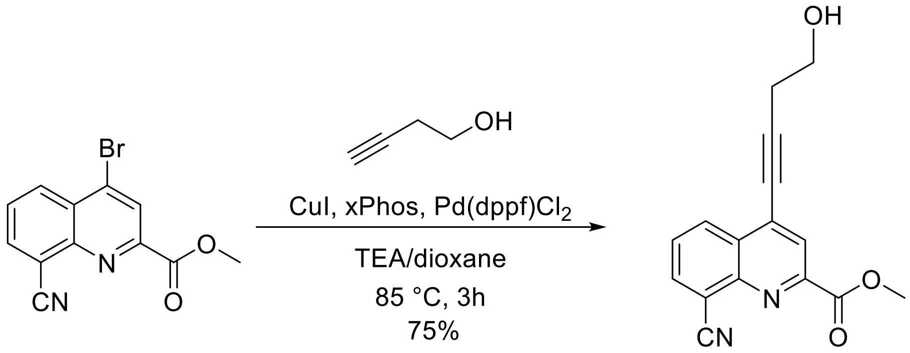

7 (4.75 g, 16.3 mmol, 1.0 eq.), XPhos (778 mg, 1.63 mmol, 0.1 eq.) and Copper(I) iodide (321 mg, 1.63 mmol, 0.1 eq.) were suspended in triethylamine (150 mL) and dioxane (150 mL) under N_2-atmosphere. The mixture was degassed for 10 min and but-3-yn-1-ol (1.85 mL, 24.5 mmol, 1.5 eq.) and Pd(dppf)Cl_2 (1.19 g, 1.63 mmol, 0.1 eq.) were added and the suspension further degassed for 10 min. The mixture was heated to 85 °C under reflux and N_2-atmosphere for 3 h to give a color gradient from yellow to orange to red to black.The solvent was removed *in vacuo*. The crude product was purified by column chromatography (80% EtOAc in cHex) and then precipitated from DCM (35 mL)/MeOH (35 mL) by slow evaporation of the DCM under reduced pressure. The title compound was isolated by filtration as an off-white solid (3.44 g, 12.2 mmol, 75%).

^{1}H NMR (500 MHz, $CDCl_3$) δ [ppm] = 8.55 (dt, *J* = 8.4, 1.4 Hz, 1H), 8.33 (s, 1H), 8.21 (dt, *J* = 7.2, 1.3 Hz, 1H), 7.75 (ddd, *J* = 8.4, 7.2, 1.3 Hz, 1H), 4.08 (s, 3H), 3.97 (q, *J* = 6.2 Hz, 2H), 2.90 (t, *J* = 6.3 Hz, 2H), 1.87 (t, *J* = 5.9 Hz, 1H).

^{13}C NMR (126 MHz, $CDCl_3$) δ [ppm] = 165.3, 149.6, 146.8, 136.9, 132.7, 131.1, 129.2, 128.21, 125.4, 116.6, 114.8, 100.4, 77.22, 60.9, 53.6, 24.3.

HRMS: (ESI^-) m/z calc. for $C_{16}H_{11}N_2O_3^-$ $[M-H]^-$: 279.0775; found: 279.0772.

Compound **9**

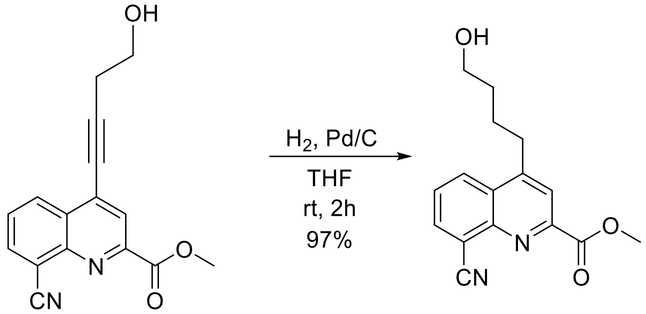

8 (3.44 g, 12.3 mmol, 1 eq.) was dissolved in THF (90 mL). The solvent was degassed by bubbling N_2 threw the solution for 15 min and put under N_2-atmosphere. To this, Pd/C (360 mg) was added and the N_2-atmopshere was replaced by H_2-atmosphere. The black suspension was stirred under H_2-atmosphere for 2 h at rt. The suspension was filtered through celite and the solvent was removed *in vacuo* then co-evaporated with toluene (1x) and Et_2O (1x) to give the title compound as a yellow solid (3.39 g, 11.9 mmol, 97%) as a yellow solid.

^{1}H NMR (500 MHz, $CDCl_3$) δ [ppm] = 8.35 (dd, *J* = 8.5, 1.3 Hz, 1H), 8.26 – 8.10 (m, 2H), 7.73 (dd, *J* = 8.6, 7.2 Hz, 1H), 4.08 (s, 3H), 3.74 (td, *J* = 6.2, 4.8 Hz, 2H), 3.27 – 3.14 (m, 2H), 1.91 (tt, *J* = 9.4, 7.6 Hz, 2H), 1.81 – 1.67 (m, 2H), 1.32 (t, *J* = 5.0 Hz, 1H).

^{13}C NMR (126 MHz, $CDCl_3$) δ [ppm] = 165.9, 151.2, 149.6, 147.1, 136.3, 128.8, 128.7, 127.5, 122.3, 117.0, 115.2, 62.5, 53.5, 32.4, 32.1, 26.6.

HRMS: (ESI^-) m/z calc. for $C_{16}H_{11}N_2O_3^-$ $[M-H]^-$: 285.1088; found: 283.1086.

Compound **10**

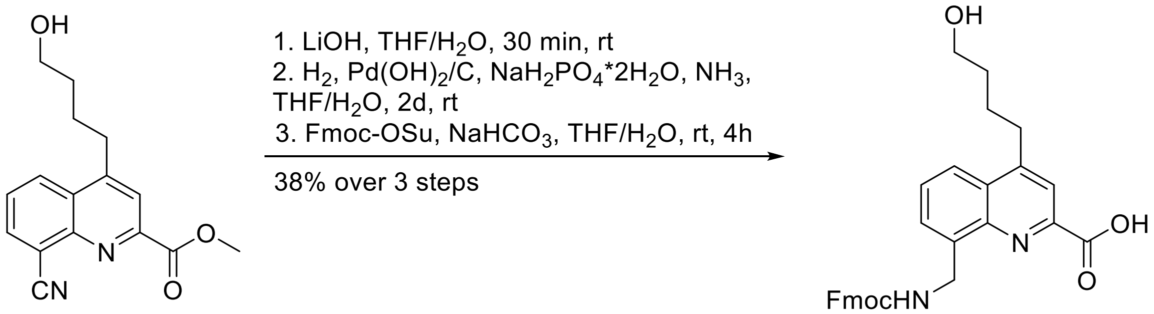

9 (3.38 g, 11.9 mmol, 1 eq.) was dissolved in THF (200 mL). LiOH (370 mg, 15.5 mmol, 1.3 eq.) dissolved in water (100 mL) was added and the reaction mixture was stirred at rt for 30 min. The mixture was diluted with citric acid (5% w/v) and extracted with DCM (3x). The combined organic phases were dried over Na_2SO_4, filtered and the solvent was removed *in vacuo*, then coevaporated with toluene (1x) and Et_2O (1x). The resulting orange solid was used for the next step with no further purification. The orange solid was dissolved in THF (525 mL), NaH_2PO_4*$2H_2O$ (18.5 g, 119 mmol, 10 eq.) dissolved in water (240 mL) and NH_4OH solution (20% w/v, 6.80 mL, 35.7 mmol, 3.0 eq.) was added. The solution was degassed by bubbling N_2 threw the solution. To the mixture, $Pd(OH)_2/C$ (20% loaded, 540 mg) was added and the mixture was stirred under H_2-atmopshere at rt for 2d. To the mixture, $NaHCO_3$ (5.00 g, 59.5 mmol, 5 eq.) was added and the pH was checked to be neutral. To this Fmoc-Osu (3.30 g, 9.76 mmol, 0.82 eq., calculated based on HPLC-purity at 300 nm) dissolved in THF (50 mL) was added

and the mixture stirred at rt for 4 h. The mixture was filtered threw celite, washed with THF (ca. 150 mL), concentrated to ca. 250 mL, diluted with H_2O to 500 mL and acidified to pH = 3 with citric acid (5% w/v). The mixture was extracted with DCM (3x), the combined organic phases dried over $MgSO_4$, filtered and the solvent was removed *in vacuo* and coevaporated with MeCN to give a dark green oil. The oil was dissolved in MeCN (15 mL) and precipitated under sonication. The compound was isolated by filtration and washed with MeCN and then further purified by column chromatography (silica, 1-3% MeOH/DCM + 0.1% AcOH) to give the title compound as a white solid (2.22 g, 4.47 mmol, 38%).

NMR does show 2 conformers ratio 85:15. Integrals are given with their respective integration. Overlapping NMRs are integrated as m and their integration is given.

^{1}H NMR (500 MHz, DMSO-*d*$_6$) δ [ppm] = 8.14 (dd, J = 8.5, 1.4 Hz, 1H), 8.01 (s, 1H), 7.91 (dd, J = 11.2, 6.8 Hz, 2.6H), 7.79 (d, J = 7.6 Hz, 0.3H), 7.73 – 7.67 (m, 2.6H), 7.64 (d, J = 7.8 Hz, 0.15H), 7.60 (dd, J = 7.0, 1.3 Hz, 0.85H), 7.53 (t, J = 6.5 Hz, 0.15H), 7.42 (t, J = 7.5 Hz, 1.8H), 7.35 (d, J = 7.6 Hz, 0.3H), 7.31 (td, J = 7.5, 1.2 Hz, 1.8H), 7.04 (t, J = 7.5 Hz, 0.3H), 4.87 (d, J = 6.2 Hz, 1.7H), 4.79 (d, J = 6.4 Hz, 0.3H), 4.44 (bs, 1H), 4.40 (d, J = 6.8 Hz, 1.7H), 4.30 (d, J = 6.6 Hz, 0.15H), 4.25 (t, J = 6.8 Hz, 0.85H), 4.13 (t, J = 6.8 Hz, 1.15H), 3.55 (m, J = 0.15H), 3.44 (t, J = 6.4 Hz, 2H), 3.17 (t, J = 7.7 Hz, 2H), 3.06 (q, J = 7.3 Hz, 0.15H), 1.80 – 1.69 (m, 2H), 1.57 – 1.49 (m, 2H), 1.25 – 1.19 (m, 1H).

^{13}C NMR (126 MHz, DMSO-d$_6$) δ [ppm] = 166.4, 156.6, 150.8, 147.02, 144.6, 143.9, 140.8, 138.7, 128.1, 128.0, 127.6, 127.5, 127.1, 125.2, 122.9, 120.2, 120.0, 65.3, 60.4, 46.9, 40.7, 32.2, 31.4, 26.5.

HRMS: (ESI$^+$) m/z calc. for $C_{30}H_{29}N_2O_5$: 497.2071 $[M+H]^+$; found: 497.2073.

Compound **11**

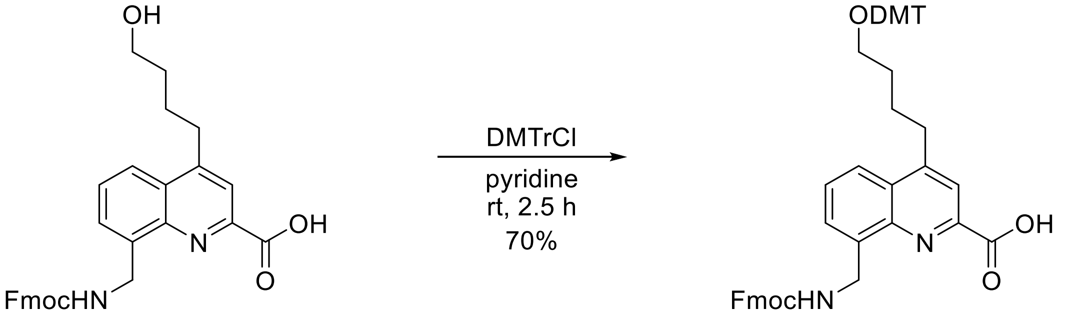

10 (295 mg, 594 µmol, 1.0 eq.) and DMTrCl (262 mg, 772 µmol, 1.3 eq.) were dissolved in anhydrous pyridine (1.8 mL) under N_2-atmosphere and stirred at rt for 3h. The solvent was removed *in vacuo.* The product was dissolved in DCM (50 mL) and the organic phase was washed with water (3x50 mL) and the combined aqueous phases were back extracted with DCM (30 mL). The combined organic phases were dried over $MgSO_4$, filtered and the solvent was removed in vacuo. The crude product was purified by automated reversed-phase column chromatography (C18, 50 → 100% MeCN in H_2O) to give the title compound (332 mg, 416 µmol, 70%) as a white solid.

NMR does show 2 conformers ratio 85:15. Integrals are given with their respective integration. Overlapping NMRs are integrated as m and their integration is given.

^{1}H NMR (500 MHz, DMSO-d$_6$) δ [ppm] = 13.10 (s, 1H), 8.08 (dd, *J* = 8.4, 1.6 Hz, 1H), 7.99 (s, 1H), 7.89 (d, *J* = 7.2 Hz, 2.5H), 7.75 (d, *J* = 7.7 Hz, 0.3H), 7.69 (d, *J* = 7.5 Hz, 1.7H), 7.67 – 7.57 (m, 1.7H), 7.59 – 7.50 (m, 0.3H), 7.41 (t, *J* = 7.5 Hz, 1.7fH), 7.39 – 7.32 (m, 2H), 7.34 – 7.26 (m, 4H), 7.25 – 7.21 (m,

5H), 7.21 – 7.19 (m, 0.5fH), 7.01 (t, *J* = 7.5 Hz, 0.3H), 6.91 – 6.83 (m, 4H), 4.87 (d, *J* = 6.2 Hz, 1.7H), 4.78 (d, *J* = 6.4 Hz, 0.3H), 4.40 (d, *J* = 6.8 Hz, 1.7H), 4.30 (d, *J* = 6.3 Hz, 0.3H), 4.25 (t, *J* = 6.7 Hz, 0.85H), 4.11 (t, *J* = 6.3 Hz, 0.15H), 3.71 (s, 6H), 3.10 (t, *J* = 7.7 Hz, 2H), 3.01 (t, *J* = 6.1 Hz, 2H), 1.78 (q, *J* = 8.0 Hz, 2H), 1.68 (q, *J* = 6.8 Hz, 2H).

^{13}C NMR (126 MHz, DMSO-d$_6$) δ [ppm] = 166.19, 157.97, 156.54, 150.64, 146.69, 145.19, 144.56, 143.87, 143.85, 140.78, 138.70, 135.96, 129.55, 128.04, 127.97, 127.79, 127.60, 127.58, 127.01, 126.56, 125.12, 122.79, 120.12, 119.90, 113.13, 85.21, 65.26, 62.18, 54.99, 46.85, 40.66, 31.23, 29.03, 26.68.

HRMS: (ESI$^+$) m/z calc. for $C_{51}H_{20}N_2O_7$: 799.3378 [M+H]$^+$; found: 799.3388.

3.1.3 Linker assembly & activation

Compound **12**

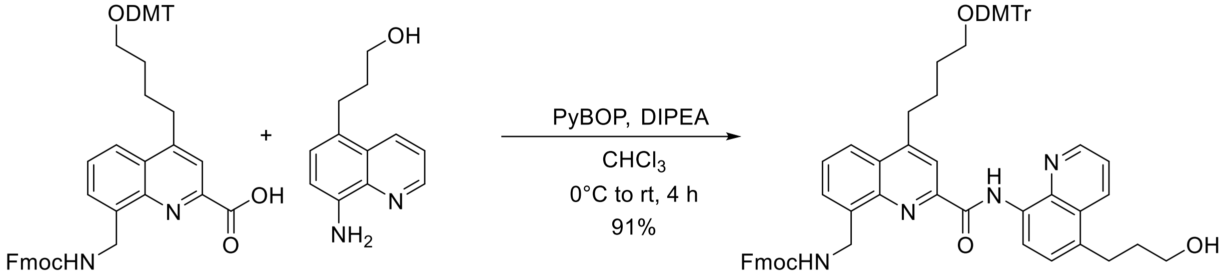

11 (315 mg, 394 µmol, 1.0 eq.) and PyBOP (410 mg, 789 µmol, 2.0 eq.) were dissolved in anhydrous chloroform (8.00 mL) under N_2-atmosphere and the solution was cooled to 0°C. **5** (87.7 mg, 0.43 mmol, 1.0 eq.) was dissolved in anhydrous chloroform and added to the reaction mixture. DIPEA was added and reaction mixture was stirred at rt for 4h. The solvent was removed *in vacuo* and the crude product was purified by column chromatography (60% EtOAc in cHex + 0.1% TEA) to give the title compound (351 mg, 357 µmol, 91%) as a pale yellow solid.

NMR shows 2 conformers ratio 85:15. Only major species in ^{13}C NMR listed.

^{1}H NMR (500 MHz, DMSO-d$_6$) δ [ppm] = 12.68 (s, 0.85H), 12.66 (s, 0.15H), 8.94 (d, *J* = 3.2 Hz, 2H), 8.73 (d, *J* = 7.8 Hz, 1H), 8.60 (d, *J* = 8.4 Hz, 1H), 8.18 (s, 1H), 8.11 (d, *J* = 6.3 Hz, 1H), 7.89 (t, *J* = 5.9 Hz, 0.85H), 7.86 (d, *J* = 7.6 Hz, 1.70H), 7.72 – 7.61 (m, 4.85H), 7.52 (d, *J* = 7.9 Hz, 1H), 7.47 – 7.43 (m, 0.30H), 7.41 – 7.34 (m, 3.80H), 7.29 (t, *J* = 7.6 Hz, 2.30H), 7.26 – 7.18 (m, 7H), 7.13 (t, *J* = 7.6 Hz, 0.30H), 6.87 (d, *J* = 8.8 Hz, 4H), 5.15 (d, *J* = 5.7 Hz, 1.70H), 5.06 – 5.01 (m, 0.30H), 4.58 (t, *J* = 5.1 Hz, 1H), 4.42 (d, *J* = 6.8 Hz, 1.70H), 4.35 (d, *J* = 6.1 Hz, 0.30H), 4.22 (t, *J* = 6.8 Hz, 0.85H), 4.07 (s, 0.15H), 3.71 (s, 6H), 3.51 (q, *J* = 5.8 Hz, 2H), 3.16 (t, *J* = 7.7 Hz, 2H), 3.08 (t, *J* = 7.8 Hz, 2H), 3.03 (t, *J* = 6.2 Hz, 2H), 1.88 – 1.78 (m, 4H), 1.72 – 1.66 (m, 2H).

^{13}C NMR (126 MHz, DMSO-d$_6$) δ [ppm] = 161.63, 157.97, 156.51, 151.58, 148.73, 147.86, 145.19, 143.84, 143.81, 143.70, 140.76, 138.71, 137.93, 135.97, 133.46, 133.26, 132.02, 129.56, 128.91, 128.04, 127.90, 127.79, 127.61, 127.54, 127.28, 126.93, 126.57, 126.52, 126.50, 125.11, 122.92, 122.07, 121.38, 120.06, 120.02, 119.80, 117.53, 115.20, 113.13, 109.76, 85.22, 65.30, 62.21, 60.16, 54.98, 46.86, 40.78, 34.03, 31.43, 29.07, 27.74, 26.71.

HRMS: (ESI$^+$) m/z calc. for $C_{63}H_{59}N_4O_7$: 983.4378 [M+H]$^+$; found: 983.4388.

Compound **1**

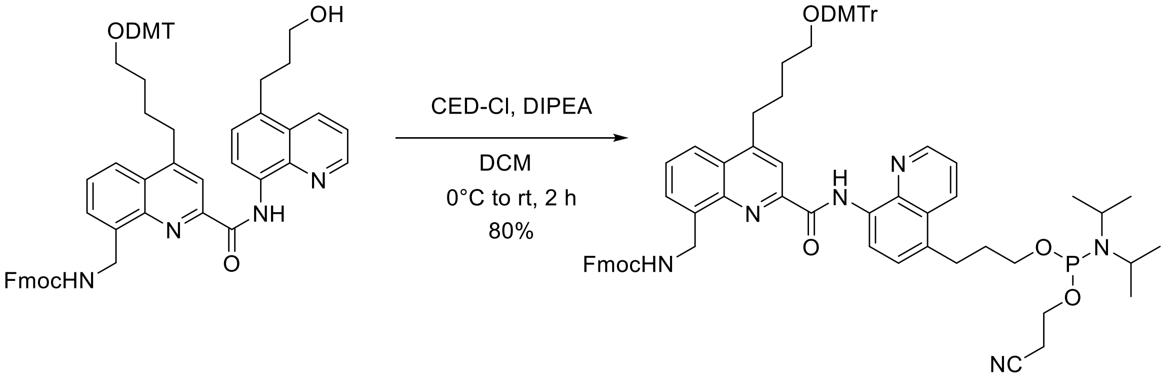

11 (439 mg, 447 mmol, 1.0 eq.) was dissolved in dry degassed DCM (4.5 mL) under argon atmosphere. DIPEA (311 mL, 1.79 mmol, 4.0 eq.) and CED-Cl (249 mL, 1.12 mmol, 2.5 eq.) were added at 0 °C and the reaction mixture was stirred for 2h. Subsequently the mixture was diluted with DCM (15 mL) and quenched with sat. sodium bicarbonate solution (10 mL). The aqueous phase was extracted with DCM (3×10 mL), the organic layers were combined, washed with brine (5 mL) and dried over Mg_2SO_4. The solvent was removed *in vacuo* and the product was purified by flash column chromatography (silica, EtOAc/nHex= 1:2) to yield **1** (421 mg 356 mmol, 80%) as a yellow powder after lyophilization from benzene (3×). $\mathbf{R}_{\boldsymbol{f}}$ = 0.65 (EtOAc/nHex = 1:2)

^{31}**P NMR** (202 MHz, Acetone-d_6): δ(ppm) = 152.34, 143.69.
HRMS: (ESI$^+$) m/z calc. for $C_{72}H_{76}N_6O_8P$: 1183.5462 $[M+H]^+$; found: 1183.5322.

3.2 Foldamer synthesis

3.2.1 General solid-phase foldamer synthesis

Solid phase synthesis (SPS) was performed manually under MW-irradiation on a CEM Discover (Liberty Bio) microwave oven using a reaction vessel and an internal fiber optic probe for temperature control, or with a fully automated synthesizer followed by previously reported protocol.[2, 64]

- Bromination of low loading Wang resin

Low loading-(LL) Wang resin (Novabiochem, 100-200 mesh, 1.00 g, 0.37 mmol, 1.0 eq.) was swollen in DMF (6 mL) for 30 min. PPh_3 (970 mg, 3.70 mmol, 10.0 eq.) and CBr_4 (1.23 g, 3.70 mmol, 10 eq.) were quickly added and the suspension was stirred slowly for 20 h at RT. The resin was filtered, washed with DMF (3 × 3 mL) and DCM (3 × 3 mL), and dried by passing N_2 through the resin and stored at 4 °C until usage.

- Loading of the 1st monomer unit & resin loading estimation

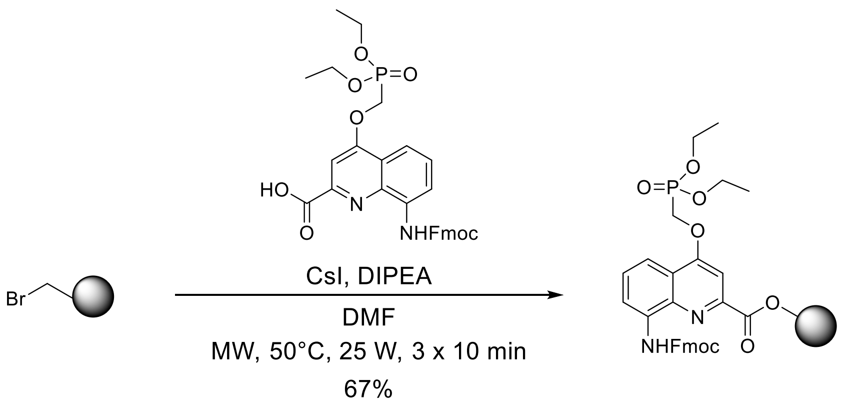

LL-brominated Wang resin (Novabiochem, 100-200 mesh, 100 mg, 37.0 µmol, 1.0 eq.) was swollen in anhydrous DMF (3 mL) for 30 min under N_2. After washing with anhydrous DMF (3 mL), Fmoc-Q_4-COOH (42.5 mg, 74.0 µmol, 2.0 eq.) dissolved in anhydrous DMF (2 mL), CsI (19.2 mg, 74.0 µmol, 2.0 eq.) and freshly distilled DIPEA (9.56 mg, 12.9 µL, 74 µmol, 2.0 eq.) were quickly added and the reaction vessel was placed under microwave irradiation (25 W, ramp to 50°C over 5 min, hold at 50°C for 10 min) while bubbling N_2 through the solution. The resin was washed with anhydrous DMF (3 × 3 mL) and the process was repeated twice. The resin was washed with DMF (3 × 3 mL) and DCM (3 × 3 mL) and dried by passing N_2 through the resin. For resin loading estimation, a solution of 20% piperidine in DMF (v/v, 3 mL) was added to a known mass of the previously dried resin (1-2 mg) and agitated for 5 min. Meanwhile, the absorbance at 290 nm of the piperidine/DMF-solution was measured. After agitation, the absorbance of the solution that contained the resin was measured at 290 nm.

$$Resin\ loading\ \left[\frac{mmol}{g}\right] = \frac{[Abs_{final} - Abs_{initial}]}{[2.00 \times m_{resin}]}$$

- Fmoc deprotection

To the pre-swollen loaded Wang resin (75.0 mg, 0.26 mmol g^{-1}, 19.5 µmol), a 20% solution of piperidine in DMF (3 mL, v/v) was added and the resin was mixed by bubbling N_2-gas through the solution for 3 min. The resin was filtered and washed with DMF (2 x 3 mL) and the deprotection was repeated once for 7 min to give the respective amine NH_2-Q-Wang resin. The resin was filtered, washed with DMF (5 x 3 mL), and washed with anhydrous THF (3 x 3 mL) prior to coupling.

- *In situ* acid chloride activation, coupling, and capping

The NH_2-Wang resin (75.0 mg, 0.26 mmol g^{-1}, 19.5 µmol, 1.0 eq.) was suspended in anhydrous THF (0.9 mmol) and *2-4-6*-collidine (23.0 µL, 176 µmol, 9.0 eq.) was added. Concurrently, in a glass vial, **Monomer** (35 mg, 58.6 µmol, 3.0 eq.) and PPh_3 (41 mg, 156 µmol, 8 eq.) were mixed and dissolved in anhydrous $CHCl_3$ (0.9 mL). Subsequently, trichloroacetonitrile (18.0 µL, 176 µmol, 9 eq.) was added to the vial, which was quickly shaken and the mixture was added to the pre-swollen resin. After mixing, the reaction vessel was placed under microwave irradiation (25 W, ramp to 50°C over 5 min, hold at 50°C for 15 min). The resin was filtered off and washed with anhydrous THF (3 x 3 mL). The coupling step was repeated once. The resin was filtered off and washed with anhydrous THF (3 x 3 mL) and DCM (3 x 3 mL) prior to the capping step. The resin was suspended in a 50% solution of Ac_2O in DCM (v/v) and mixed by bubbling N_2-gas through the solution for 10 min. The resin was washed with DCM (2 x 3 mL) and DMF (2 x 3 mL).

- Resin cleavage

The resin-bound foldamer was placed in a syringe equipped with a filter and then suspended in TFA (3 mL). The resin was shaken for 2 h at RT. The resin was then filtered off and washed twice with TFA. TFA was removed *in vacuo* and the resulting solid was precipitated in cold Et_2O. The precipitate was centrifuged and the solvent was decanted to give a yellow solid. The decanted Et_2O was concentrated by rotary evaporation and the precipitation was repeated. The combined precipitates were dissolved in water/MeCN and then lyophilized to give the crude protected foldamer as a yellow solid and further purified as a diethyl-phosphonate protected compound.

3.2.2 Removal of the diethyl-phosphonate protecting groups

Purified protected foldamers were dissolved in anhydrous chloroform (1 mL per 10 mg compound) and cooled to 0 °C. TMSBr (0.2 mL per 10 mg compound) was diluted in anhydrous chloroform (0.8 mL per 10 mg compound). Diluted TMSBr-solution was added dropwise over 10 min to the reaction mixture. The reaction mixture was allowed to room temperature and stirred under N_2-atmosphere for 1-3 d until an HPLC- and LCMS aliquot of the reaction mixture showed full cleavage of all phosphonate-diethyl esters. The reaction mixture was evaporated *in vacuo* (40°C water bath) to give a yellow oil, then coevaporated with DCM (2x) to give a yellow solid. The solid was suspended in water, basified to pH > 12 with triethylamine and stirred for 30 min. The suspension was filtered through nylon syringe filters to give a pale yellow solution that was freeze-dried to give the crude deprotected foldamers as yellow solids.

3.2.3 Cation exchange chromatography

Cation exchange chromatography was performed on Dowex© 50WX4 200-400 (H) resin. The resin was swollen in H_2O the orange solution was decanted. The resin was transferred into a column and washed with H_2O (all with gravity flow) followed by two column volumes (CV) of 2 M HCl solution, washed with H_2O until pH = 6-7 (ca. 5-10 CV), washed with two CV of 2 M NH_4OAc solution, washed again with 5 CV of H_2O. Purified triethylammonium salts of foldamers were dissolved in water and loaded on the column, the column was closed without flow for 2 h, the column was eluted with water (20 mL) and the compound was lyophilized to give the purified foldamer as NH_4^+-salt as yellow solid.

3.2.4 Synthesized foldamers

3.2.4.1 Compound **22a** protected acetylated octamer

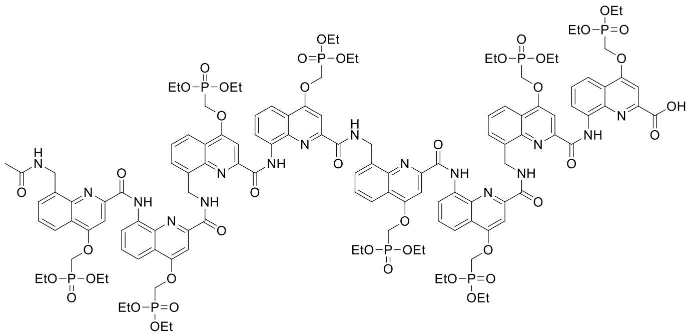

Oligomer **22a** was synthesized on LL-Wang resin (23.2 µmol). The compound was isolated by semi-preparative RP-HPLC (acidic conditions, linear gradient 30-100% B in A) to give the title compound (35.0 mg, 12.5 µmol, 54%) as a yellow solid.
(ESI$^+$) m/z calc. for $C_{126}H_{150}N_{16}O_{42}P_8$: 1403.399 [M+2H]$^{2+}$; found: 1403.393.

3.2.4.2 Compound **22** unprotected acetylated octamer

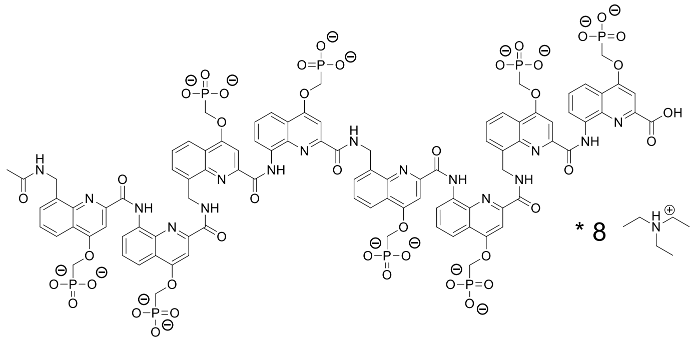

Compound **22a** (30.0 mg, 13.7 µmol, 1 eq.) was deprotected according to section 4.2.2 of the SI. Stirring time: 2 d at rt. The crude foldamer was purified by semi-preparative RP-HPLC (TEAA buffers, 0-50% B in A) to give the title compound (30.2 mg, 12.5 µmol, 89%) as a octa-triethylammonium salt as a pale yellow solid. (ESI$^-$) m/z calc. for $C_{94}H_{82}N_{16}O_{42}P_8$: 1177.1342 [M-2H]$^{2-}$; found: 1177.1368.

Synthesis of **23** is described here.[2]

3.3 Conjugate synthesis and oligonucleotides

3.3.1 DNA hairpin biotinylation

Isolated desalted DNA strand **20** (1.83 mg, 276 nmol, determined by UV-spectroscopy) were dissolved in degassed H_2O/DMF (250 µL, 1:4, v/v). Biotin-PEG4-NHS (5 eq.) ester was dissolved in degassed DMF (50 µL) and added to the reaction mixture. The reaction was followed by consumption of the starting material of an aliquot of the reaction by RP-HPLC (basic conditions) after 1 day. If incomplete, further Biotin-PEG_4-NHS (25 eq.) in DMF (100 µL) was added. Upon completion, the reaction mixture was diluted with degassed H_2O (15 mL) and lyophilized to give the crude biotinylated DNA-strand that was purified by basic HPLC (TEAA buffer system) to give the respective biotinylated DNA-strand **28** that was used as a triethylammonium salt for further usage (1.31 mg, 185 nmol, 67%). For **21** the reaction was performed on (520 µg, 78.2 nmol) to yield **29** (182 µg, 25.6 nmol, 33%).

3.3.2 By EDC hydrochloride

Desalted DNA-strand (2.28 mg, 548 nmol, 1eq.) was dissolved in water (500 µL). A polytriethyl ammonium salt of respective foldamer (4.35 mg, 1.25 µmol, 2.3 eq.) was dissolved in water (100 µL). EDC hydrochloride (3.15 mg, 16.4 µmol, 30 eq.), N-hydroxysuccinimide (1.89 mg, 16.4 µmol, 30 eq.) and DIPEA (2.86 µL, 16.4 µmol, 30 eq.) were dissolved in water (50 µL). EDC hydrochloride, NHS and DIPEA were added to dissolved foldamer. The activated foldamer solution was added to the DNA-strand and the mixture was shaken at 700 rpm at 21 °C for 24 h. The mixture was directly injected into semi-preparative RP-HPLC for purification (basic conditions, gradient 0-30% A in B, NH_4OAc buffer system) to obtain the foldamer-DNA conjugate as an NH_4^+-salt as pale yellow solid (2.01 mg, 307 nmol, 56%).

3.3.3 By DMTMM hydrochloride

Desalted DNA-strand (493 µg, 118 nmol, 1eq.) and polytriethyl ammoniumsalt foldamer (1.88 mg, 544 nmol, 4.6 eq.) were dissolved in water (90 µL) and diluted with MES-buffer (100 µL, 300 mM, pH = 6) to a volume of 200 µL. DMTMM hydrochloride (983 µg, 3.55 µmol, 30 eq.) was dissolved in water (10 µL) and added and the mixture was shaken at 700 rpm at 20 °C for 24 h. The mixture was directly injected into semi-preparative HPLC for purification (gradient 0-20% B in A, NH_4OAc buffer system) to obtain the foldamer-DNA conjugate as an NH_4^+-salt as pale yellow solid (338 µg, 49.8 nmol, 42%).

4 Spectra & Chromatogramms

4.1 NMR spectra

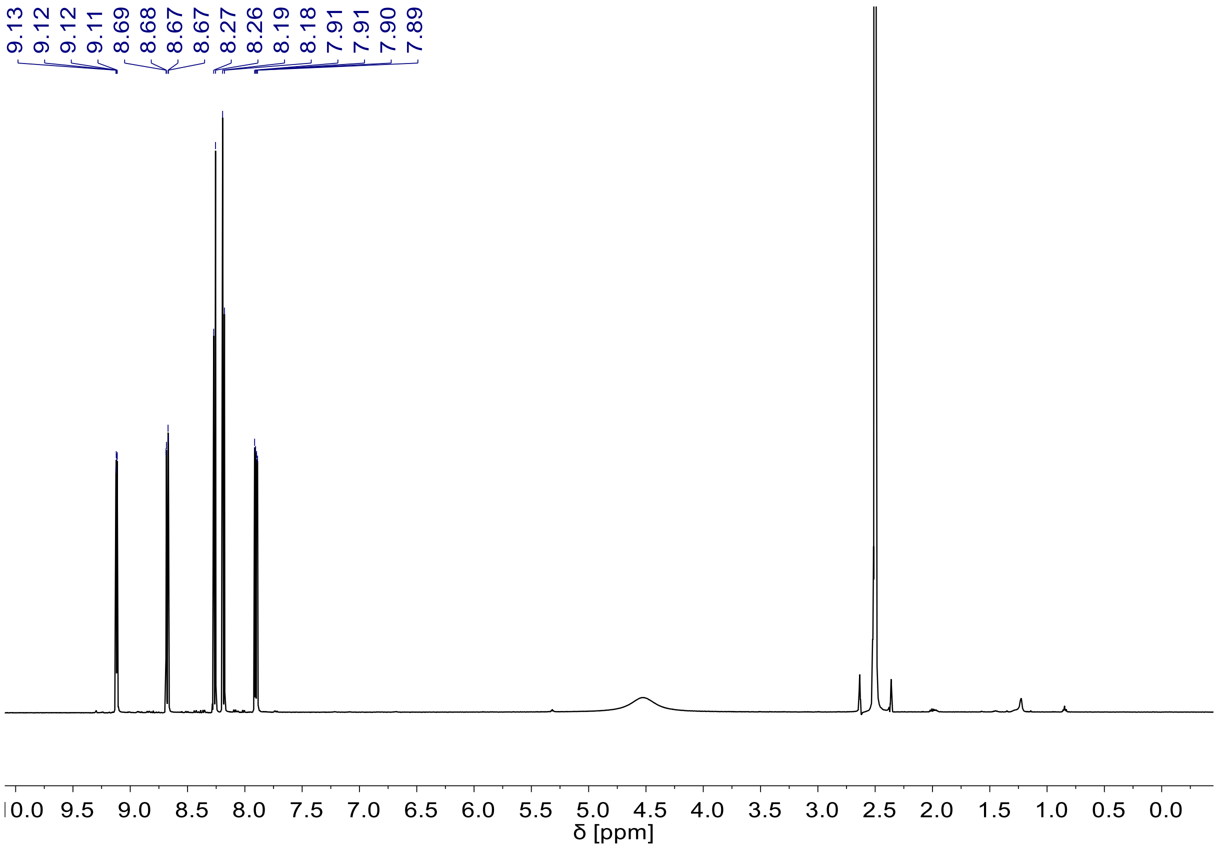

Figure S20 ^{1}H NMR spectrum of compound **3**.

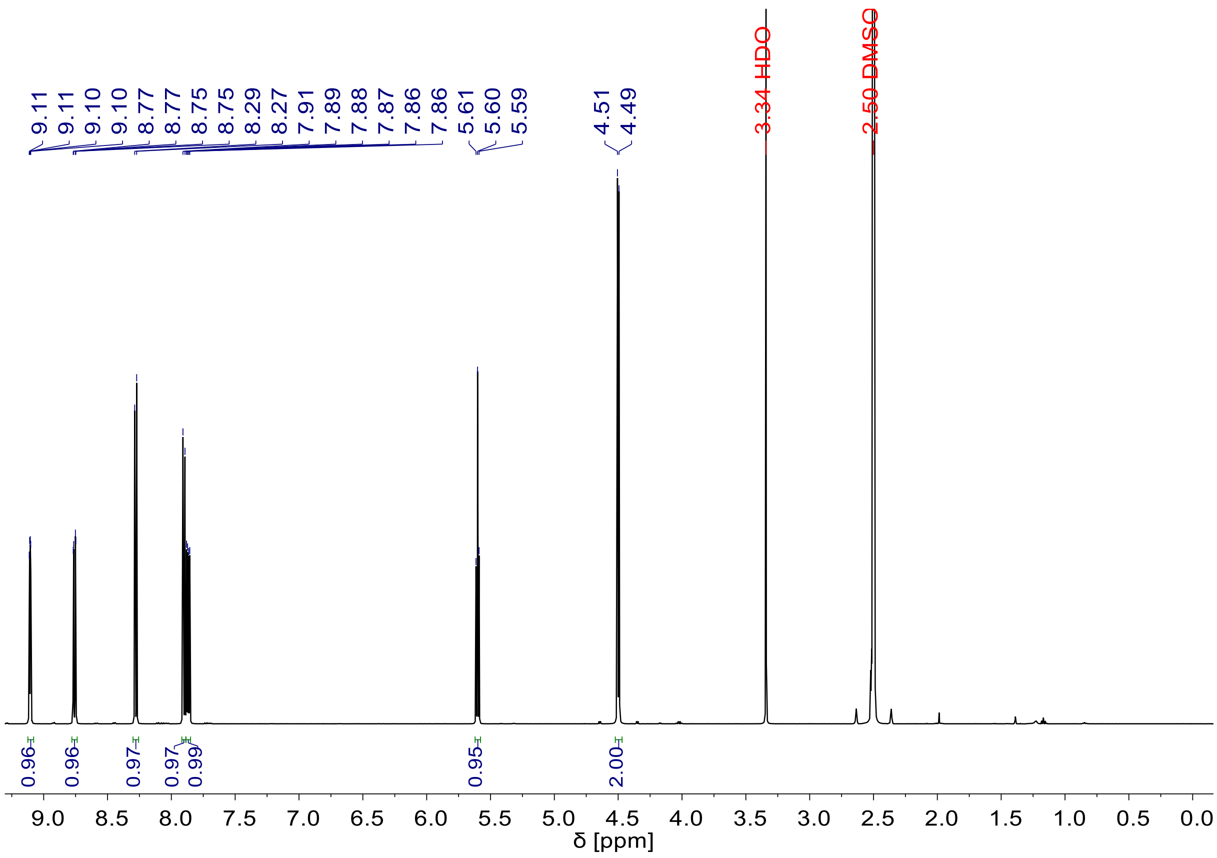

Figure S21 ^{1}H NMR spectrum of compound **4**.

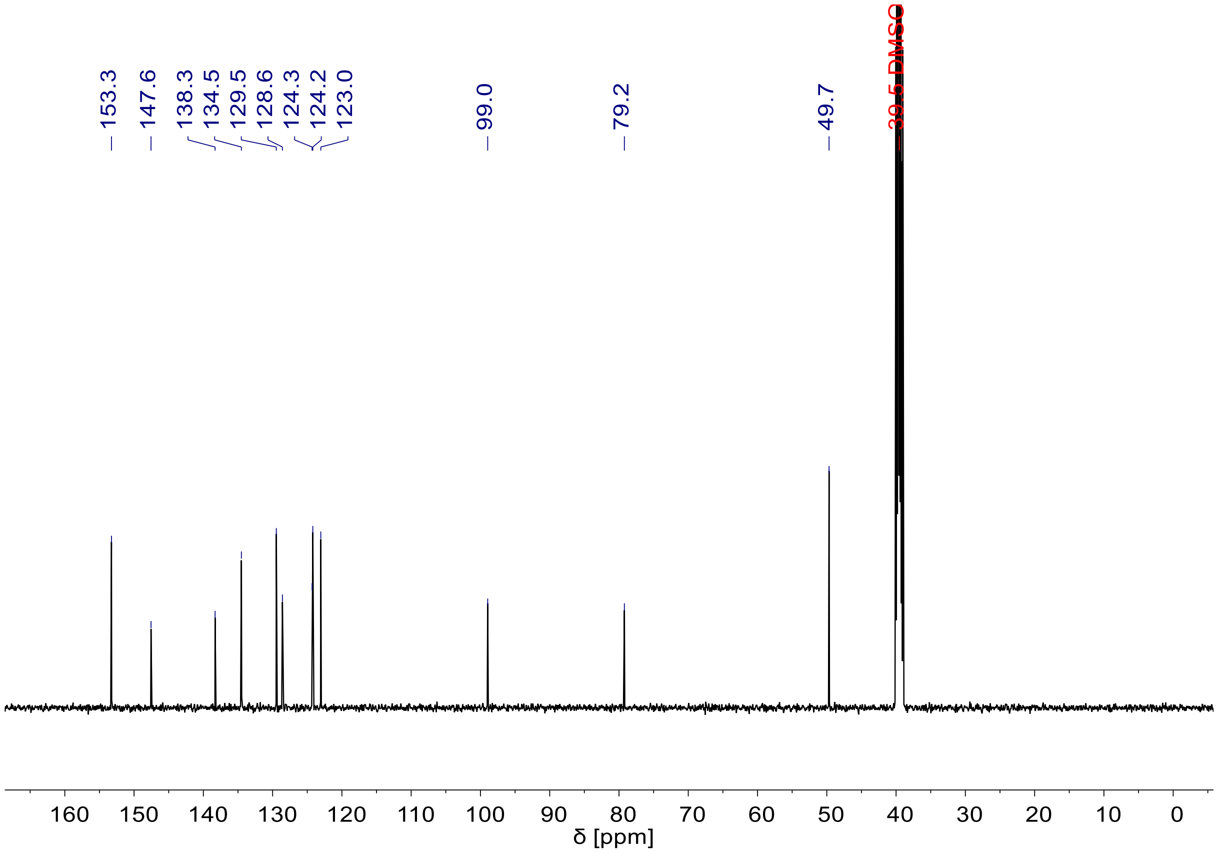

Figure S22 ^{13}C NMR spectrum of compound **4.**

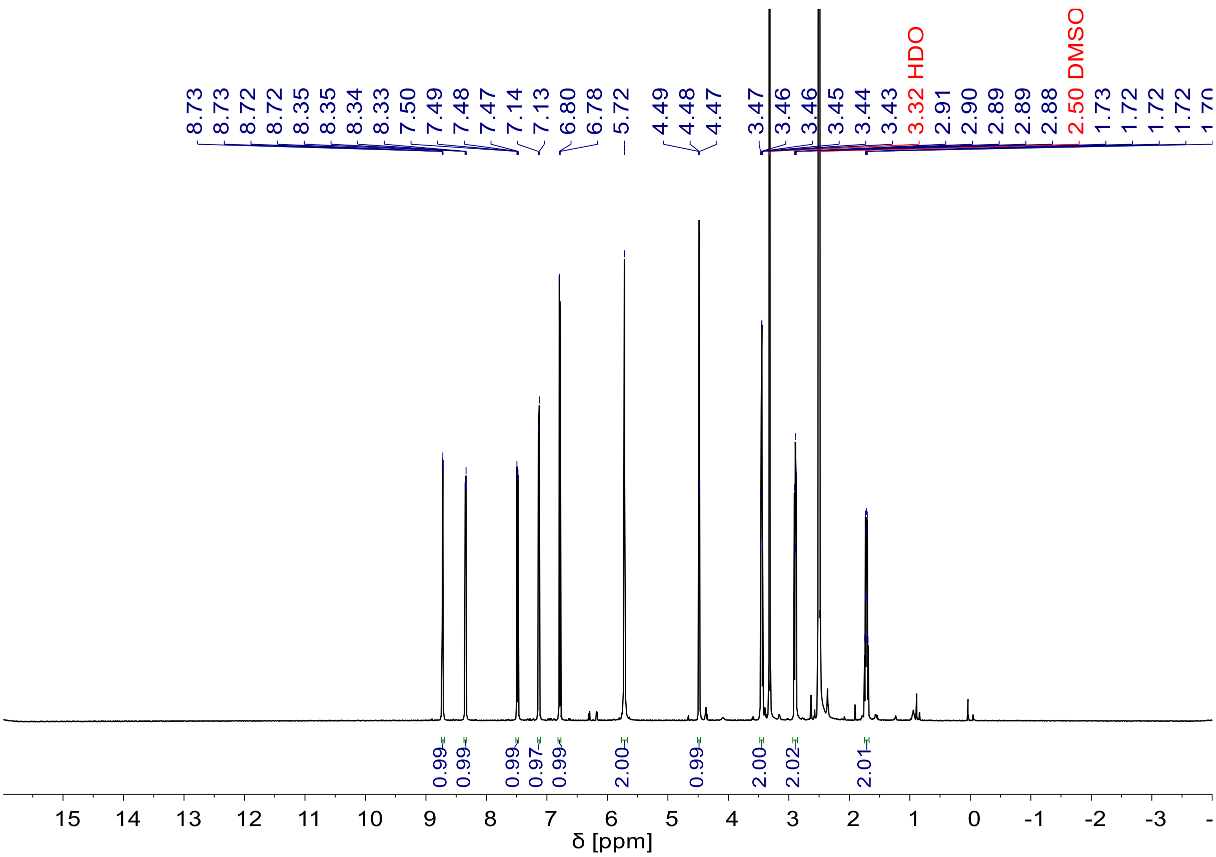

Figure S23 ^{1}H NMR spectrum of compound **5**.

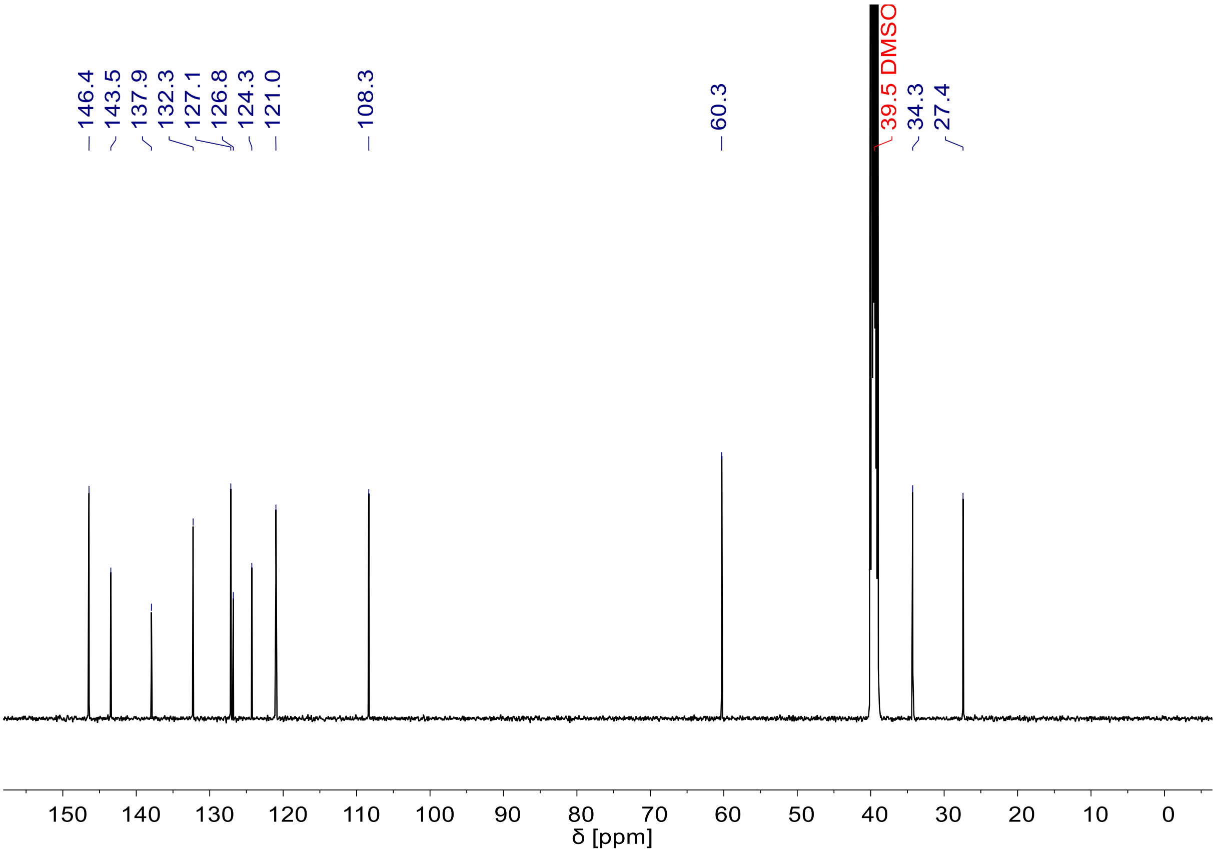

Figure S24 ^{13}C NMR spectrum of compound **5**.

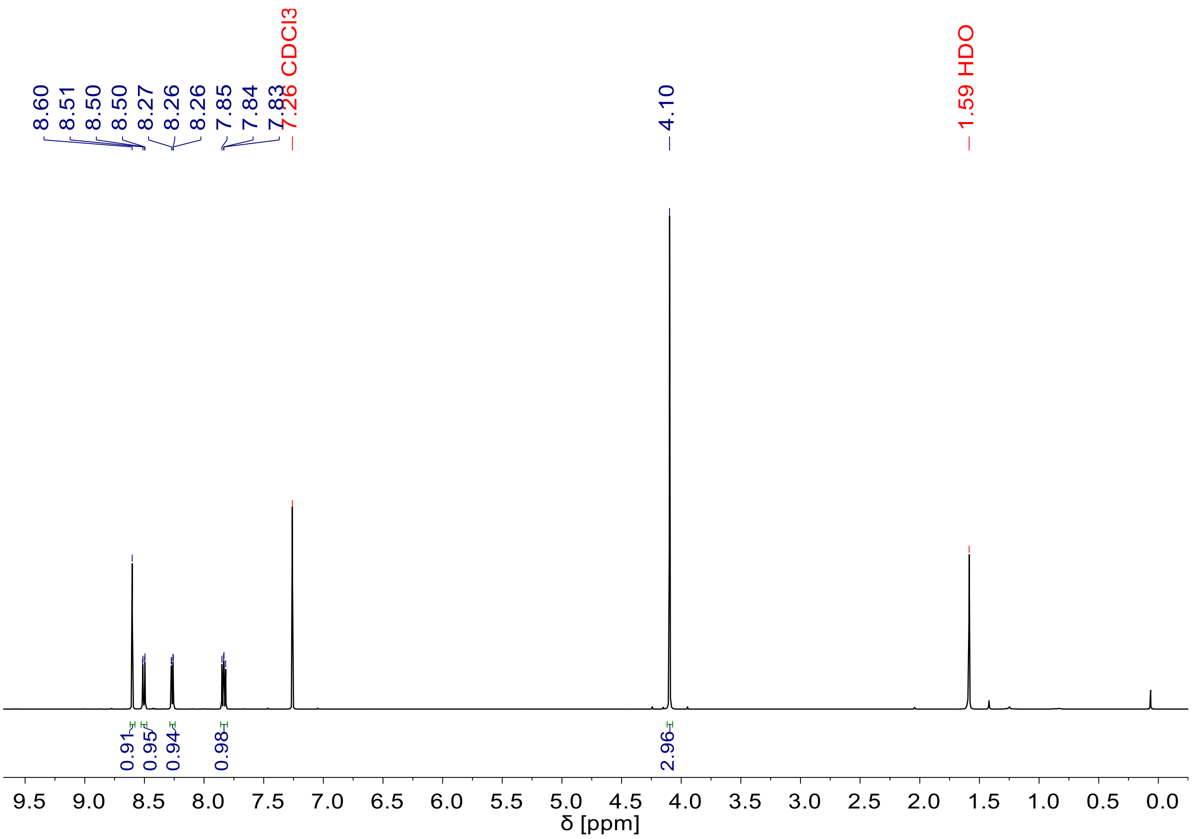

Figure S25 ^{1}H NMR spectrum of compound **7.**

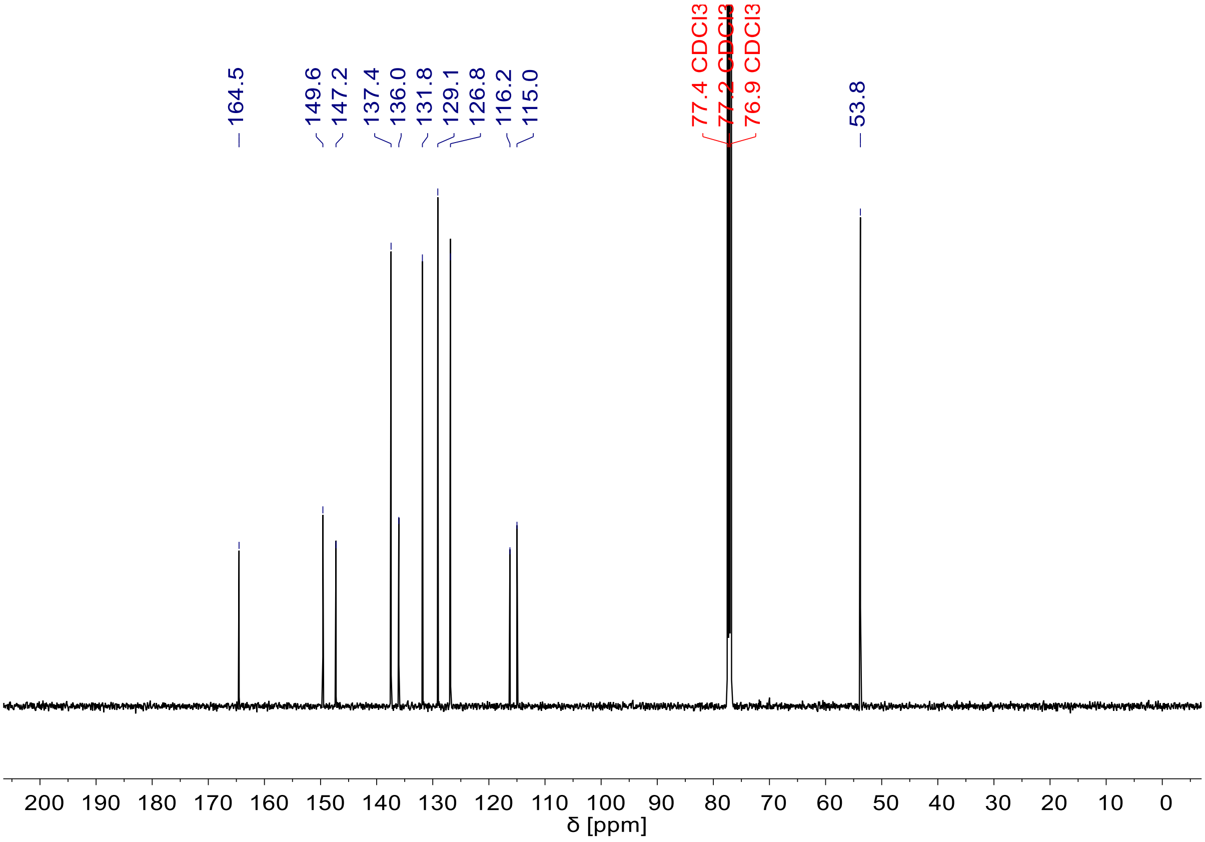

Figure S26 ^{13}C NMR spectrum of compound **7**.

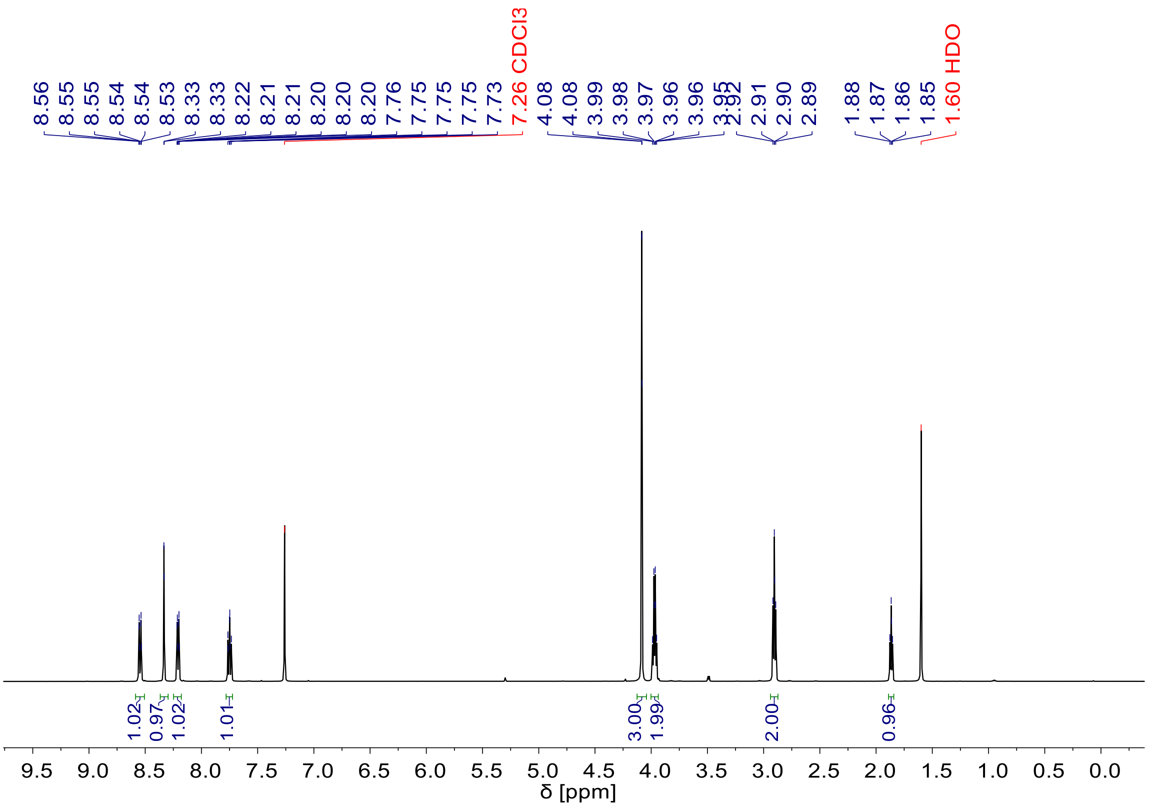

Figure S27^{1}H NMR spectrum of compound **8**.

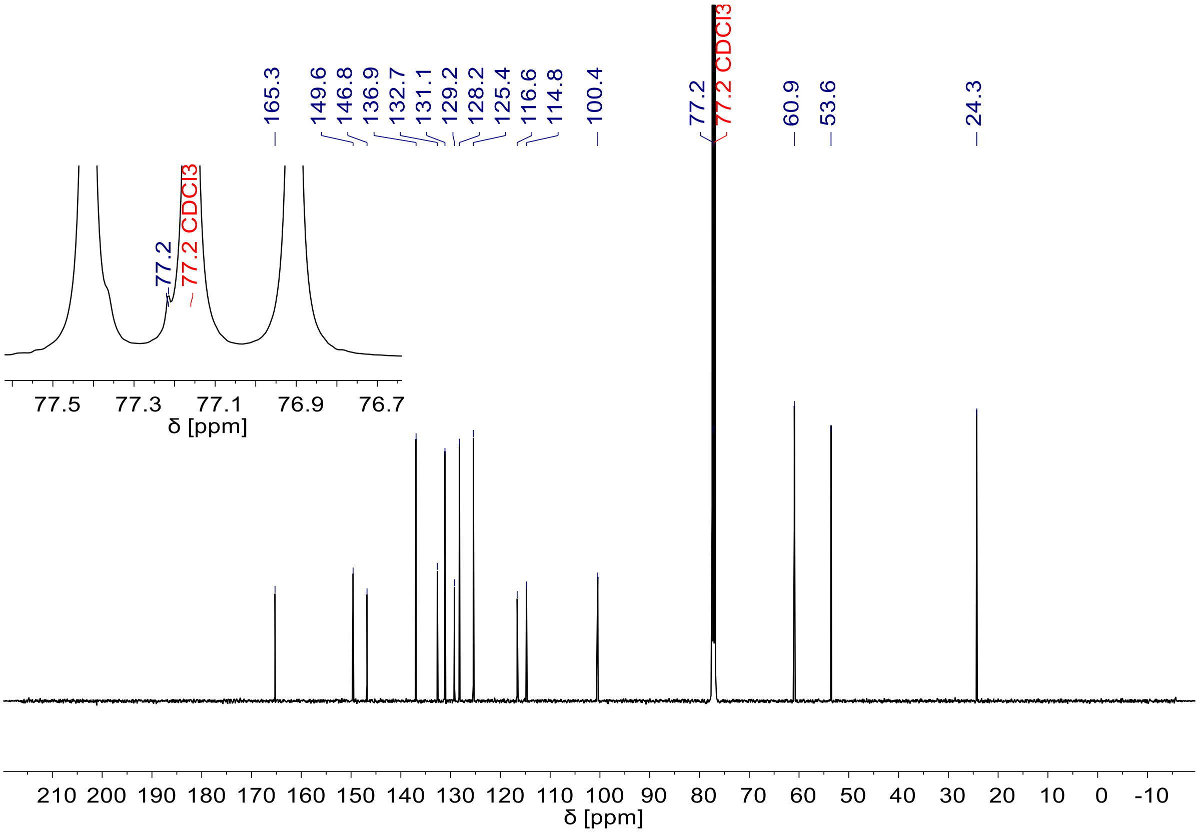

Figure S28 ^{13}C NMR spectrum of compound **8**.

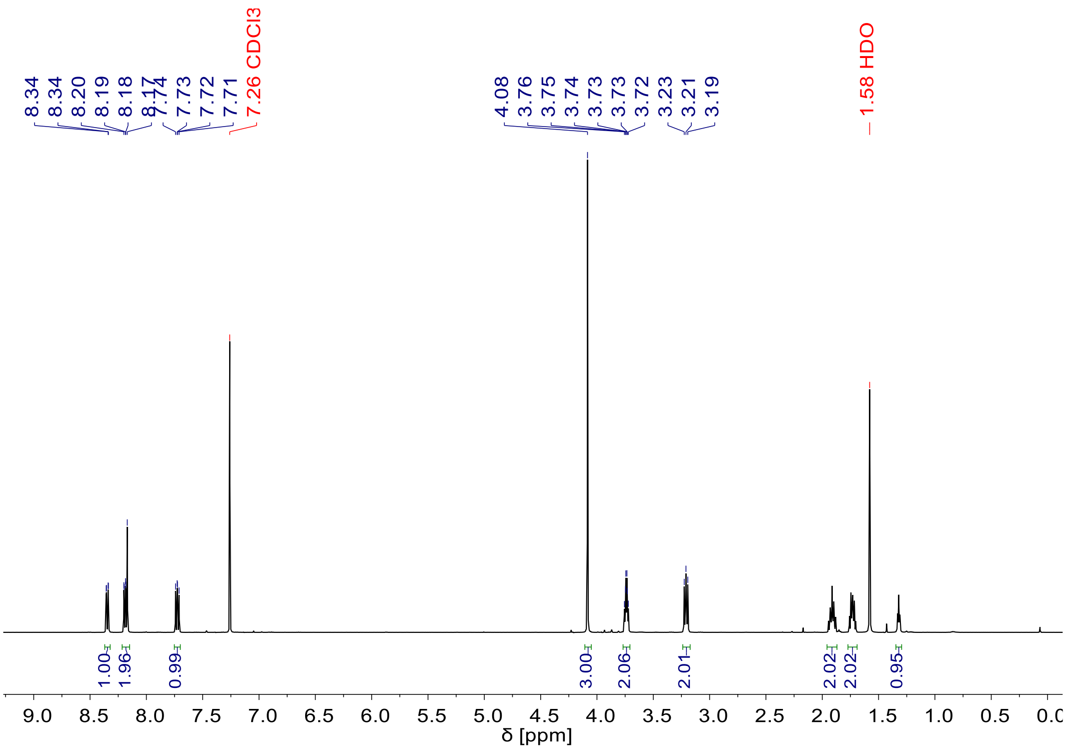

Figure S29 ^{1}H NMR spectrum of compound **9**.

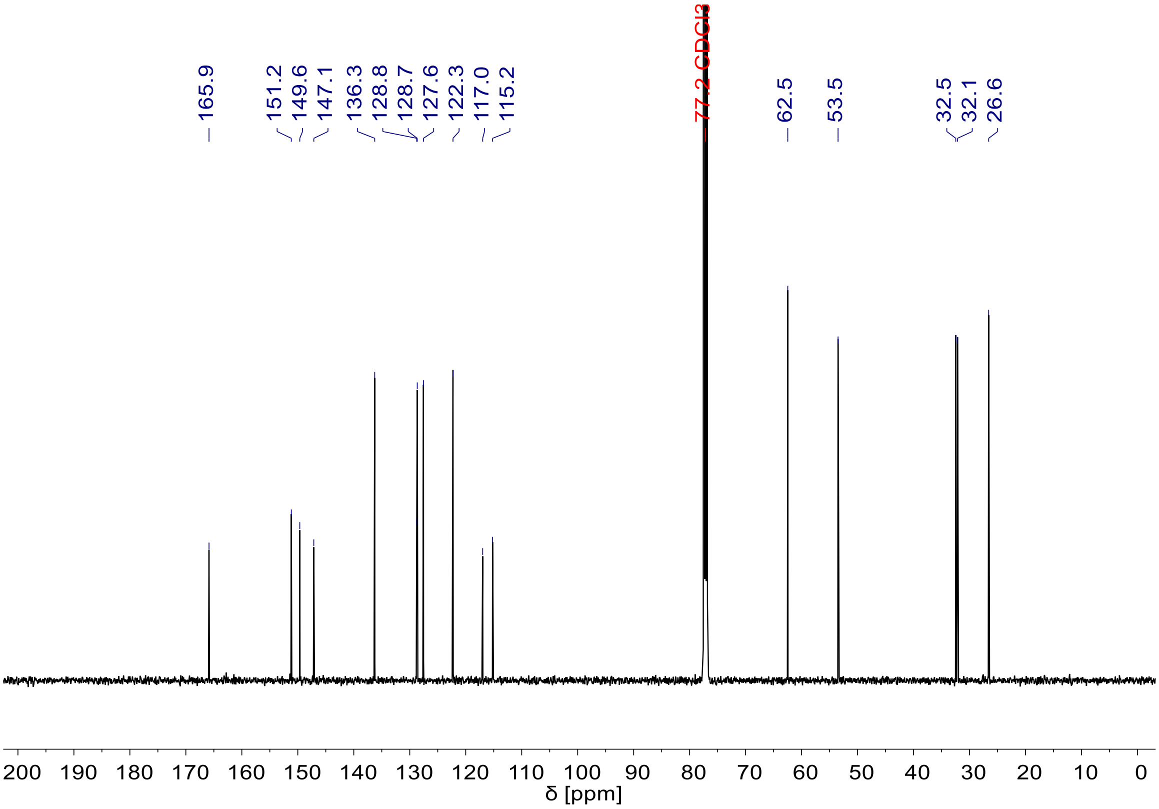

Figure S30 ^{13}C NMR spectrum of compound **9**.

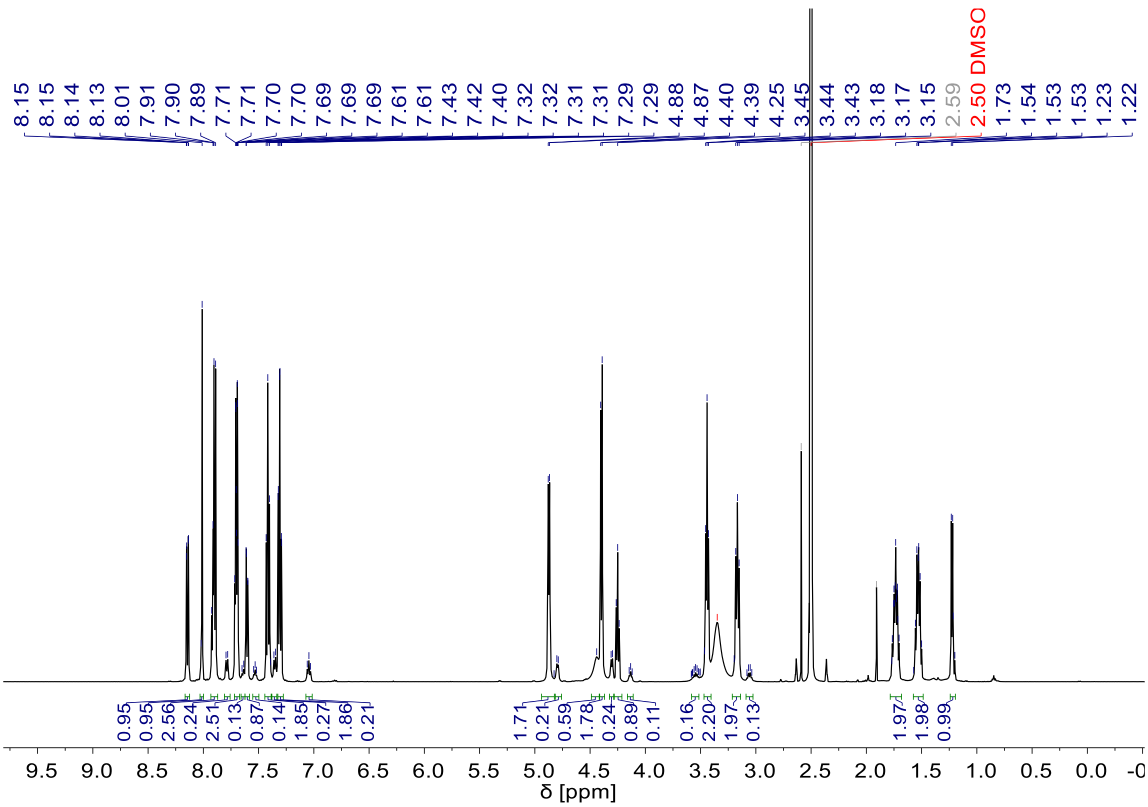

Figure S31 ^{1}H NMR spectrum of compound **10**.

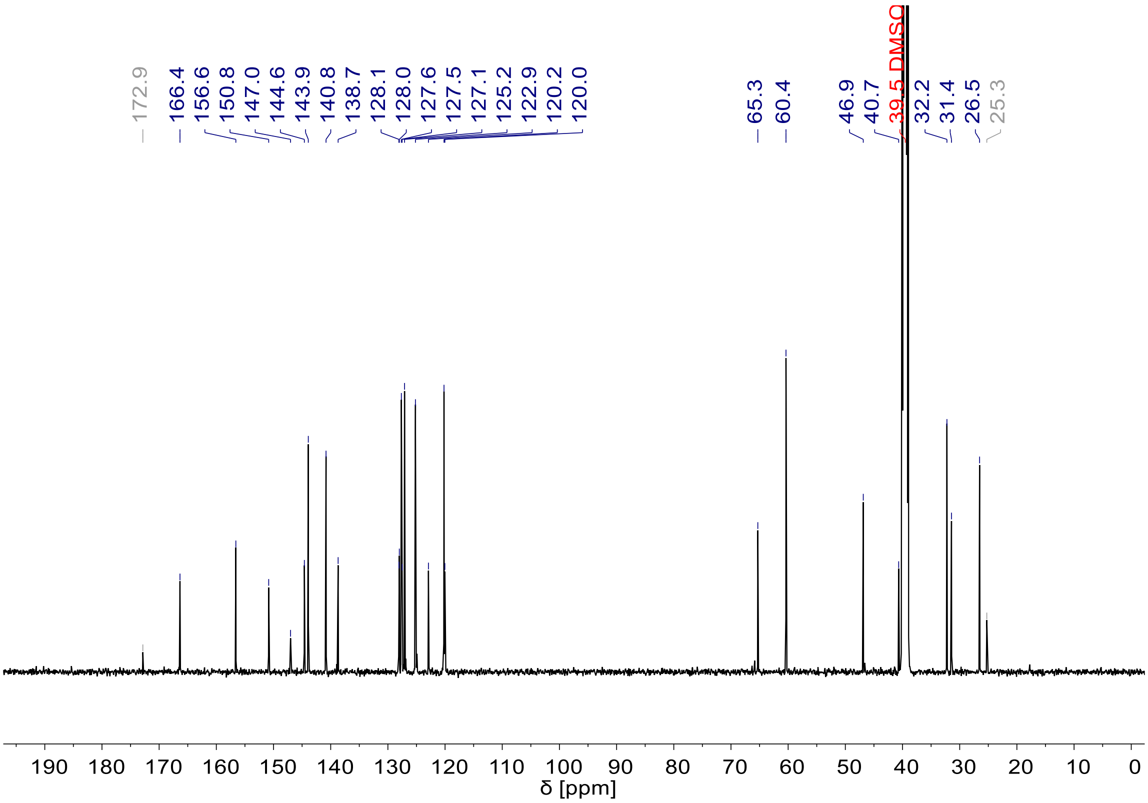

Figure S32 ^{13}C NMR spectrum of compound **10**.

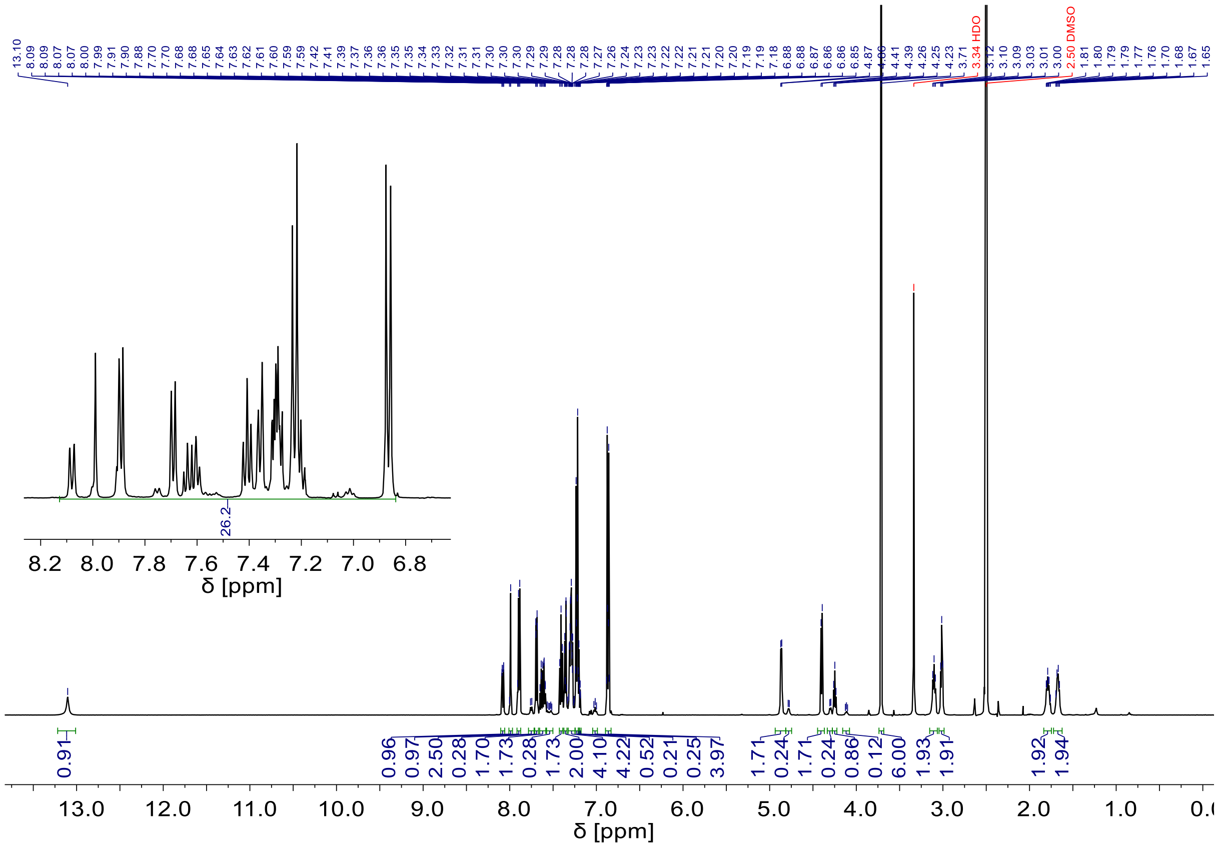

Figure S33 ^{1}H NMR spectrum of compound **11**.

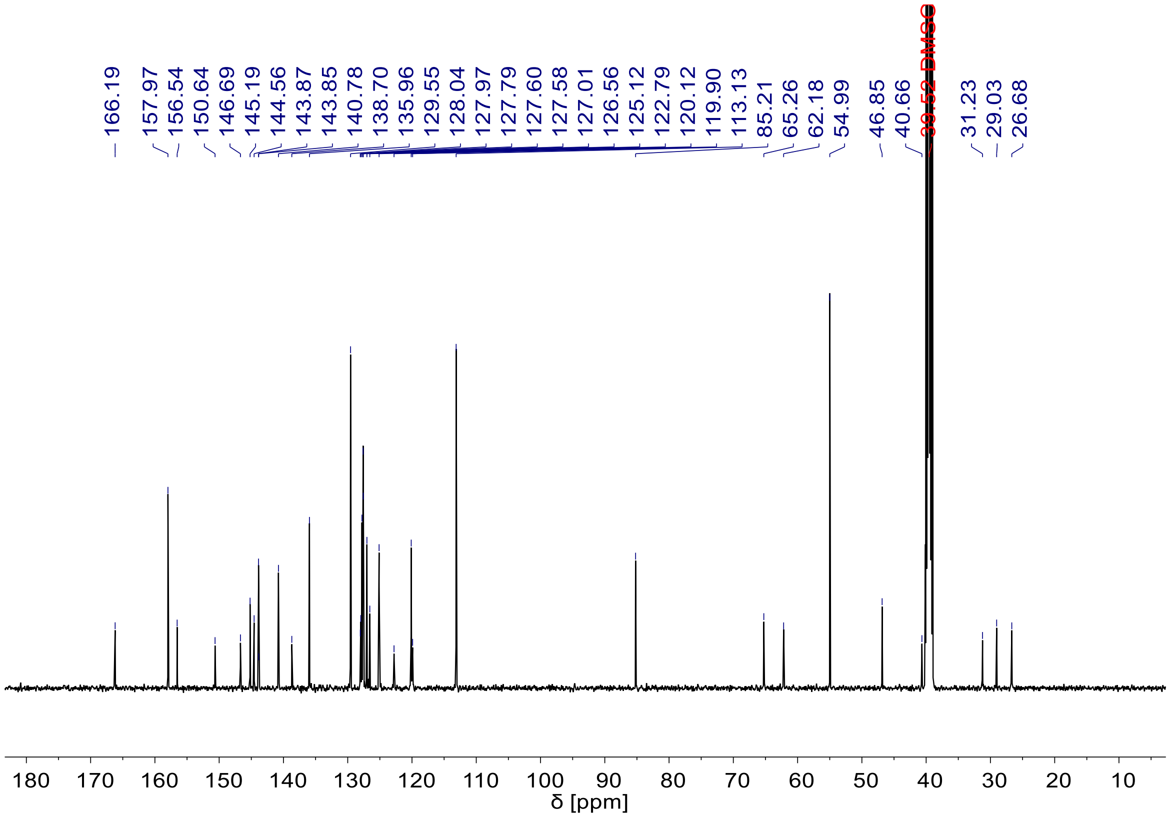

Figure S34 ^{13}C NMR spectrum of compound **11.**

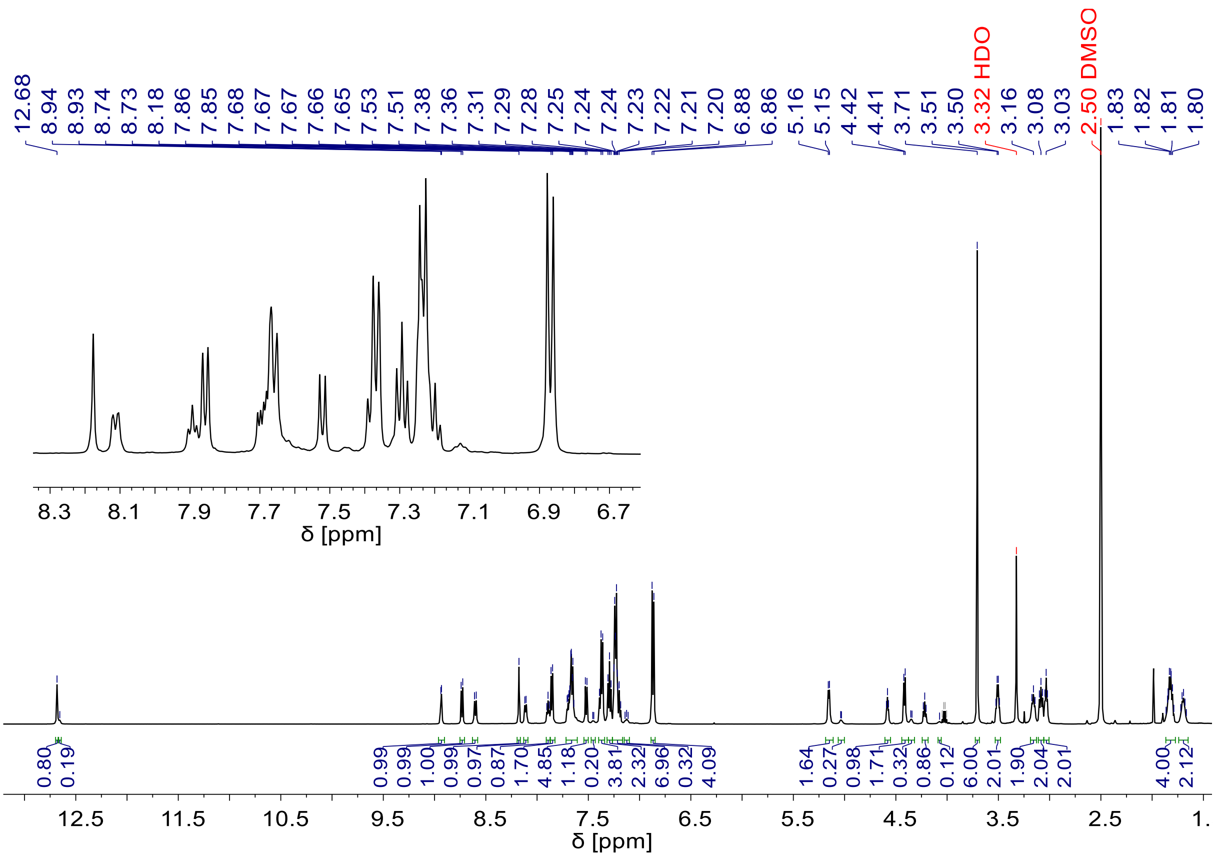

Figure S35 ^{1}H NMR spectrum of compound **12**.

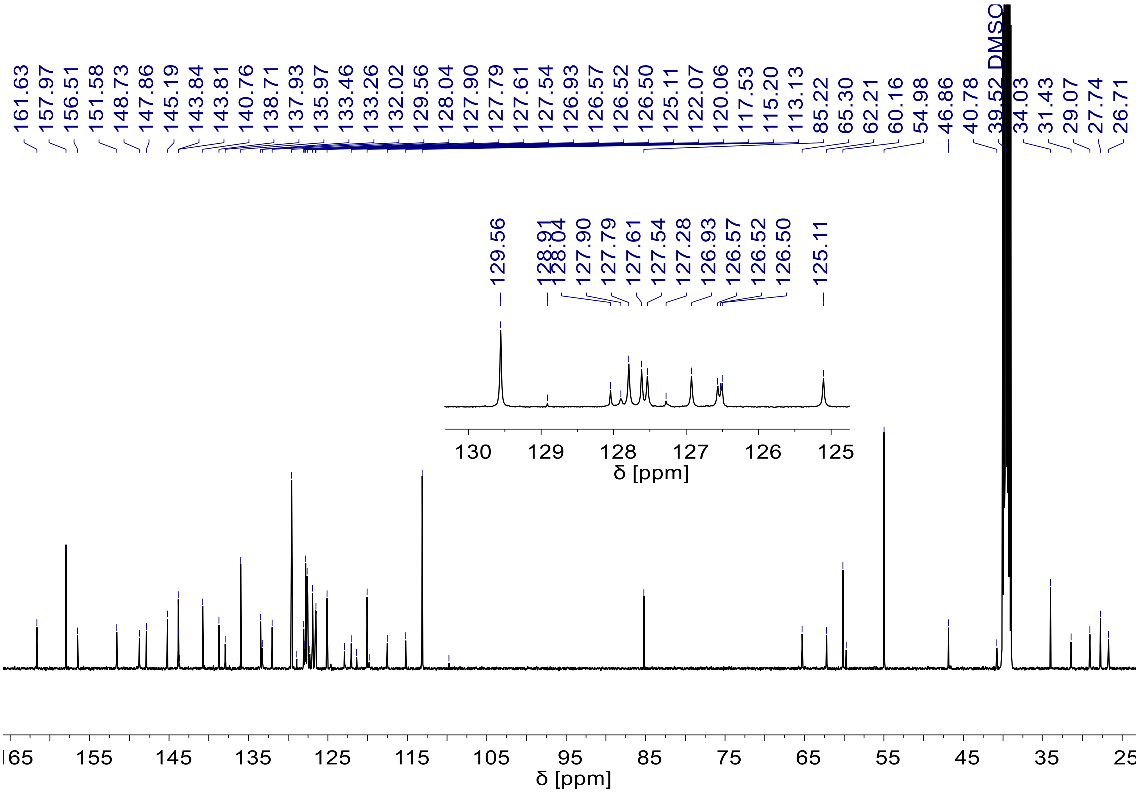

Figure S36 ^{13}C NMR spectrum of compound **12.**

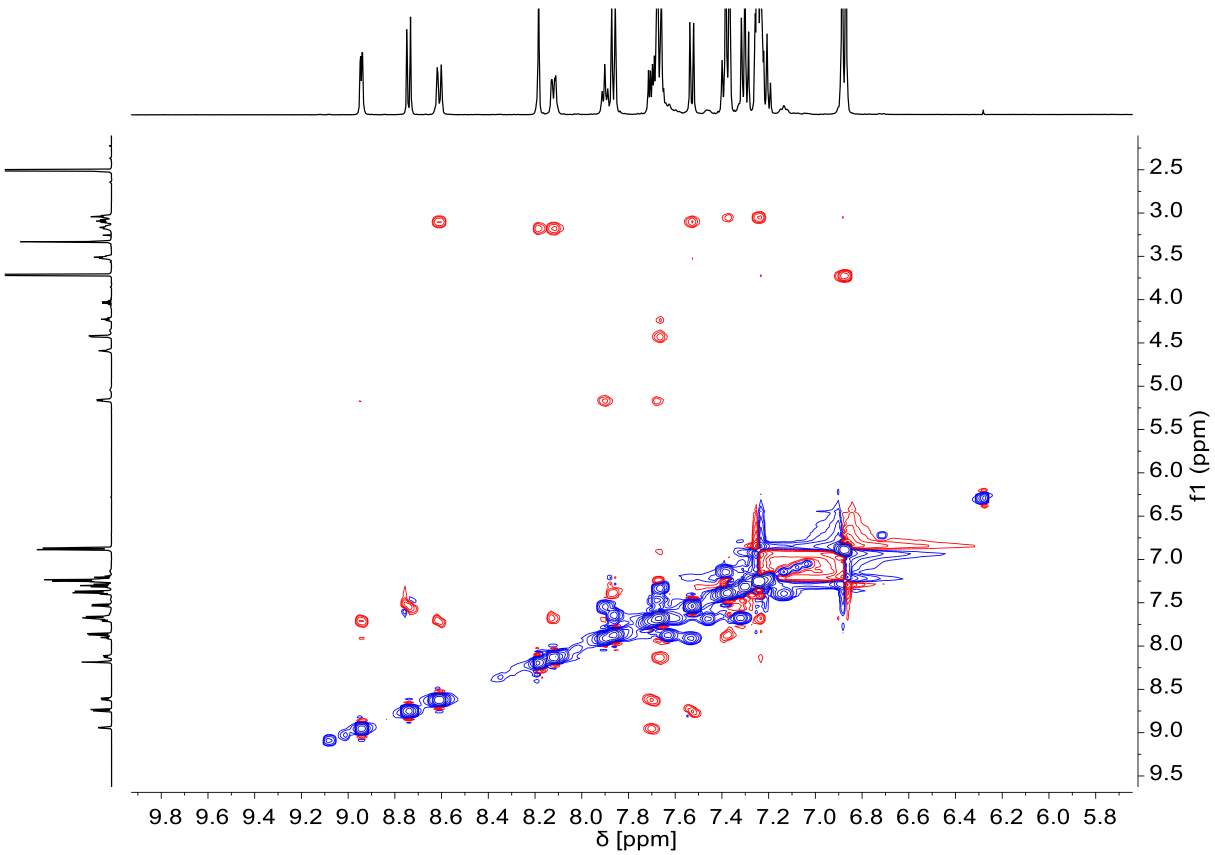

Figure S37 ROESY-EXSY NMR spectrum of compound **12.**

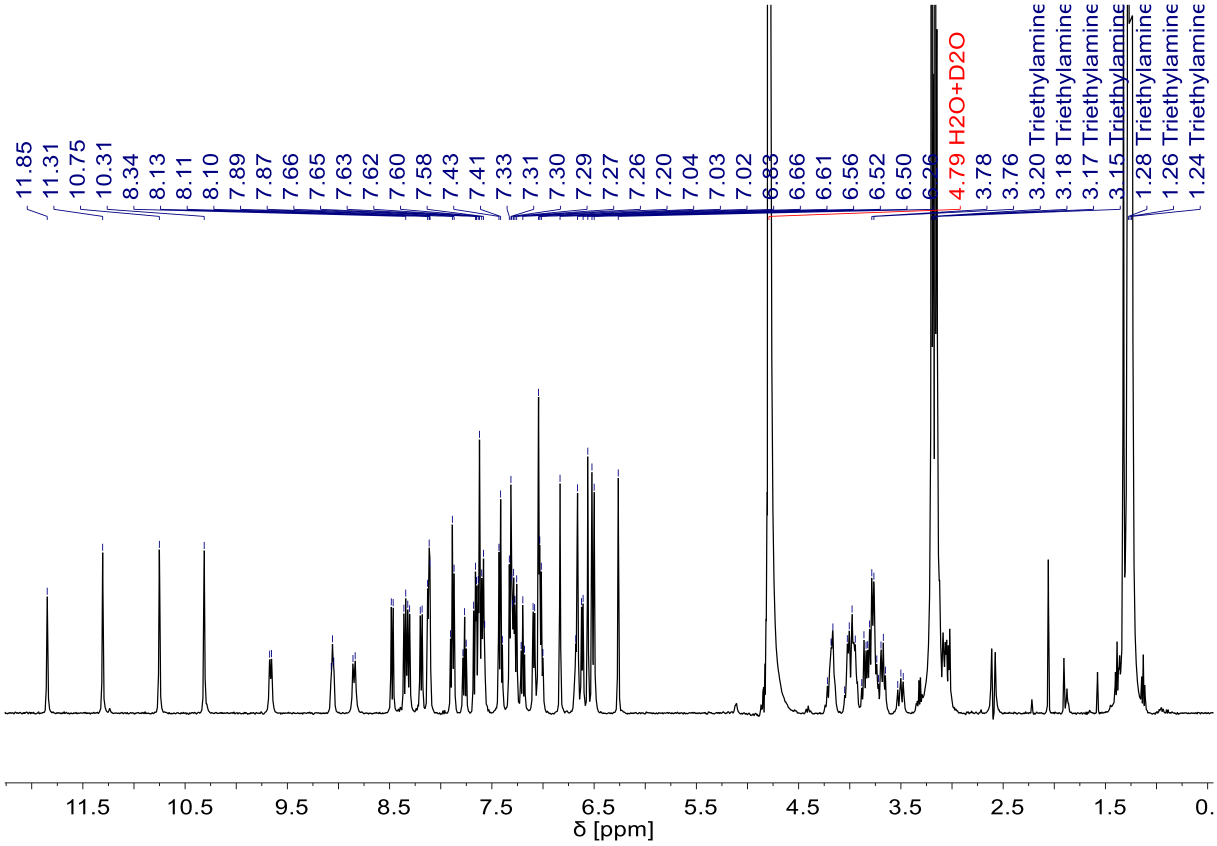

Figure S38 ^{1}H NMR spectrum of **22** in H_2O/ D_2O (v/v; 9:1; 50 mM NH_4HCO_3), water suppression.

4.2 HPLC chromatograms

4.2.1 Foldamer chromatograms

Compound **22a**

Gradient: 30-100 % B in A, 0.1% TFA buffer

Column: Nucleodur C18

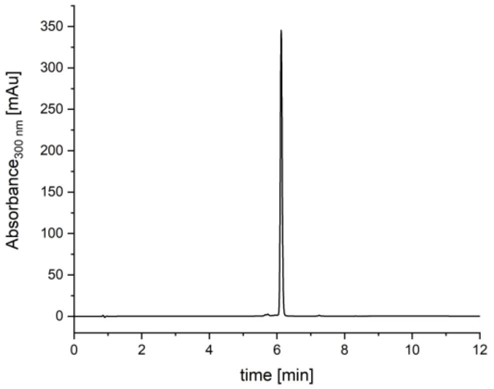

Compound **22**

Gradient: 0-100 % B in A, TEAA buffer

Column: Nucleodur C18

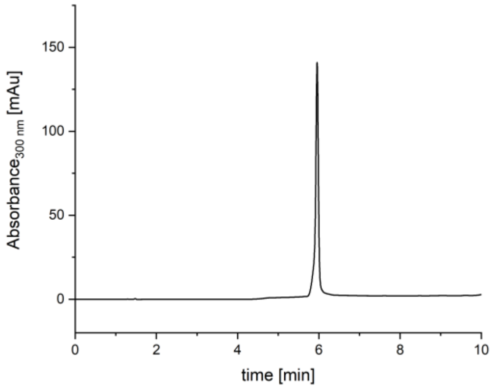

4.2.2 Oligonucleotide chromatograms

Compound **13**

D-(5’-GTT TTG **X**CA AAA C-3’)

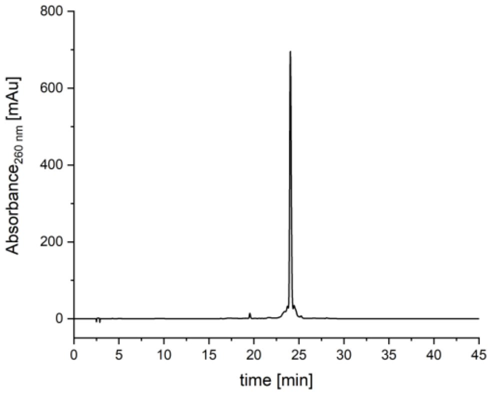

Compound **14**

L-(5’-GTT TTG **X**CA AAA C-3’)

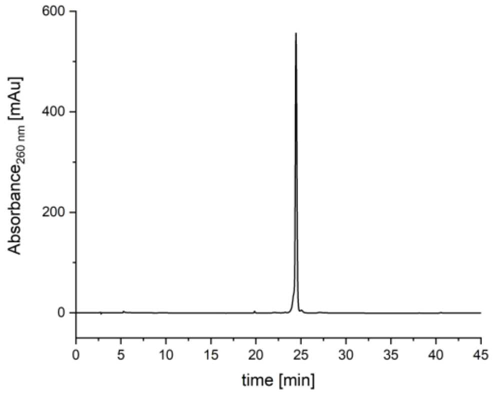

Compound **15**

5’-ACA GGA T**X**A TCC TGT-3’

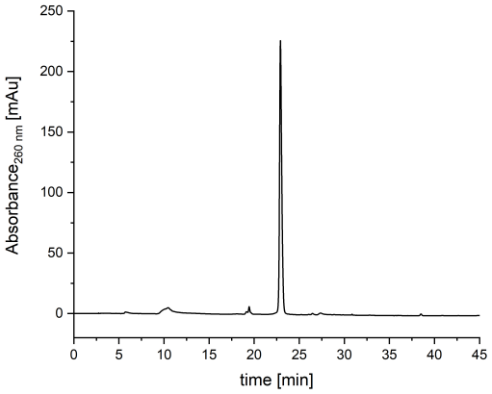

Compound **16**

5’-pC**X**G TCC TA-3’

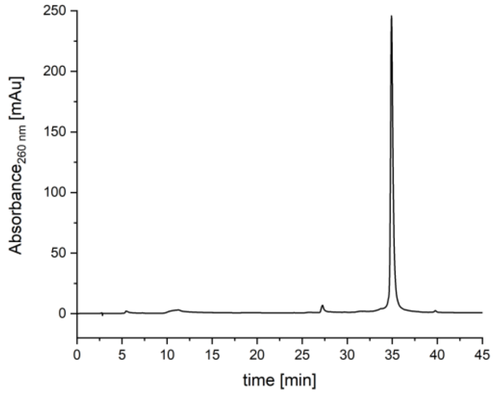

Compound **17**

5’-pAGG ATG **X**C-3’

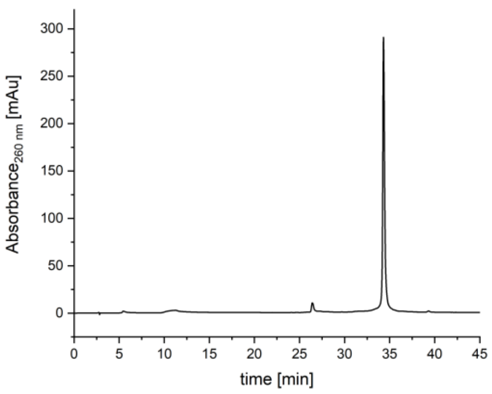

Compound **18**

5’-CTG **X**CA GGA TG**X** CAT Cp-3’

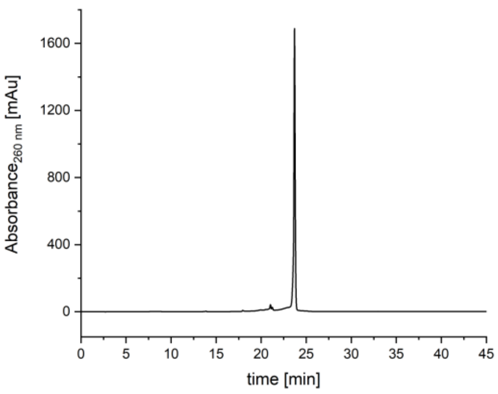

Compound **19**

5’-CTG T**X**A CAG GAT GT**X** ACA TCp-3’

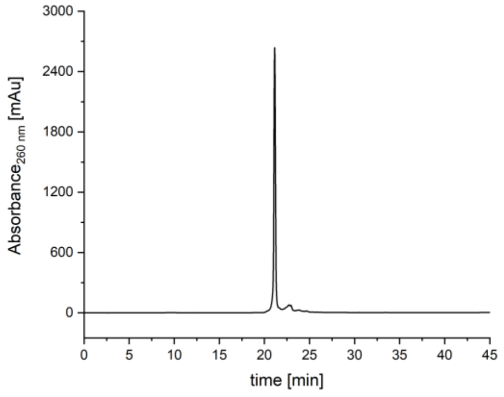

Compound **20**

5‘-CAC ATC CTG T**X(H)**A CAG GAT GTG-3‘

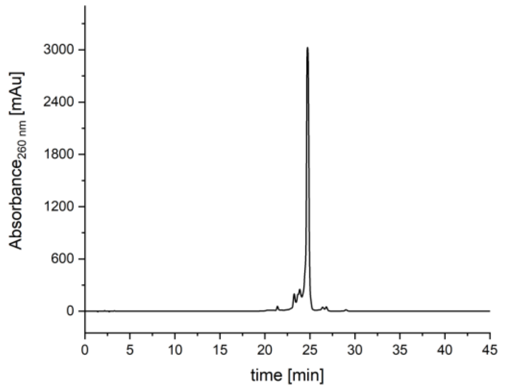

Compound **28**

5‘-CAC ATC CTG T**X(Bio)**A CAG GAT GTG-3‘

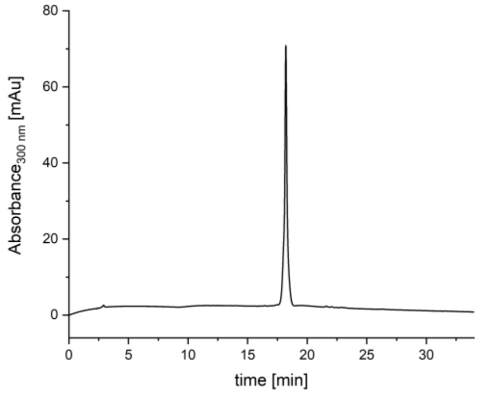

Compound **21**

5‘-GAC AGG ATG T**X(H)**A CAT CCT GTC-3

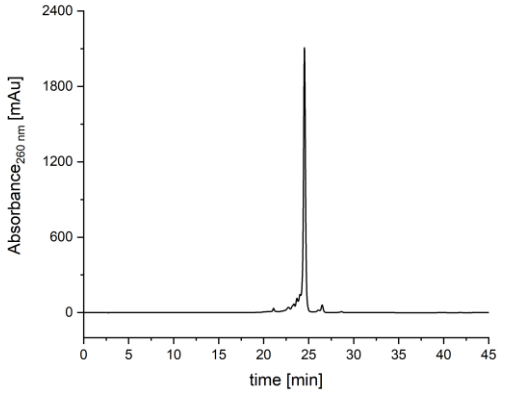

Compound **29**

5‘-GAC AGG ATG T**X(Bio)**A CAT CCT GTC-3

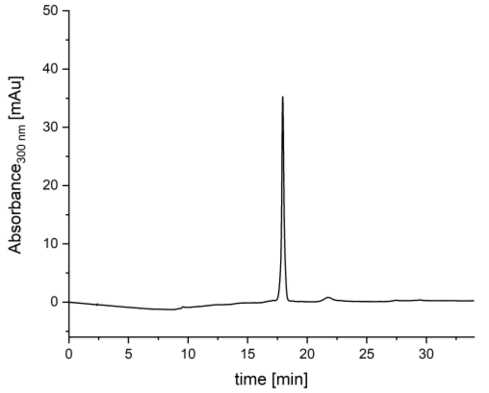

4.2.3 Conjugate chromatograms

Compound **25**

Gradient: 0-50 B in A; NH_4OAc buffer system

Column: Nucleodur C18

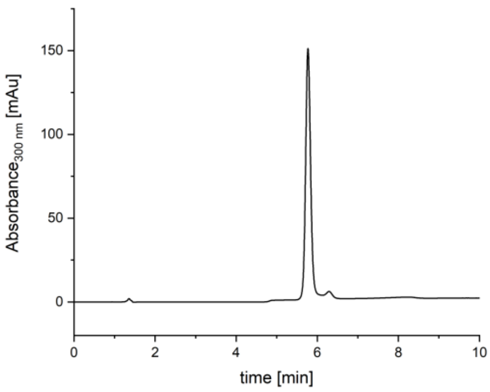

Compound **26**

Gradient: 0-50 B in A; NH_4OAc buffer system

Column: Nucleodur C18

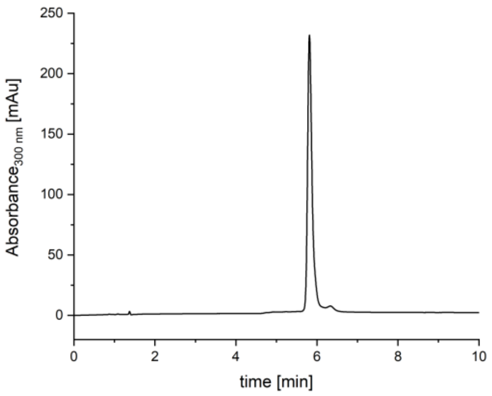

4.3 Mass spectra

4.3.1 Foldamer mass spectra

Compound **22a**

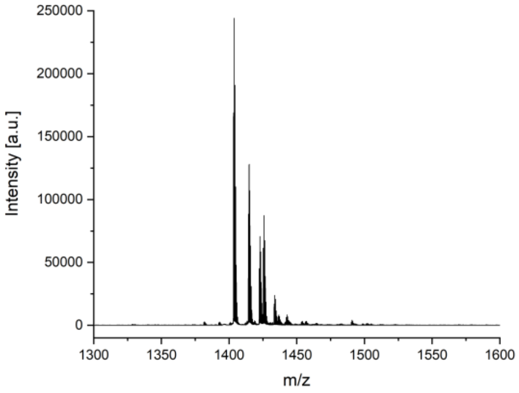

Compound **22a** Zoom

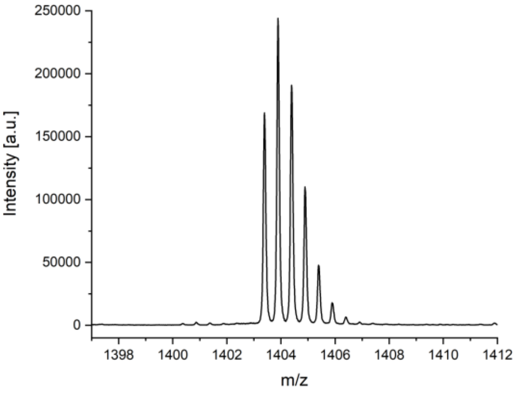

Compound **22**

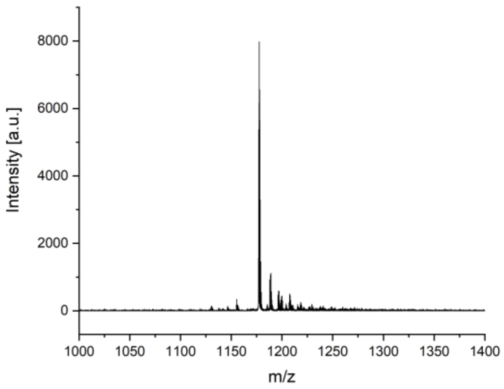

Compound **22** Zoom

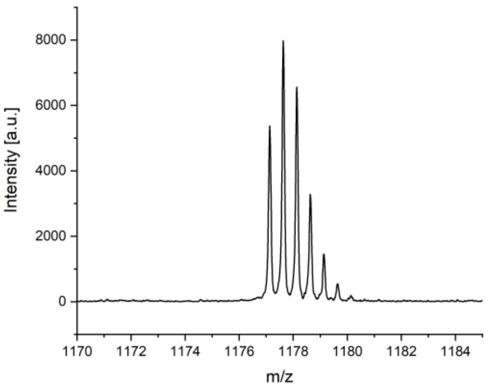

4.3.2 Oligonucleotide mass spectra

For conditions, see the general section for oligonucleotide synthesis

Compound **13**

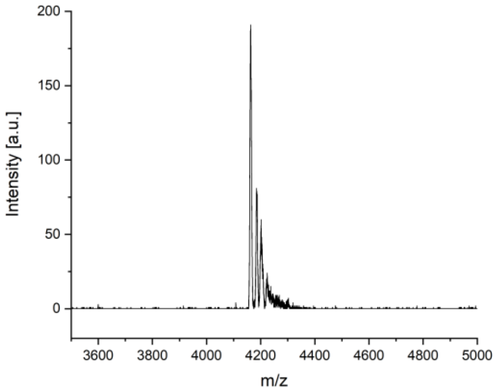

Compound **14**

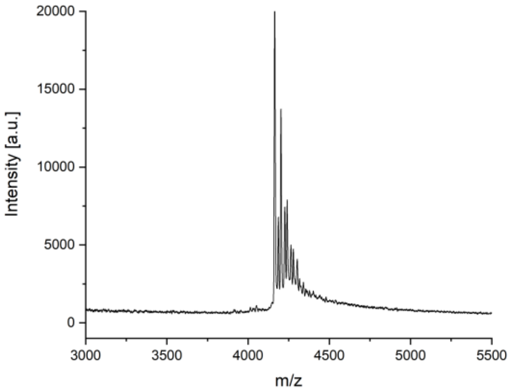

Compound **15**

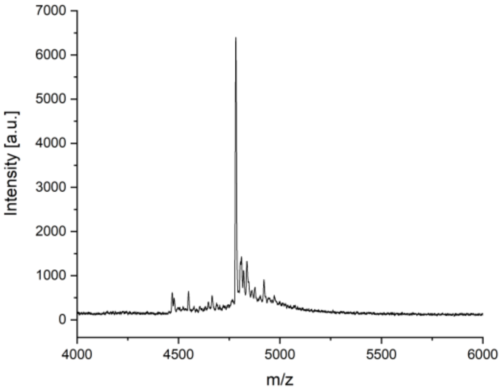

Compound **16**

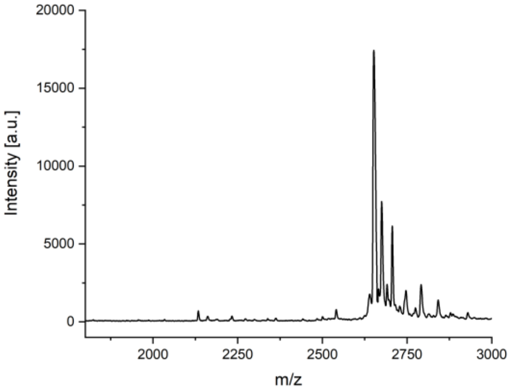

Compound **17**

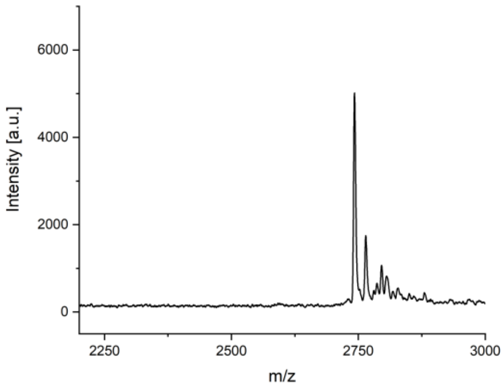

Compound **18**

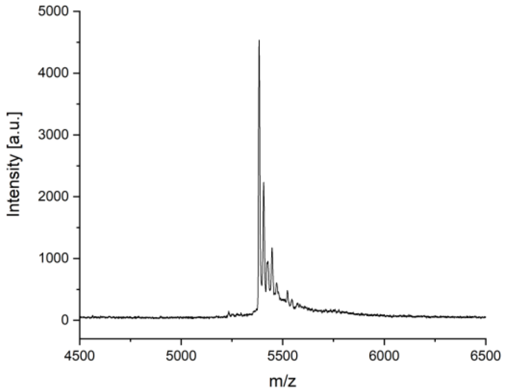

Compound **19**

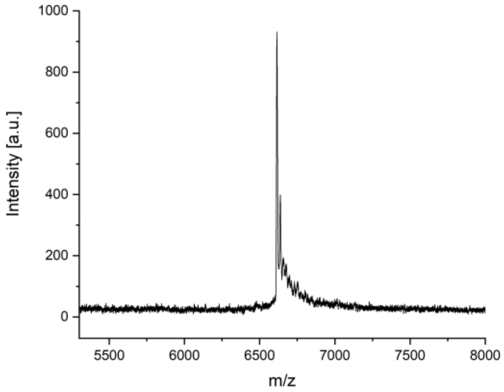

Compound **20**

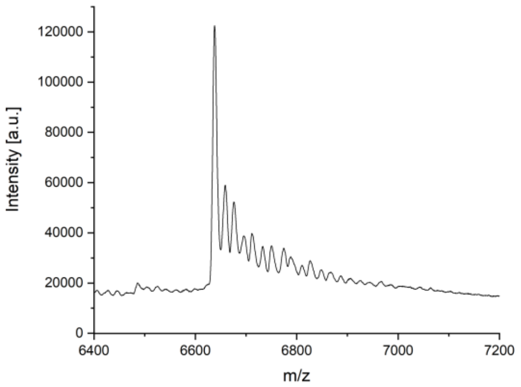

Compound **21**

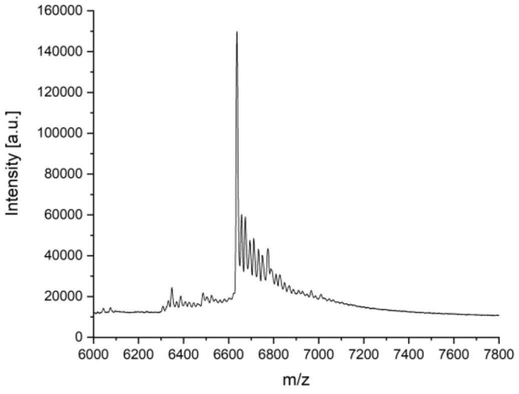

Compound **28**

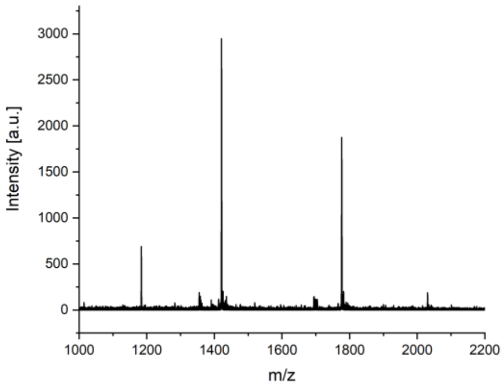

Compound **28** Zoom

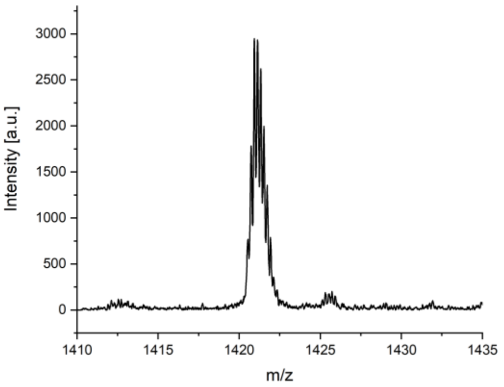

Compound **29**

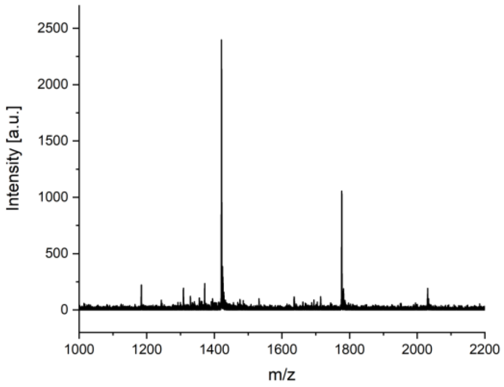

Compound **29** Zoom

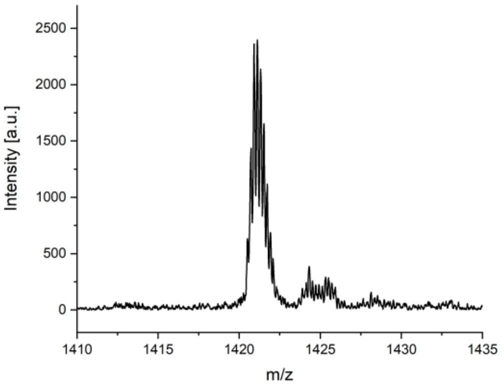

4.3.3 Conjugate mass spectra

Compound **24**

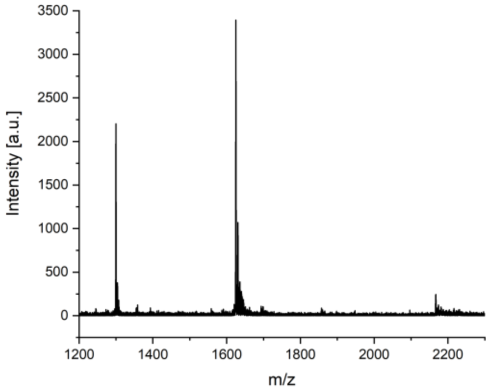

Compound **24** Zoom

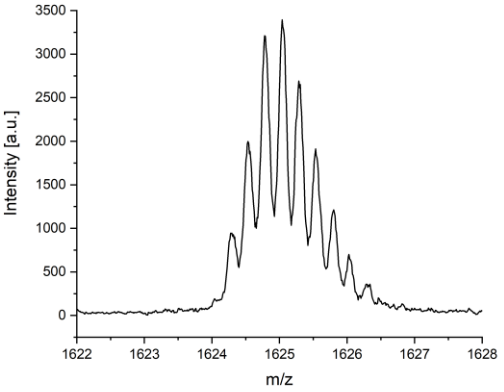

Compound **25**

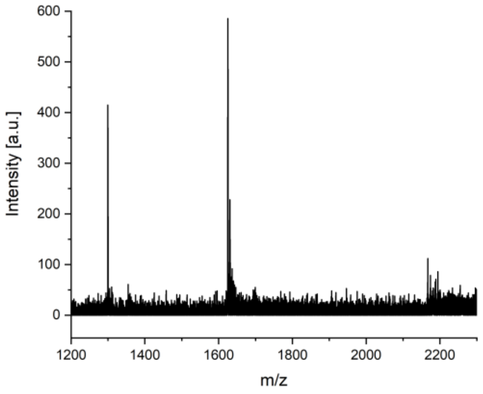

Compound **25** Zoom

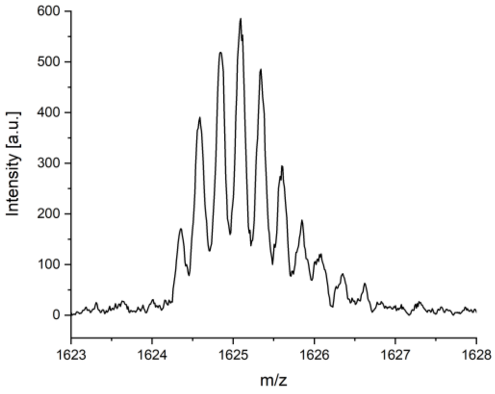

5 References

[1] K. Ziach, C. Chollet, V. Parissi, P. Prabhakaran, M. Marchivie, V. Corvaglia, P. P. Bose, K. Laxmi-Reddy, F. Godde, J.-M. Schmitter, S. Chaignepain, P. Pourquier, I. Huc, *Nat. Chem.* **2018**, *10*, 511-518.

[2] V. Corvaglia, J. Wu, D. Deepak, M. Loos, I. Huc, *Chem. - Eur. J.* **2024**, *30*, e202303650.

[23] M. Egli, V. Tereshko, G. N. Mushudov, R. Sanishvili, X. Liu, F. D. Lewis, *J. Am. Chem. Soc.* **2003**, *125*, 10842-10849.

[25] P. K. Mandal, G. W. Collie, B. Kauffmann, I. Huc, *Angew. Chem., Int. Ed.* **2014**, *53*, 14424-14427.

[28] E. Krissinel, K. Henrick, *Acta Crystallogr., Sect. D: Biol. Crystallogr.* **2004**, *D60*, 2256-2268.

[37] J. R. Terrell, S. J. Taylor, A. L. Schneider, Y. Lu, T. N. Vernon, S. Xhani, R. H. Gumpper, M. Luo, W. D. Wilson, U. Steidl, G. M. K. Poon, *Cell Rep.* **2023**, *42*, 112671.

[38] Y. Mo, B. Vaessen, K. Johnston, R. Marmorstein, *Mol. Cell* **1998**, *2*, 201-212.

[41] R. E. Franklin, R. G. Gosling, *Nature (London, U. K.)* **1953**, *172*, 156.

[42] G. R. Fulmer, A. J. M. Miller, N. H. Sherden, H. E. Gottlieb, A. Nudelman, B. M. Stoltz, J. E. Bercaw, K. I. Goldberg, *Organometallics* **2010**, *29*, 2176-2179.

[43] M. Cianci, G. Bourenkov, G. Pompidor, I. Karpics, J. Kallio, I. Bento, M. Roessle, F. Cipriani, S. Fiedler, T. R. Schneider, *J. Synchrotron Radiat.* **2017**, *24*, 323-332.

[44] P. Evans, *Acta Crystallogr., Sect. D: Biol. Crystallogr.* **2006**, *D62*, 72-82.

[45] P. R. Evans, G. N. Murshudov, *Acta Crystallogr., Sect. D: Biol. Crystallogr.* **2013**, *69*, 1204-1214.

[46] W. Kabsch, *Acta Crystallogr., Sect. D: Biol. Crystallogr.* **2010**, *66*, 125-132.

[47] C. Vonrhein, C. Flensburg, P. Keller, A. Sharff, O. Smart, W. Paciorek, T. Womack, G. Bricogne, *Acta Crystallogr., Sect. D: Biol. Crystallogr.* **2011**, *67*, 293-302.

[48] A. J. McCoy, R. W. Grosse-Kunstleve, P. D. Adams, M. D. Winn, L. C. Storoni, R. J. Read, *J. Appl. Crystallogr.* **2007**, *40*, 658-674.

[49] M. D. Winn, C. C. Ballard, K. D. Cowtan, E. J. Dodson, P. Emsley, P. R. Evans, R. M. Keegan, E. B. Krissinel, A. G. W. Leslie, A. McCoy, S. J. McNicholas, G. N. Murshudov, N. S. Pannu, E. A. Potterton, H. R. Powell, R. J. Read, A. Vagin, K. S. Wilson, *Acta Crystallogr., Sect. D: Biol. Crystallogr.* **2011**, *67*, 235-242.

[50] P. Emsley, B. Lohkamp, W. G. Scott, K. Cowtan, *Acta Crystallogr., Sect. D: Biol. Crystallogr.* **2010**, *66*, 486-501.

[51] M. D. Winn, G. N. Murshudov, M. Z. Papiz, *Methods Enzymol.* **2003**, *374*, 300-321.

[52] G. N. Murshudov, P. Skubak, A. A. Lebedev, N. S. Pannu, R. A. Steiner, R. A. Nicholls, M. D. Winn, F. Long, A. A. Vagin, *Acta Crystallogr., Sect. D: Biol. Crystallogr.* **2011**, *67*, 355-367.

[53] A. A. Vagin, R. A. Steiner, A. A. Lebedev, L. Potterton, S. McNicholas, F. Long, G. N. Murshudov, *Acta Crystallogr., Sect. D: Biol. Crystallogr.* **2004**, *D60*, 2184-2195.

[54] A. T. Bruenger, *Nature (London)* **1992**, *355*, 472.

[55] A. W. Schuettelkopf, D. M. F. van Aalten, *Acta Crystallogr., Sect. D: Biol. Crystallogr.* **2004**, *D60*, 1355-1363.

[56] A. Wilson, *Acta Crystallogr.,* **1950**, *3*, 397-398.

[57] H. M. Berman, J. Westbrook, Z. Feng, G. Gilliland, T. N. Bhat, H. Weissig, I. N. Shindyalov, P. E. Bourne, *Nucleic Acids Res.* **2000**, *28*, 235-242.

[58] W.L. Delano, 2002 The PyMOL Molecular Graphics System, Delano Scientific, San Carlos **2002**.

[59] X. -J. Lu, W. K. Olson, *Nat. Protoc.* **2008**, *3*, 1213-1227.

[60] Schrödinger, LLC. *Maestro*; Schrödinger, LLC: New York, NY, **2021**.

[61] N. Yang, X. Su, V. Tjong, W. Knoll, *Biosens. Bioelectron.* **2007**, *22*, 2700-2706.

[62] S. Wang, G. M. K. Poon, W. D. Wilson, *Methods Mol. Biol. (N. Y., NY, U. S.)* **2015**, *1334*, 313-332.

[63] C. Perez, A. M. Barkley-Levenson, B. L. Dick, P. F. Glatt, Y. Martinez, D. Siegel, J. D. Momper, A. A. Palmer, S. M. Cohen, *J. Med. Chem.* **2019**, *62*, 1609-1625.

[64] V. Corvaglia, F. Sanchez, F. S. Menke, C. Douat, I. Huc, *Chem. - Eur. J.* **2023**, *29*, e202300898.
